# Temporal vascular pattern remodeling mediated by the FHL2/sFRP2 signaling pathway in tenocytes affects tendon repair and regeneration

**DOI:** 10.1038/s12276-025-01574-2

**Published:** 2025-11-11

**Authors:** Qihang Su, Heng’an Ge, Jun Li, Centao Liu, Liyang Chen, Jie Li, Qiuchen Cai, Chenglong Huang, Xiaofei Feng, Dandan Li, Biao Cheng

**Affiliations:** 1 Department of Sports Medicine, Tongji Hospital, School of Medicine, Tongji University, Shanghai, China; 2 Department of Orthopedics, Shanghai Tenth People’s Hospital, School of Medicine, Tongji University, Shanghai, China; 3 Department of Sports Medicine, Huashan Hospital, Fudan University, Shanghai, China; 4 Department of Orthopedics, Zhabei Central Hospital of Jing’an District, Shanghai, China; 5 Department of Orthopedics, Ningbo No. 2 Hospital, Ningbo, China; 6 Department of Medical Ultrasound, Shanghai Tenth People’s Hospital, Ultrasound Research and Education Institute, Shanghai Engineering Research Center of Ultrasound Diagnosis and Treatment, School of Medicine, Tongji University, Shanghai, China; 7 Department of Biostatistics, Health Informatics, and Data Sciences, College of Medicine, University of Cincinnati, Cincinnati, OH USA

**Keywords:** Cell signalling, Trauma, Inflammation

## Abstract

Although angiogenesis following tendon injury was expected to provide nutrients for regeneration and repair, excessive angiogenesis may be associated with poor long-term outcomes in tendinopathy. Here we aim to explore the pathological role of angiogenesis in the progression of tendinopathy. Patients with tendinopathy were categorized into a hypervascularization group (HyperV) and a hypovascularization group (HypoV), and postarthroscopic outcome and histopathology were compared. In addiiton, tendon injury models and tenocyte stress models were employed to investigate the temporal–spatial vascular pattern characteristics and mechanisms involved in the progression of tendinopathy. This study finds that the HyperV group exhibited worse postoperative pain and functional outcomes and higher Bonar’s pathological scores and vascular density. Bulk RNA sequencing and pathological staining revealed that decreased FHL2 and increased YAP1/sFRP2 expression in tenocytes were strongly associated with disorganized tissue pathology, aggravated inflammation and increased vascular abundance in the HyperV group and tendon injury models (Td-Inj and Td-Sut groups). Three-dimensional vascular imaging demonstrated the formation of morphologically complex and abnormally distributed blood vessels in the Td-Inj and Td-Sut groups, which was significantly alleviated by *YAP1* knockdown. In activated tenocytes, FHL2 deficiency-mediated YAP1 overexpression led to the overexpression and extracellular secretion of sFRP2, thereby enhancing endothelial angiogenesis. *FHL2* overexpression partly mitigated vascular remodeling and improved tendon blood perfusion in rats. In summary, FHL2/YAP1/sFRP2-mediated pathological vascular remodeling disrupts the homeostasis of tendon repair and regeneration. This study underscores the importance of a systematic vascular assessment, incorporating abundance, morphology, and spatial distribution, in tendinopathy.

## FHL2 YAP1 signaling disrupts tendon repair through angiogenesis

Tendinopathy is a condition where tendons become damaged, causing pain and dysfunction. This study explores how blood vessel growth, or angiogenesis, affects tendinopathy. Researchers found that excessive blood vessel growth in tendons can worsen the condition rather than help it heal. The study involved patients with chronic rotator cuff tendinopathy and used rat models to simulate tendon injuries. Researchers focused on three proteins: FHL2, YAP1 and sFRP2. They discovered that low levels of FHL2 and high levels of YAP1 and sFRP2 are linked to abnormal blood vessel growth and poor tendon healing. By increasing FHL2 levels in injured tendons, they improved tissue structure and blood flow, suggesting a potential treatment strategy. The study concludes that managing blood vessel growth in tendons could lead to better treatments for tendinopathy.

This summary was initially drafted using artificial intelligence, then revised and fact-checked by the author.

## Introduction

Tendinopathy consists of a spectrum of changes in damaged and diseased tendons, leading to pain and dysfunction, with its prevalence increasing steadily^1^. Impaired tendon repair can induce the progression of chronic tendinopathy, resulting in suboptimal pain relief and functional recovery, while the high cost-effectiveness ratio increases socioeconomic burdens^2,3^. Tendons are hypovascular tissues, and neovascularization after tendon injury provides oxygen supply and nutrients for regeneration and repair^4–6^; however, increased vascular abundance in tendinopathy could not improve long-term outcomes^7,8^. Tendinopathy-associated neovascularization appears positively correlated with the severity of pain^9–11^, and inhibition of angiogenesis was shown to alleviate pathological scar formation^12^ and prevent the progression of heterotopic ossification^13^. Although angiogenesis plays a critical role in tendon injury repair and tendinopathy progression, its physiological and pathological mechanisms remain poorly understood. Therefore, the effect of angiogenesis on disease prognosis has remained controversial in therapeutic exploratory studies, with limited evidence^14^. Revisiting the effects of vascular networks on tendinopathy holds vital importance and value.

Under the intense metabolic demands of tissue repair, neovascularization may occur as an exaggerated response. Subsequent vascular pruning reduces unnecessary vessels and promotes maturation (physiological remodeling), achieving vascular bed homeostasis. However, in tendinopathy, this neovascularization-regression equilibrium appears disrupted. Immature, hyperpermeable vessels may persist, failing to resolve hypoxia while simultaneously perpetuating abnormal vascular proliferation, which further exacerbates local oxygen and nutrient consumption^15^. Motivated by the rationale of inhibiting this excessive neovascularization, early studies proposed using sclerosing agents to directly ablate nascent vessels as a means to alleviate tendinopathy pain^16^. Critically, however, a mid-term follow-up study (2.7–5.1 years; mean, 3.9 years) found that this vasculature-targeted inhibitory approach failed to provide important long-term benefits for patients with mid-portion tendinopathy^17^. This strategy of solely suppressing neovascularization seems counterintuitive to the fundamental role of angiogenesis in supporting tissue repair and regeneration. More importantly, its lack of efficacy strongly suggests that the vascular pathology in tendinopathy extends beyond mere vessel ‘excess’ and is fundamentally rooted in ‘remodeling failure’ leading to homeostatic imbalance. This therapeutic dilemma is not unique to tendinopathy: recent research in highly vascularized pathologies such as retinopathies and tumors has revealed that clinically used antiangiogenic drugs can sometimes exacerbate underlying ischemia by excessively inhibiting vasculature. Conversely, strategies aimed at transiently modulating rather than completely suppressing angiogenesis—thereby promoting vascular structural/functional ‘normalization’—have been shown to improve perfusion and yield superior therapeutic outcomes^18–20^. Collectively, these insights across diverse fields highlight a critical principle: in pathologies reliant on vascular repair, simply blocking neovessel formation is often counterproductive; restoring a healthy, functional vascular network homeostasis (that is, effective physiological remodeling) is paramount. Consequently, Järvinen et al. propose that shifting the therapeutic focus in tendinopathy from inhibiting angiogenesis toward promoting vascular bed homeostasis and rectifying pathological remodeling represents a potentially more promising strategy^21^. Nevertheless, achieving this goal requires a more profound elucidation of the specific pathological features of vascular remodeling in tendinopathy and the key factors that disrupt the neovascularization-pruning balance.

Traditionally, studies on vascular remodeling in tendinopathy have primarily focused on changes in vascular abundance. However, recent studies have revealed that the spatial morphology and distribution of blood vessels also play a critical role in disease prognosis^22–26^. Single-cell and spatial transcriptomic mapping^27^ demonstrated critical spatial heterogeneity in endothelial cell phenotypes in tendinopathy. Endothelial cells exhibit spatial behaviors, such as migration, bypassing and redeployment through phenotypic transitions, which can drive vascular spatial remodeling independently of apoptosis^28^. However, the characteristics of vascular spatial patterns, such as vascular abundance, morphology and distribution, have not yet been elucidated in tendinopathy. A systematic assessment of vascular patterns is crucial to provide a deeper insight into the pathogenesis of tendinopathy.

This study first analyzed the clinical outcomes and histological characteristics of patients with tendinopathy and hypervascular or hypovascular signals (power Doppler imaging) to identify key pathological factors. Using rat tendon injury/tendinopathy models, we then systematically characterized vascular spatial patterning during tendon repair and tendinopathy progression. We found that reduced FHL2 (four and a half LIM domains protein 2, an adapter and modifier in protein interactions), elevated YAP1 (Yes-associated protein 1, a core transcriptional co-activator in the Hippo pathway regulating organ size and tissue regeneration) and increased sFRP2 (secreted frizzled-related protein 2, an extracellular secreted protein) are closely associated with pathological vascular remodeling in tendinopathy. In vitro studies further elucidated underlying mechanisms. Finally, adenoassociated virus (AAV) vectors were employed to explore the feasibility of *FHL2* gene therapy for reversing vascular remodeling in tendinopathy. We hypothesized that vascular pattern remodeling may represent a critical pathological factor influencing tendon regeneration and repair, with FHL2, YAP1 and sFRP2 playing pivotal roles in this process. This study aims to provide novel insights and theoretical foundations for promoting vascular bed homeostasis in the treatment of tendinopathy.

## Materials and methods

### Clinical study

#### Patient cohort and clinical follow-up

Patients with chronic rotator cuff tendinopathy and associated rotator cuff injuries were included in this clinical study. The symptoms of participants persisted for at least 12 months, and all patients underwent arthroscopic rotator cuff repair combined with shoulder joint debridement between October 2020 and October 2021. Based on the Adler grading^29^ of rotator cuff power Doppler imaging (Aplio 500, Toshiba), patients with chronic tendinopathy and grade II–III blood flow signals were categorized as the ‘hypervascularization’ group (HyperV group), whereas those with grade 0–I blood flow signals were categorized as the ‘hypovascularization’ group (HypoV group). In addition, patients undergoing anterior cruciate ligament reconstruction surgery (using autologous hamstring tendons) without rotator cuff pathology were recruited as the normal group, with their normal rotator cuff power Doppler imaging and hamstring tendons serving as imaging and sample controls, respectively. Detailed inclusion and exclusion criteria are provided in Supplementary Data File 1.1.

Clinical follow-up and assessments were conducted jointly by the first author and senior attending surgeons. Follow-up was conducted preoperatively and 1, 3, 6 and 12 months postoperatively. Follow-up assessments included the visual analog scale (VAS) for shoulder joint pain during daily active movements, the modified University of California at Los Angeles (UCLA) shoulder function score^30^ and the upper limb strength and resistance time (testing methods detailed in Supplementary Data File 1.1).

#### Sampling and histological assessment

During arthroscopic rotator cuff repair or anterior cruciate ligament reconstruction, tendon tissues were obtained from the rotator cuffs of the HypoV and HyperV groups and the hamstring tendons of the normal group. Western blotting (WB) and routine pathological staining were conducted (detailed methods are provided in Supplementary Data File 1.2). Regarding pathological staining, we conducted hematoxylin–eosin staining, Alcian Blue staining, Sirius Red staining (SR), TdT-mediated dUTP nick-end labeling assay (TUNEL), immunohistochemistry (IHC) and immunofluorescence (IF). The histological structure of the tendon was evaluated using modified Bonar’s scoring^31^ combined with hematoxylin–eosin, Alcian Blue staining and SR staining.

#### Bulk RNA-seq

RNA sequencing analysis was conducted on rotator cuff tissues from the HypoV group (7 samples) and the HyperV group (12 samples) to identify major differentially expressed genes (DEGs) and enriched pathways. The sequencing was supported by Shanghai Jiayin Biotechnology. The DEGs were defined as genes with adjusted *P* value <0.05 and |log_2_FC| >1. Principal component analysis, Gene Ontology (GO)/Kyoto Encyclopedia of Genes and Genomes (KEGG) functional enrichment, gene set enrichment analysis (GSEA) and protein interaction networks were conducted using relevant R packages, and the results were visualized.

### In vivo studies

#### Rats and sampling

Twelve-week-old male Sprague–Dawley rats were obtained from Shanghai Jihui Laboratory Animal Care (specific pathogen-free). Six-week-old rats were used for *YAP1*-knockdown/overexpression model construction. Rats were housed under controlled conditions (22 °C, 12-h light–dark cycle) with ad libitum access to water and standard laboratory rat chow. Each group included at least six rats per time point. All anesthesia procedures (modeling, sampling and euthanasia) were conducted using intraperitoneal injections of 2.5% avertin solution (M2820, Nanjing Aibei Biotechnology) at a dose of 6 ml/kg body weight for rats weighing less than 300 g and 7 ml/kg for rats weighing more than 300 g. For surgical sampling, we primarily collected intact Achilles tendon tissues, and the rats were killed via cervical dislocation at the end of the experiments.

#### Modeling of exercise-induced tendon injury

A treadmill exercise-induced tendon injury model was employed to minimize the effect of external physical interventions and closely mimic the pathogenesis of tendon degeneration. Based on previous studies^32–36^, this study established low-intensity exercise protocols (representing a lower intensity to induce tendon injury changes) and high-intensity exercise protocols (representing the maximum tolerable intensity for rats). Experimental groups were divided into a control group (Control), a low-intensity treadmill group (L-tm) and a high-intensity treadmill group (H-tm) to explore the temporal pathological characteristics and vascular features of tendon injury under varying exercise intensities and durations. After completing the exercise protocols, tendon pathological structure, cell proliferation/apoptosis, vascular abundance and the expression of key signaling factors were assessed at 6, 8, 12 and 18 weeks. Detailed exercise protocols are provided in Supplementary Data File 2.2.

#### Modeling of trauma-induced tendon injury

A trauma-induced tendon injury rat model was constructed by partly severing tendons to simulate clinical tendon tears and investigate the temporal pathological characteristics of acute tendon injury progressing to chronic tendinopathy. This model provides controlled injury severity, high reliability and reproducibility. In addition, a modified Kessler suture technique^37^ was used to repair injured tendons, simulating the clinical treatment of tendon sutures to explore the efficacy of suturing in improving pathological remodeling. The study included three groups, including sham surgery (Sham), tendon injury (Td-Inj) and tendon suture (Td-Sut). Postoperative assessments, including in vivo three-dimensional vascular imaging and tissue sampling, were conducted on day 3, and at 1, 2, 4 and 6 weeks. Systematic assessment included the pathological changes of tendon tissue, cell proliferation/apoptosis, matrix remodeling (collagen types, matrix metalloproteinases (MMPs) and inflammation), vascular patterns (in vivo three-dimensional vascular imaging) and the expression of key signaling factors. Detailed modeling methods are provided in Supplementary Data File 2.3.

#### Modeling of *YAP1*-knockdown/overexpression tendon injury

This study utilized AAV vectors for transfection (localized Achilles tendon injections) and surgery to construct tendon injury models with *YAP1* gene overexpression or knockdown and unravel the specific regulatory mechanisms. Six-week-old rats were randomly assigned to four groups: control transfection group (Ctl/*NC*), control transfection tendon injury group (Inj/*NC*), *YAP1*-knockdown tendon injury group (Inj/*YAP1*^*KD*^) and *YAP1* overexpression tendon injury group (Inj/*YAP1*^*OE*^). Assessments were conducted at 3 days, 1 week, 2 weeks, 4 weeks and 6 weeks after modeling. We evaluated the pathological changes of the tendon tissue (pathological staining), vascular patterns (in vivo three-dimensional vascular imaging) and cellular expression of key signaling factors. Detailed modeling methods are provided in Supplementary Data File 2.4 and Supplementary Table 1.

#### In vivo microvascular three-dimensional imaging

In vivo, microvascular three-dimensional imaging of the Achilles tendon was conducted using an optical coherence tomography angiography (OCTA) system (Beijing HealthOLight Technology). Rats were anesthetized, shaved, prepped and fixed in their position. The skin along the previous modeling incision was carefully opened to expose the Achilles tendon and minimize the vascular damage. OCTA imaging of the Achilles tendon was then conducted. The obtained data were used for three-dimensional vascular reconstruction, and regions of interest in the Achilles tendon were selected to analyze the vascular patterns. We analyzed the following parameters: (1) vessel diameter index (VDI), representing the average vessel diameter calculated from the vascular area and skeleton vessel maps; (2) vessel area density (VAD), the ratio of vascular area to the total image area in binarized vascular images; and (3) vessel complexity index (VCI), reflecting the complexity of vascular morphology based on perimeter and vascular area maps.

#### TEM

Transmission electron microscope (TEM) was used to assess collagen fibers in the tendons of the Sham, Td-Inj and Td-Sut groups 6 weeks after the surgery. After standard TEM section staining, ImageJ software was used to calculate and analyze the collagen volume fraction (%volume fraction = collagen area/total area) and the mean fibril diameter (Feret’s diameter, nanometers) in cross-sectional images of the tendon mid-substance. Detailed TEM procedures are provided in Supplementary Data File 2.5.

#### Histopathological assessment

Hematoxylin–eosin, SR, TUNEL, Masson’s trichrome staining (Masson), Safranin O-Fast green staining, IHC and IF were conducted for the histopathological staining of rat tendons. Furthermore, a semi-quantitative analysis of key protein expression was conducted using WB. Detailed procedures are described in Supplementary Data File 2.6.

### In vitro studies

#### Activated tenocyte models

Primary human tenocytes were cultured using 10% fetal bovine serum and DMEM/F12 complete medium under standard conditions. In vivo studies identified interleukin-1 beta (IL-1β, HY-P7028, MedChemExpress) as the major inflammatory factor in chronic tendinopathy, and we detected significantly elevated levels of transforming growth factor-beta 1 (TGF-β1, HY-P7118, MedChemExpress). Therefore, IL-1β and TGF-β1 were used as primary stimulatory factors to construct activated tenocyte models. In addition, *tert*-butyl hydroperoxide (tBHP, no. 458139, Sigma-Aldrich), a commonly used oxidative stress inducer^38^, was used to establish oxidative stress models of tenocytes.

Tenocytes were treated with IL-1β (0.5/1/2.5/5/10/15/20 ng/ml), tBHP (0.05/0.1/0.25/0.5/1/2 μM) or TGF-β1 (0.1/0.5/1/2/4/8/10/15/20/25 ng/ml) in various concentration gradients. Cell viability was assessed using the Cell Counting Kit-8 cell proliferation assay (CCK8, G4103, Servicebio) at 1, 6, 12, 24, 48, 72 and 96 h. The optimal treatment concentrations (IL-1β: 2.5 ng/ml; tBHP: 0.1 μM; TGF-β1: 4 ng/ml) and treatment durations (12 or 24 h) were selected to measure the expression of the relevant proteins.

#### siRNA/plasmid-treated tenocyte models

Using the activated tenocyte model, we conducted siRNA transfection with negative control-siRNA (*siNC*), *YAP1*-siRNA (*siYAP1*) and four and a half LIM domains protein 2-siRNA (*siFHL2*), as well as plasmid transfection with an empty vector (Vectors) and *FHL2* overexpression plasmid (*FHL2*^*OE*^). WB and flow cytometry-based apoptosis assays were used to measure the regulatory mechanisms. Detailed methods are provided in Supplementary Data File 3.1 and Supplementary Table 2.

#### ELISA

Enzyme-linked immunosorbent assay (ELISA) kits were used to detect secreted frizzled-related protein 2 (sFRP2) levels in the culture medium of tenocytes treated with IL-1β, tBHP and TGF-β1 after *siNC/siYAP1* transfection to assess the extracellular levels of sFRP2. Detailed methods are provided in Supplementary Data File 3.2.

#### sFRP2-treated HUVECs

This section explores the effects of sFRP2 on human umbilical vein endothelial cells (HUVECs, HUVEC-20001 and OriCell) in terms of proliferation, apoptosis, migration and angiogenesis.

First, a concentration gradient of recombinant human sFRP2 protein (2.5 pM, 5 pM, 10 pM, 20 pM, 500 pM, 5 nM, 15 nM, 30 nM and 90 nM; CSB-MP021139HU, CUSABIO) was applied to HUVECs. Cell viability was measured using the CCK8 assay at 24, 48, 72 and 96 h to determine the optimal concentration for subsequent experiments.

Next, HUVECs were divided into the following groups for further assessments: NC group (negative control, untreated), 7 pM-sFRP2 treatment group, 7 pM-sFRP2 + U73122 (1 μM, phospholipase C inhibitor, HY-13419, MedChemExpress) group, 7 pM-sFRP2 + Ceapin-A7 (30 μM, selective ATF6α signaling blocker, HY-108434, MedChemExpress) group and 7 pM-sFRP2 + Fz7-21 (30 μM, FZD7 receptor peptide antagonist, HY-P1454, MedChemExpress) group. We measured cell viability (CCK8), apoptosis/cell cycle (flow cytometry), migration (scratch wound healing assay) and angiogenesis (tube formation assay) of HUVECs.

#### Tenocyte-HUVECs coculture models

Coculture models of normal or *sFRP2*-knockdown (*sFRP2*^*KD*^) tenocytes with HUVECs were established using Transwell chambers. The angiogenic ability of HUVECs was measured after treatment with IL-1β (2.5 ng/ml). Detailed methods are provided in Supplementary Data File 3.3.

#### Flow cytometry apoptosis/cell cycle assays

Flow cytometric apoptosis assay was conducted using Annexin V-FITC/PI apoptosis detection kits (C1062L, Beyotime), and the proportions of early and late apoptotic cells were analyzed. Cell cycle analysis was conducted using PI staining kits (C1052, Beyotime), and the proportions of cells in the G0/G1, S and G2/M phases were analyzed.

Flow cytometric apoptosis analysis of tenocytes was conducted at 24 h. The following groups were included: Vectors, IL-1β/tBHP/TGF-β1 + Vectors, *siYAP1*, IL-1β/tBHP/TGF-β1 + *siYAP1*, *FHL2*^*OE*^ and IL-1β/tBHP/TGF-β1 + *FHL2*^*OE*^. For HUVECs, both apoptosis assay and cell cycle analysis were conducted at 12, 24 and 48 h. The following groups were included: NC, sFRP2 treatment, sFRP2 + U73122, sFRP2 + Ceapin-A7 and sFRP2 + Fz7-21.

#### Scratch wound healing assay

Scratch wound healing assays were used to determine HUVEC migration rates in the NC, sFRP2 treatment, sFRP2 + U73122, sFRP2 + Ceapin-A7 and sFRP2 + Fz7-21 groups at 0, 12, 24 and 48 h. Detailed methods are provided in Supplementary Data File 3.4.

#### Tube formation assay

The tube formation assay was conducted to measure the angiogenic capacity of HUVECs in the NC, sFRP2 treatment, sFRP2 + U73122, sFRP2 + Ceapin-A7 and sFRP2 + Fz7-21 groups at 12, 24 and 48 h. HUVEC suspensions were collected, and the cells were prepared at a concentration of 4 × 10^4^ cells per milliliter in a serum-free medium with appropriate drug concentrations based on the experimental groups. Each well of a prechilled angiogenic µ-slide (ibidi) was coated with 10 μl matrigel (BD Biosciences). After gel solidification, 50 μl of the prepared cell suspension was added to each well. Photographs were taken at 12, 24 and 48 h, and ImageJ software was employed to analyze the number of branches, total branching length, number of junctions and number of meshes.

#### WB analysis

For in vitro experiments, adherent cells were detached from culture flasks using trypsin to collect cell pellets. The total protein was extracted and incubated with radioimmunoprecipitation assay buffer and protease inhibitors. WB was conducted as described in Supplementary Data File 1.2. The primary antibodies were as follows: FHL2 (TD13015, Abmart), YAP1 (A19134, ABclonal), p-YAP1^S127^ (TA3328, Abmart), sFRP2 (TD4451, Abmart), type I collagen (Col-I, ab138492, Abcam), type III collagen (Col-III, TA5457, Abmart), vascular endothelial growth factor A (VEGFA, TA5131, Abmart) and glyceraldehyde-3-phosphate dehydrogenase (GAPDH, GB15002, Servicebio).

### Therapeutic study

#### AAV-FHL2^OE^ therapeutic intervention in the rat models

Trauma-induced tendon injury models were established following the methods described in Supplementary Data File 2.3 to measure the therapeutic effects of *FHL2* overexpression on tendinopathy. Thereafter, AAV-*EGFP-FHL2*^*OE*^ transfection (Supplementary Table 1) was conducted, with in vivo OCTA, vascular perfusion and histopathological assessments at 6, 8 and 10 weeks after transfection. Detailed methods are provided in Supplementary Data File 4.1.

#### Assessment of vascular perfusion function

Rats were anesthetized, fixed and shaved for preparation. The mean blood flow in the Achilles tendon region was measured using laser speckle contrast imaging (LSCI, Jiangxi Zvast-Biotechnology). Four rats per group were assessed at each time point.

### Statistical analysis

The data were analyzed using IBM SPSS Statistics V20 software (IBM Corp.), considering a significance threshold of *P* < 0.05. Shapiro–Wilk or Kolmogorov–Smirnov was conducted to assess the normality of data, and a Levene’s test was conducted to measure the homogeneity of variance. Parametric or nonparametric tests were selected based on data characteristics. For data with a *P* value greater than 0.05 in the normality or homogeneity test and different results in the parametric and nonparametric tests, we primarily used the parametric test results. In the clinical study, VAS and UCLA scores were analyzed using a two-factor repeated-measures analysis of variance. For data with a two-factor factorial design involving group and time, further simple effect analysis was conducted when an interaction effect between group, and time was detected.

## Results

### Tendinopathy with hypervascularization exhibited poorer clinical prognosis compared with tendinopathy with hypovascularization

**Fig. 1:**
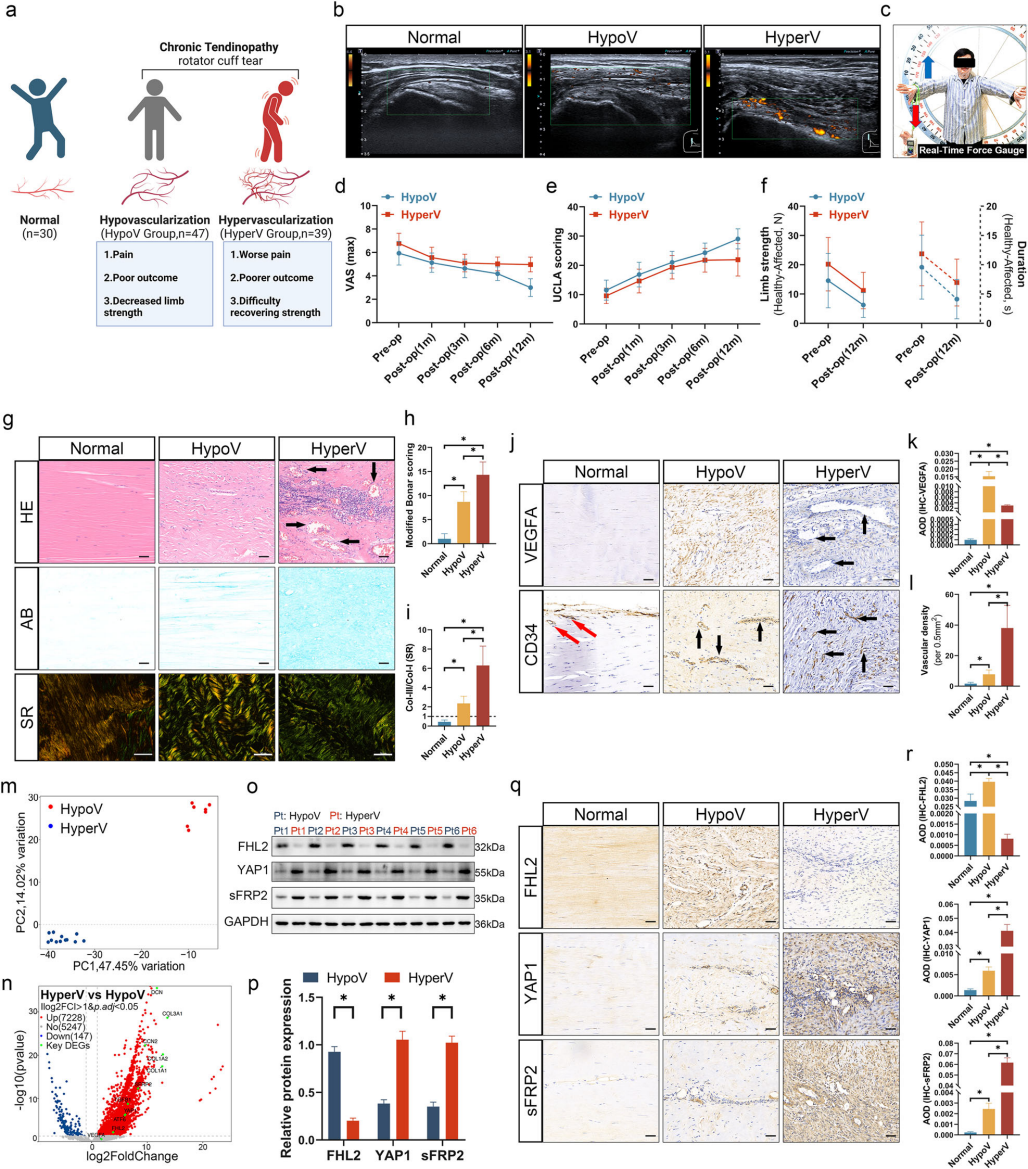
The clinical outcome and key differentially expressed molecules in chronic rotator cuff tendinopathy. **a** A schematic diagram of group allocation, including the normal (normal tendon, Adler blood flow grading 0–I), HypoV (chronic tendinopathy, Adler blood flow grading 0–I) and HyperV groups (chronic tendinopathy, Adler blood flow grading II–III). **b** Preoperative rotator cuff power Doppler imaging in the normal (Adler grading 0–I), HypoV (Adler grading 0–I) and HyperV (Adler grading II–III) groups. The yellow-colored area indicates the presence of slow blood flow, with the intensity of the color representing the signal power. The scale on the side shows the signal power range. **c** An illustration of upper limb resistance strength measurement. Patients maximally resisted the examiner’s downward pull while maintaining position. The maximum resistance force and duration were recorded (Supplementary Data File 1.1.1). **d** Preoperative and postoperative (1, 3, 6 and 12 months) VAS in the HypoV and HyperV groups. **e** The modified UCLA shoulder function scores at each follow-up time point in the HypoV and HyperV groups. **f** A comparison of maximum resistance force difference (in Newtons, left axis) and resistance duration difference (in seconds, right axis) between unaffected and affected sides preoperatively and at 12 months postoperatively in the HypoV and HyperV groups. **g** Representative hematoxylin–eosin staining (HE), Alcian Blue staining (AB) and SR images in the normal, HypoV and HyperV groups. The black arrows indicate extensive angiogenesis (scale bar, 50 μm). **h** Modified Bonar scoring. **i** The area ratio of type III collagen (green thin fibers) to type I collagen (orange thick fibers) in SR staining. **j** IHC staining of VEGFA and CD34 (scale bar, 50 μm). The red arrows indicate vascular accumulation at the tendon sheath boundaries; the black arrows indicate vascular accumulation within the tendon (intratendinous). **k** The average optical density (AOD) for VEGFA in IHC staining. **l** A statistical graph for vascular density (per 0.5 mm^2^, CD34 IHC). **m** A principal component analysis in bulk RNA-seq. **n** A volcano plot of DEGs in bulk RNA-seq. **o** A WB for the protein expression of FHL2, YAP1 and sFRP2 was conducted on six samples from the HyperV and HypoV groups. **p** A statistical chart for relative protein expression in WB. **q** IHC staining of FHL2, YAP1 and sFRP2 (scale bar, 50 μm). **r** The AOD for FHL2, YAP1 and sFRP2 in IHC staining. *P < 0.05.

This study included 30 patients in the normal group (median age ± standard error of 33 ± 0.69 years), 47 in the HypoV group (median age ± standard error of 58 ± 0.73 years) and 39 in the HyperV group (median age ± standard error of 59 ± 0.74 years) (Fig. 1a). Power Doppler imaging revealed significantly stronger blood flow signals in the HyperV group compared with the normal and HypoV groups (Fig. 1b). Supraspinatus tendon injuries were often accompanied by a positive Jobe’s test. Therefore, the maximum resistance force difference and resistance time difference between the healthy and affected upper limbs were measured during this specific physical examination (Fig. 1c).

The VAS scores reflect the level of shoulder pain during patients’ daily activities, with higher values suggesting more severe pain (0–10 scale). The VAS scores in the HyperV and HypoV groups showed an overall downward trend after the surgery (Fig. 1d and Table 1), with VAS scores in the HyperV group consistently higher than those in the HypoV group (*P* < 0.05). In the HyperV group, VAS scores plateaued after three months, with no significant differences among the scores at 3, 6 and 12 months postoperatively (*P* > 0.05). By contrast, the HypoV group exhibited a continuous decline in VAS scores, with the 12-month postoperative VAS significantly lower than the preoperative value (mean difference of 2.936, *P* < 0.05), exceeding the minimal clinically important difference (MCID^VAS^ of 2.4)^39^. This suggests that compared with the HypoV group, patients in the HyperV group may experience more prolonged and severe pain.

**Table 1:** Simple effect analysis of pairwise comparisons between group and time in the VAS.

Subjects			Mean difference	*P*	95% Confidence interval for difference	
Group	Time (*I*)	Time (*J*)	(*I* – *J*)		Lower bound	Upper bound
HypoV	Pre-op	Post-op (1 m)	0.809*	0.000	0.409	1.208
		Post-op (3 m)	1.298*	0.000	0.902	1.694
		Post-op (6 m)	1.745*	0.000	1.328	2.161
		Post-op (12 m)	2.936*	0.000	2.431	3.441
	Post-op (1 m)	Post-op (3 m)	0.489*	0.001	0.142	0.837
		Post-op (6 m)	0.936*	0.000	0.540	1.332
		Post-op (12 m)	2.128*	0.000	1.710	2.545
	Post-op (3 m)	Post-op (6 m)	0.447*	0.000	0.170	0.724
		Post-op (12 m)	1.638*	0.000	1.220	2.057
	Post-op (6 m)	Post-op (12 m)	1.191*	0.000	0.863	1.520
HyperV	Pre-op	Post-op (1 m)	1.205*	0.000	0.767	1.643
		Post-op (3 m)	1.667*	0.000	1.232	2.101
		Post-op (6 m)	1.744*	0.000	1.286	2.201
		Post-op (12 m)	1.795*	0.000	1.240	2.349
	Post-op (1 m)	Post-op (3 m)	0.462*	0.008	0.080	0.843
		Post-op (6 m)	0.538*	0.006	0.104	0.973
		Post-op (12 m)	0.590*	0.004	0.132	1.048
	Post-op (3 m)	Post-op (6 m)	0.077	1.000	−0.227	0.381
		Post-op (12 m)	0.128	1.000	−0.331	0.588
	Post-op (6 m)	Post-op (12 m)	0.051	1.000	−0.309	0.412
**Time**	**Group (*I*)**	**Group (*J*)**				
Pre-op	HypoV	HyperV	−0.833*	0.000	−1.242	−0.424
Post-op (1 m)	HypoV	HyperV	−0.436*	0.020	−0.803	−0.070
Post-op (3 m)	HypoV	HyperV	−0.464*	0.007	−0.798	−0.130
Post-op (6 m)	HypoV	HyperV	−0.834*	0.000	−1.084	−0.584
Post-op (12 m)	HypoV	HyperV	−1.974*	0.000	−2.275	−1.674

*The mean difference is significant at the 0.05 level. m, months; pre-op, preoperative; post-op, postoperative.

The UCLA score is an integrated assessment tool reflecting shoulder pain, function and satisfaction^30^. Higher scores indicate better postoperative recovery (maximum score: 35 points; excellent: 34–35, good: 29–33, fair or poor: <29). Postoperative shoulder joint function in both the HyperV and HypoV groups significantly improved, with an overall upward trend in UCLA scores (Fig. 1e and Table 2; 6-months versus 12-months in the HyperV group, *P* > 0.05). The differences in UCLA scores between 12-months postoperative and preoperative for both groups (HyperV: 12.333 points; HypoV: 17.511 points) were markedly higher than the MCID^UCLA^ (3.5 points)^40^. Although UCLA scores in the HypoV group were higher than those in the HyperV group (*P* < 0.05), the score difference between the two groups (HypoV − HyperV: 7.115) exceeded the MCID^UCLA^ only 12 months after the surgery. This indicates that shoulder joint function recovery was better in the HypoV group than in the HyperV group, with more pronounced long-term differences.

**Table 2:** Simple effect analysis of pairwise comparisons between group and time in the UCLA scoring.

Subjects			Mean difference	*P*	95% Confidence interval for difference	
Group	Time (*I*)	Time *(J)*	(*I* – *J*)		Lower bound	Upper bound
HypoV	Pre-op	Post-op (1 m)	−5.319*	0.000	−6.728	−3.911
		Post-op (3 m)	−9.511*	0.000	−10.978	−8.043
		Post-op (6 m)	−12.766*	0.000	−14.289	−11.243
		Post-op (12 m)	−17.511*	0.000	−19.494	−15.527
	Post-op (1 m)	Post-op (3 m)	−4.191*	0.000	−5.283	−3.100
		Post-op (6 m)	−7.447*	0.000	−9.029	−5.864
		Post-op (12 m)	−12.191*	0.000	−14.221	−10.162
	Post-op (3 m)	Post-op (6 m)	−3.255*	0.000	−4.593	−1.917
		Post-op (12 m)	−8.000*	0.000	−9.914	−6.086
	Post-op (6 m)	Post-op (12 m)	−4.745*	0.000	−6.283	−3.206
HyperV	Pre-op	Post-op (1 m)	−5.051*	0.000	−6.597	−3.505
		Post-op (3 m)	−9.744*	0.000	−11.355	−8.132
		Post-op (6 m)	−12.154*	0.000	−13.826	−10.482
		Post-op(12 m)	−12.333*	0.000	−14.511	−10.156
	Post-op (1 m)	Post-op(3 m)	−4.692*	0.000	−5.890	−3.494
		Post-op(6 m)	−7.103*	0.000	−8.840	−5.366
		Post-op(12 m)	−7.282*	0.000	−9.510	−5.054
	Post-op (3 m)	Post-op(6 m)	−2.410*	0.000	−3.879	−0.941
		Post-op(12 m)	−2.590*	0.006	−4.691	−0.489
	Post-op (6 m)	Post-op(12 m)	−0.179	1.000	−1.869	1.510
Time	Group (*I*)	Group (*J*)				
Pre-op	HypoV	HyperV	1.938*	0.005	0.609	3.266
Post-op (1 m)	HypoV	HyperV	2.206*	0.016	0.424	3.988
Post-op (3 m)	HypoV	HyperV	1.705*	0.044	0.046	3.364
Post-op (6 m)	HypoV	HyperV	2.550*	0.002	0.979	4.121
Post-op (12 m)	HypoV	HyperV	7.115*	0.000	5.169	9.061

*The mean difference is significant at the 0.05 level. m, months; pre-op, preoperative; post-op, postoperative.

Similarly, compared with preoperative values, the 12-month postoperative differences in maximum resistance force and resistance time between the healthy and affected upper limbs significantly decreased in both the HyperV and HypoV groups (*P* < 0.05) (Fig. 1f), suggesting that both groups experienced improvements in the strength and endurance of the affected upper limb at 12 months postoperatively. However, preoperatively or at 12 months postoperatively, the HyperV group showed larger differences in maximum resistance force (preoperative: *P* = 0.005; postoperative: *P* = 0.000) and resistance time (preoperative: *P* = 0.027; postoperative: *P* = 0.001) compared with the HypoV group, suggesting that muscle strength and endurance recovery of the affected shoulder joint in the HyperV group were inferior to those in the HypoV group.

Compared with the normal group, tendon tissue in the HypoV and HyperV groups displayed disorganized and ruptured fiber alignment, in addition to the loss of typical fiber bundle structures and extensive inflammatory cell infiltration (Fig. 1g). These pathological changes were more severe in the HyperV group, which also exhibited significantly increased abundance of blood vessels (HE staining). The HyperV group also showed a marked increase in acidic mucopolysaccharides (AB staining). Moreover, SR staining revealed an increased proportion of type III collagen fibers (Col-III, green; type I collagen appears yellow, Col-I) in the HyperV group, with the disappearance of fiber bundles and a sparse, fragmented state of broken fibers. Based on the modified Bonar scoring system^31^, tendon pathology is assessed through five histological dimensions: cell morphology, collagen arrangement, cellularity, vascularity and ground substance. Higher scores indicate more severe tendon degeneration (maximum score: 20 points). Modified Bonar scores in the HyperV group were significantly higher than those in the HypoV and normal groups (*P* < 0.05) (Fig. 1h). Furthermore, both the HypoV and HyperV groups exhibited a reversal pattern in collagen fiber types (Col-III/Col-I >1), with the proportion of type III collagen fibers in the HyperV group (Col-III/Col-I = 6.30) being significantly higher than that in the HypoV group (Col-III/Col-I = 2.36) (*P* < 0.05) (Fig. 1i).

Significantly increased expression levels of IL-1β were observed in the HypoV and HyperV groups compared with the normal group (Supplementary Fig. 1a, b), with the HyperV group showing higher levels than the HypoV group (*P* < 0.05). However, no significant differences were observed among the three groups regarding IL-6 expression levels (*P* > 0.05). Besides, only the HyperV group exhibited slightly higher levels of TNF-α expression compared with the normal group, and no significant differences were observed between the HypoV and normal groups (*P* > 0.05). These results suggest that IL-1β may be a key inflammatory mediator in the pathogenesis of tendinopathy. Moreover, the proportion of *TGF-β1*-positive cells, an important factor in tissue remodeling^41^, was significantly elevated in both the HypoV and HyperV groups (Supplementary Fig. 1c, d). The HyperV group also exhibited a higher proportion of apoptotic cells (Supplementary Fig. 1e, f).

Vascular density in the HyperV group was significantly higher than that in the normal and HypoV groups (Fig. 1j–l), with vessels predominantly concentrated between collagen fibers (intratendinous). By contrast, blood vessels in the normal group were primarily located along the boundary of the tendon sheath. Interestingly, VEGFA expression was lower in the HyperV group than in the HypoV group (*P* < 0.05), suggesting that VEGFA may not be the key signaling factor driving excessive vascular proliferation in the HyperV group.

In summary, patients with hypervascularized tendinopathy (HyperV group) experienced suboptimal postoperative improvements in pain, functional activity, and upper limb strength. Their tendon tissues exhibited structural disorganization, severe inflammation, increased apoptosis and increased vascular density, suggesting more severe pathological remodeling.

### Differential expression of FHL2/YAP1/sFRP2 is associated with angiogenesis and tissue remodeling

Bulk RNA-seq was conducted on tendon tissues from the HyperV and HypoV groups. A principal component analysis revealed significant differences between the HyperV and HypoV groups, with high similarity among samples in each group (Fig. 1m). KEGG and GO analyses (Supplementary Fig. 1g, h) revealed that the upregulated DEGs (UP-DEGs, HyperV versus HypoV) were mainly enriched in pathways associated with tissue remodeling, cell adhesion, angiogenesis, apoptosis, inflammation and oxidative stress. These pathways included focal adhesion, TGF-β signaling pathway, Wnt signaling pathway, regulation of apoptosis, regulation of angiogenesis and the Hippo signaling pathway. Interestingly, the VEGF signaling pathway was not identified as a key differentially enriched pathway. The volcano plot (Fig. 1n) indicated 7228 upregulated DEGs and 147 downregulated DEGs in the HyperV group compared with the HypoV group. Notably, the transcription levels of genes associated with matrix remodeling, such as *DCN*, *COL3A1*, *COL1A1*, *COL1A2*, *CCN2* and *TGFB1*, were significantly enhanced. Moreover, the transcription levels of *FHL2*, *YAP1*, *sFRP2* and *ATF6* were increased. Wixler et al.^42^ reported that *FHL2* expression in the inflammatory phase of wound repair helps balance inflammation, thereby promoting physiological tissue repair and remodeling. *YAP1*, *sFRP2* and *ATF6* have been implicated in angiogenesis. Studies have suggested that *sFRP2* acts as a critical factor driving pathological angiogenesis in melanoma independent of VEGF^43–45^. Protein interaction analysis also identified potential interactions between *FHL2*, *YAP1*, *TGF-β1* and *ATF6*, suggesting that *FHL2* may serve as an upstream signal, while *YAP1* may act as a key intermediary factor (Supplementary Fig. 1i). GSEA ridge plots demonstrated that the upregulated DEGs were primarily enriched in pathways related to angiogenesis and collagen remodeling (Supplementary Fig. 1j). Furthermore, GSEA line plots revealed that several genes, such as *YAP1* and *sFRP2*, were closely associated with pathways involved in angiogenesis and extracellular matrix remodeling (Supplementary Fig. 1k, l).

The results of WB (Fig. 1o, p) and IHC (Fig. 1q, r) showed that the protein levels of YAP1 and sFRP2 were significantly increased in the HyperV group compared with the normal and HypoV groups (*P* < 0.05). On the contrary, the protein levels of FHL2 were markedly decreased in the HyperV group, despite being identified as an upregulated DEG in RNA-seq. Subsequent multiplex IF staining (Supplementary Fig. 2) revealed that FHL2, YAP1 and sFRP2 were primarily expressed in tenocytes (marked by *TnC*), and partial sFRP2 signals were also detected in endothelial cells (marked by *vWF*). The proportion of *FHL2*^*+*^*TnC*^*+*^ tenocytes was significantly higher in the HypoV group, whereas its proportion in the HyperV group was lower than that in the normal group (Supplementary Fig. 2a). Conversely, the proportions of *YAP1*^*+*^*TnC*^*+*^ tenocytes and *sFRP2*^*+*^*TnC*^*+*^*vWF*^*−*^ tenocytes were significantly higher in the HyperV group compared with the HypoV and normal groups (Supplementary Fig. 2b–d). These findings suggest that the differential expression of FHL2, YAP1 and sFRP2 in tenocytes may be associated with angiogenesis and tissue remodeling.

### Pathological remodeling of tendons was accompanied by downregulation of FHL2 and upregulation of YAP1/sFRP2 expression

**Fig. 2:**
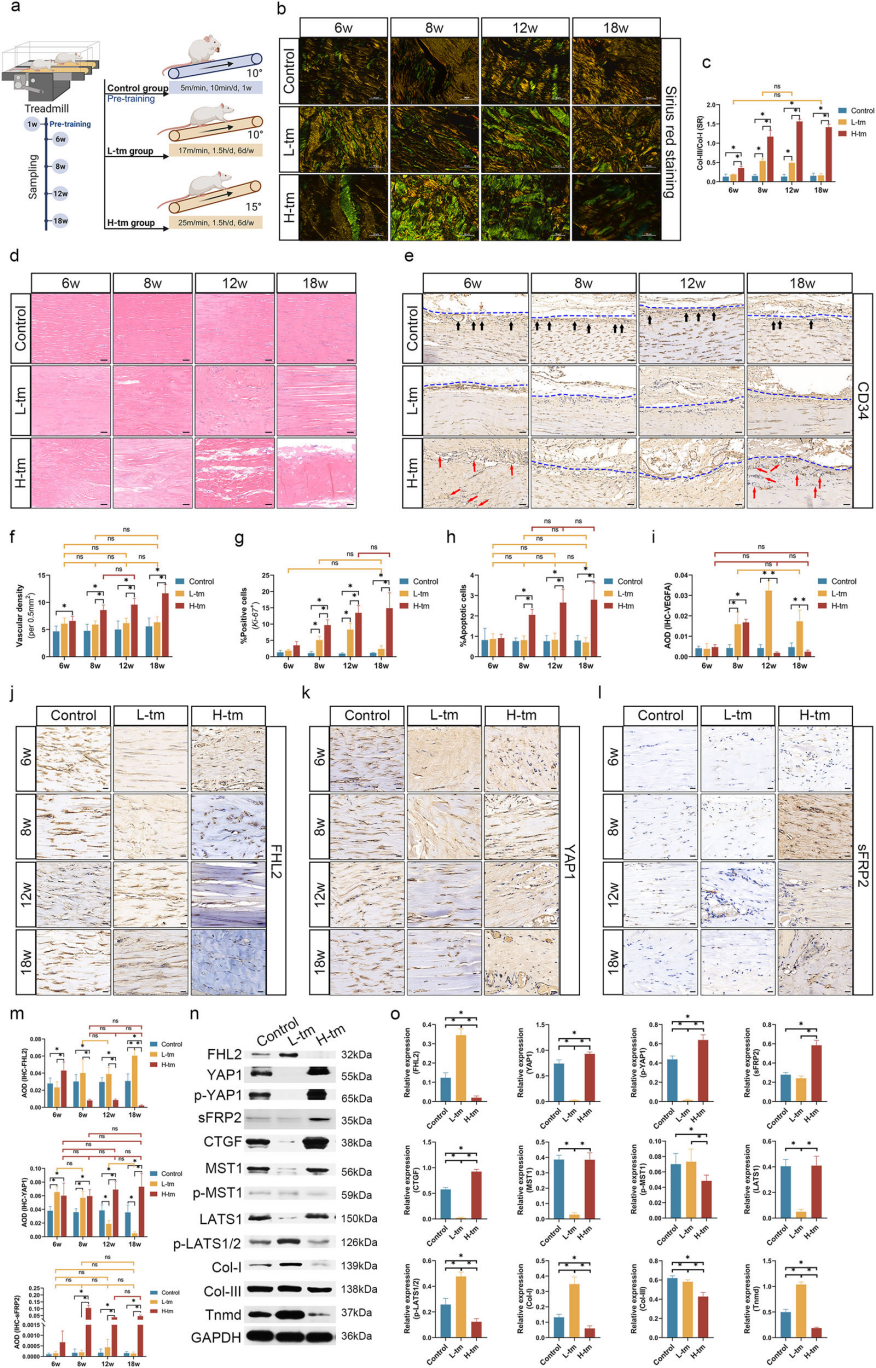
Analysis of exercise-induced rat tendon injury model. **a** Treadmill exercise protocol for rats (Supplementary Data File 1.2.2). **b** SR staining, with type III collagen (Col-III) in green and Col-I in yellow (scale bar, 50 μm). **c** Statistical chart of Col-III/Col-I area ratios in SR staining. **d** Hematoxylin–eosin (HE) staining (scale bar, 50 μm). **e** IHC staining for CD34, with the black arrows indicating blood vessels concentrated at the tendon sheath boundary, red arrows indicating blood vessels concentrated within the tendon (intratendinous) and blue dashed lines marking tendon edges (scale bar, 50 μm). **f** Vascular density in each group (CD34 IHC, per 0.5 mm^2^). **g** The percentages of *Ki-67*^*+*^ proliferative cells (Supplementary Fig. 3a). **h** The percentages of apoptotic cells (TUNEL) (Supplementary Fig. 3b). **i** The average optical density (AOD) for VEGFA in IHC staining (Supplementary Fig. 3c). **j** IHC staining for FHL2 (scale bar, 20 μm). **k** IHC staining for YAP1 (scale bar, 20 μm). **l** IHC staining for sFRP2 (scale bar, 20 μm). **m** AOD for the IHC staining of FHL2, YAP1 and sFRP2. **n** WB of key proteins. **o** A statistical chart for relative protein expression levels in WB. *P < 0.05, ns: *P* > 0.05.

Rat treadmill exercise models were used to induce tendon injury under low-intensity (L-tm) and high-intensity (H-tm) conditions and closely simulate the physiological and pathological progression of tendon degeneration and injury. In addition, we explored the correlations between tendon remodeling, angiogenesis and the expression of FHL2/YAP1/sFRP2 under varying intensities of exercise (Fig. 2a). SR staining (Fig. 2b, c) revealed a time-dependent increase in the Col-III/Col-I ratio in the H-tm group, with a significant reversal of collagen fiber types (Col-III/Col-I >1) after 8 weeks. Collagen fiber bundle structures had disappeared by 12 and 18 weeks, and the fibers were separated and fragmented, presenting as sparse and scattered fine fibrils. By contrast, the L-tm group exhibited a moderate increase in the Col-III/Col-I ratio at 8 and 12 weeks, which remained significantly lower than that of the H-tm group (*P* < 0.05). Compared with the control group, the L-tm group exhibited no significant difference by 18 weeks (*P* > 0.05). Hematoxylin–eosin staining (Fig. 2d) revealed pathological changes, including partial fiber rupture, structural disorganization and collagen remodeling, beginning at 6 and 8 weeks in both the L-tm and H-tm groups. By 12 and 18 weeks, pathological changes were further worsened in the H-tm group, with the disappearance of bundled fiber structures. On the contrary, the L-tm group showed some degrees of recovery toward organized physiological structures. Therefore, the L-tm group exhibited adaptive physiological remodeling of the tendons, whereas the H-tm group appeared to progress into a state of chronic tendinopathy with continued pathological worsening.

CD34 IHC (Fig. 2e) revealed that blood vessels in the control, L-tm and H-tm groups were primarily concentrated along the tendon sheath boundary. However, in the H-tm group, some blood vessels extended into the tendon interior, infiltrating collagen fibers. Vascular density in the H-tm group was slightly higher than that in the L-tm and control groups (L-tm versus control, *P* > 0.05) and exhibited a time-dependent increase (*P* < 0.05) (Fig. 2f). Ki-67 IF staining (Fig. 2g and Supplementary Fig. 3a) revealed that the proportion of proliferating cells (%*Ki-67*^*+*^ cells) in the H-tm group gradually increased over time (*P* < 0.05, versus control and L-tm), and proliferating cells were predominantly localized around blood vessels. At 18 weeks, the proportion of proliferating cells in the L-tm group showed no significant difference from that in the control group (*P* > 0.05). TUNEL staining (Fig. 2h and Supplementary Fig. 3b) also demonstrated that the percentage of apoptotic cells in the H-tm group increased over time, which was significantly higher than that in the control and L-tm groups (*P* < 0.05; but L-tm versus control, *P* > 0.05). These findings suggest that the H-tm group may be undergoing an active remodeling phase characterized by both cell proliferation and apoptosis.

Interestingly, IHC (Fig. 2i and Supplementary Fig. 3c) showed that although vascular abundance did not significantly increase in the L-tm group, VEGFA expression was markedly enhanced at 8, 12 and 18 weeks, significantly exceeding that in the control group (*P* < 0.05). Despite showing a significant increase in vascular density, the H-tm group exhibited a peak in VEGFA expression at 8 weeks (higher than the control group, *P* < 0.05), followed by relatively low levels at other time points (versus control, *P* > 0.05). This lack of a positive correlation between VEGFA expression and vascular abundance is consistent with findings in the clinical study and suggests that VEGFA may not be the primary proangiogenic factor increasing the abundance of blood vessels in the H-tm group.

IHC revealed that in the H-tm group, FHL2 showed a transient peak at 6 weeks, followed by a sharp decline at 8, 12 and 18 weeks, significantly lower than that in the control and L-tm groups (*P* < 0.05) (Fig. 2j). By contrast, FHL2 expression in the L-tm group showed a time-dependent increase. YAP1 expression (Fig. 2k) in the H-tm group was consistently high, whereas the L-tm group showed a time-dependent decrease in YAP1 expression. By 18 weeks, YAP1 expression in the L-tm group was significantly lower than that in the control group. Similarly, concerning sFRP2 expression (Fig. 2l), the H-tm group had significantly higher levels than the control and L-tm groups (*P* < 0.05; but control versus L-tm, *P* > 0.05). Figure 2m illustrates the statistical differences among FHL2, YAP1 and sFRP2 across the different groups.

WB for the expression of key proteins at 18 weeks in the H-tm, L-tm and control groups (Fig. 2n, o) also revealed that the H-tm group showed reduced FHL2 levels and increased YAP1/sFRP2 levels. These changes were accompanied by increased expression of connective tissue growth factor (CTGF) and Col-III and decreased levels of Col-I and tenomodulin (Tnmd), suggesting pathological tendon remodeling^46,47^. Upon phosphorylation, mammalian sterile 20-like kinase 1 (MST1) and large tumor suppressor kinase 1 (LATS1), as core kinases of the Hippo signaling pathway, induce YAP1 phosphorylation (p-YAP1), thereby downregulating its transcriptional activity in the nucleus^48^. Phospho-MST1 and phospho-LATS1/2 levels were significantly decreased in the H-tm group (*P* < 0.05), facilitating the nuclear translocation of YAP1. However, p-YAP1 levels in the H-tm group were not lower than those in the L-tm group (*P* < 0.05), probably due to higher YAP1 expression levels in the H-tm group. These findings suggest that in the rat model of exercise-induced tendinopathy, reduced FHL2 and elevated YAP1/sFRP2 levels were closely associated with angiogenesis and temporal progression of tissue remodeling.

### Downregulation of FHL2 and upregulation of YAP1/sFRP2 may be key factors in the temporal remodeling of vascular patterns in tendinopathy

**Fig. 3:**
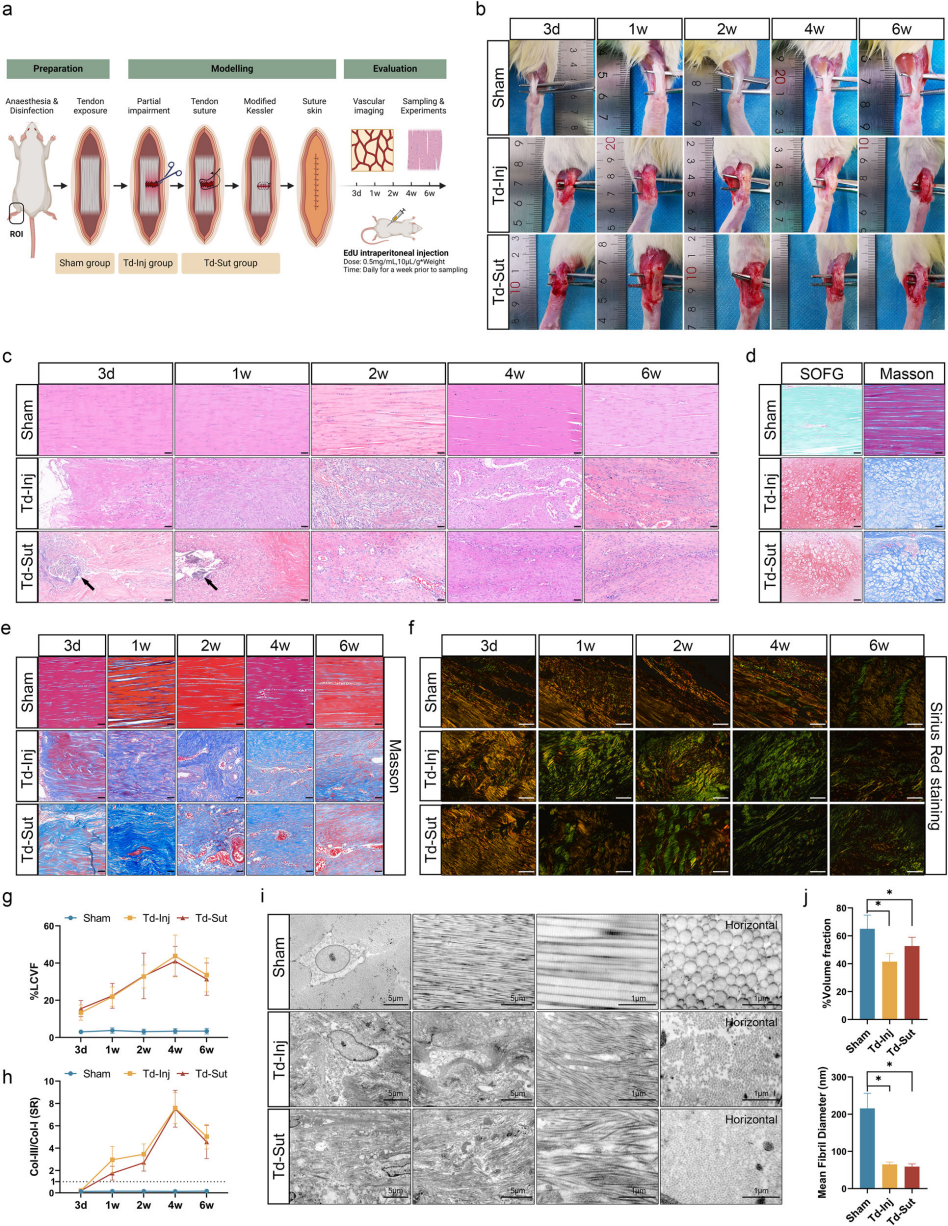
The histological features of the trauma-induced rat tendon injury model. **a** A schematic diagram of tendon injury/suture modeling, including the sham group, tendon injury (Td-Inj) group and tendon injury with suture (Td-Sut) group, with daily EdU injection to label proliferating cells 1 week before sampling (Supplementary Data File 1.2.3). **b** Gross specimens of the Achilles tendon from the sham, Td-Inj and Td-Sut groups. **c** Hematoxylin–eosin (HE) staining, with black arrows indicating sutures (scale bar, 50 μm). **d** Safranin O-Fast green (SOFG) and Masson stainings, showing cartilage formation in parts of the tendon tissues of Td-Inj and Td-Sut groups at 4–6 weeks (scale bar, 50 μm). **e** Masson staining indicating fiber compactness (scale bar, 50 μm). **f** SR staining showing collagen types, with type III collagen (Col-III) in green (fine fibers) and type I collagen (Col-I) in orange (coarse fibers) (scale bar, 100 μm). **g** Loose collagen volume fraction from Masson staining (%LCVF = blue-stained area/total collagen area); comparisons between the Td-Inj group and the Td-Sut group showed *P* > 0.05 at all time points, whereas comparisons between the sham group and the Td-Inj or Td-Sut groups showed *P* < 0.05. **h** A statistical chart of Col-III/Col-I ratios in SR staining, with values greater than 1 suggesting collagen-type inversion. **i** TEM of tendon tissues, with the first three columns showing longitudinal sections and the fourth column showing cross sections. **j** Statistical charts of collagen volume fraction (%volume fraction = collagen area/total area) and mean fibril diameter (Feret’s diameter, nanometers) in TEM cross-sections of mid-tendon. *P < 0.05.

We established a rat trauma-induced tendon injury model (Td-Inj), a tendon suture model (Td-Sut) and a sham-operated control (Sham) through surgical methods to simulate clinical tendon tears and investigate the temporal pathological characteristics from acute tendon injury to chronic tendinopathy (Fig. 3a). Gross specimens of tendons (Fig. 3b) showed clearly severed tendon ends in the Td-Inj group 3 days after modeling, with well-sutured tendon ends in the Td-Sut group. Tendon tissues exhibited significant swelling from 1 to 4 weeks, with the Td-Inj group revealing more severe swelling compared with the Td-Sut group. By 6 weeks, sutures were still visible in the Td-Sut group, but both the Td-Inj and Td-Sut groups presented tissue swelling and hemorrhagic tendencies.

HE staining (Fig. 3c) revealed that collagen bundles in the sham group were clear, orderly and compact, displaying features of avascularity and sparse cellularity. In the Td-Inj and Td-Sut groups, tendon injury sites and sutures were visible at 3 days, with loosened fiber structures. By 1 week, both groups exhibited hypercellularity and disorganized collagen structures. No obvious bundled fiber structures were observed in the Td-Inj and Td-Sut groups between 2 and 6 weeks. Further disorganization and irregularity of tissue structures were accompanied by a significant increase in intrafibrillar vascularization. Safranin O-Fast green staining and Masson staining (Fig. 3d) indicated that partial tendon tissues in the Td-Inj and Td-Sut groups showed tendon calcification and cartilage formation between 4 and 6 weeks.

In Masson staining (Fig. 3e), the sham group exhibited dense fiber structures (stained red), whereas the Td-Inj and Td-Sut groups showed loose and disorganized fiber structures (increased blue-stained areas) and pronounced vascular proliferation between weeks 2 and 6. SR staining (Fig. 3f) indicated the disappearance of bundled fiber structures in the Td-Inj and Td-Sut groups from 1 to 6 weeks. Between 4 and 6 weeks, both groups exhibited significant fiber separation and sparse, scattered fine fibrils, suggesting progression to a severe pathological state of chronic tendinopathy. Quantitative analysis revealed a significant increase in the loose collagen volume fraction (%LCVF) (Fig. 3g) in the Td-Inj and Td-Sut groups compared with the sham group (*P* < 0.05), with a slight decrease at 6 weeks. However, no significant differences were observed between the Td-Inj and Td-Sut groups (*P* > 0.05), suggesting that even at 6 weeks, the fiber structures in both groups did not return to a normally dense and orderly state. Furthermore, the proportion of Col-III in the Td-Inj and Td-Sut groups significantly increased from week 1 to week 6, leading to collagen fiber type reversal (Col-III/Col-I >1) (Fig. 3h).

Transmission electron microscopy (TEM) (Fig. 3i) of longitudinal tendon sections revealed loose collagen fibers in the Td-Inj and Td-Sut groups, with most fibers fractured and arranged irregularly. Compared with the sham group, cross-sectional images showed thinner and sparser collagen fibers in these groups. Quantitative analysis of collagen fiber volume fraction and Feret’s diameter (Fig. 3j) revealed a significant decrease in collagen fiber volume in the Td-Inj and Td-Sut groups, with mean fiber diameters far less than those of the sham group (Sham: 215.67 nm, Td-Inj: 65.00 nm, Td-Sut: 59.17 nm, *P* < 0.05). In summary, the Td-Inj and Td-Sut groups exhibited severe pathological remodeling, which progressed into the chronic state of tendinopathy by 6 weeks. Tendon suturing did not effectively prevent this pathological progression.

Further assessment of inflammation severity in tendon tissues (Supplementary Fig. 4a–c) revealed distinct expression patterns of key inflammatory mediators. Consistent with clinical findings (Supplementary Fig. 1a), IHC showed significantly higher expression of IL-1β (Supplementary Fig. 4a, g) in the Td-Inj and Td-Sut groups compared with the sham group in all time points (*P* < 0.05), suggesting that IL-1β may serve as a critical inflammatory mediator in tendinopathy. From week 2 to week 6, IL-1β expression in the Td-Inj and Td-Sut groups appeared to plateau, with no significant differences between 2, 4 and 6 weeks (*P* > 0.05). The overall expression of IL-6 was low across groups (Supplementary Fig. 4b, h). The Td-Inj and Td-Sut groups exhibited expression peaks at 1 and 2 weeks, respectively, but no significant differences were observed among the three groups at later time points (*P* > 0.05), suggesting that IL-6 is less likely a major contributor to disease progression. IHC indicated that the Td-Inj group had slightly higher expression of TNF-α (Supplementary Fig. 4c, i) than the sham group at 3 days and 1 week, but no significant differences were observed at subsequent time points (*P* > 0.05). By contrast, compared with the sham group, the Td-Sut group exhibited significantly lower TNF-α expression at 3 days and 1 week. TNF-α expression in the Td-Sut group revealed a time-dependent increase, which was significantly more than that in the sham group by 6 weeks (*P* < 0.05). The opposing trends in TNF-α expression between the Td-Inj and Td-Sut groups may be associated with the presence of sutures in the Td-Sut model.

Concerning common MMPs involved in matrix remodeling (Supplementary Fig. 4d–f, j–l), MMP2, MMP3 and MMP9 in the Td-Inj and Td-Sut groups exhibited early expression peaks. However, by 6 weeks, the expression levels of these MMPs in the Td-Inj and Td-Sut groups were not significantly different from those in the sham group (*P* > 0.05). This finding also suggests that pathological remodeling might have reached a plateau. In addition, IF staining (Supplementary Fig. 4m, n) demonstrated significantly higher average fluorescence intensity of TGF-β1 in the Td-Inj and Td-Sut groups compared with the sham group at all time points. Although the intensity showed a time-dependent decline after 1 week, it remained at relatively high levels. The sustained and high expression of IL-1β and TGF-β1 may play critical roles in pathological tissue remodeling.

Interestingly, TUNEL staining (Supplementary Fig. 5a, b) showed that although the proportion of apoptotic cells was significantly higher in the Td-Inj and Td-Sut groups than in the sham group (*P* < 0.05), apoptotic cells were primarily localized at the tendon sheath boundary (Supplementary Fig. 5c). The fascia at the tendon sheath boundary serves as an important reservoir of tendon stem/progenitor cells (TSPCs)^49^. IF staining for TSPCs (*Tnmd*^*+*^*CD90*^*+*^*CD44*^*+*^ positive cells) (Supplementary Fig. 5d, e) revealed that the proportions of TSPCs in the Td-Inj and Td-Sut groups were significantly higher than those in the sham group at day 3, week 1 and week 2 (*P* < 0.05). However, by 4 and 6 weeks, the proportions of TSPCs in the Td-Inj and Td-Sut groups were not significantly different from those in the sham group (*P* > 0.05). The late decrease in the proportion of TSPCs may be due to their depletion during differentiation into tenocytes or due to the apoptosis of TSPCs. Nevertheless, either depletion or apoptosis of TSPCs can adversely affect tissue regeneration and repair.

**Fig. 4:**
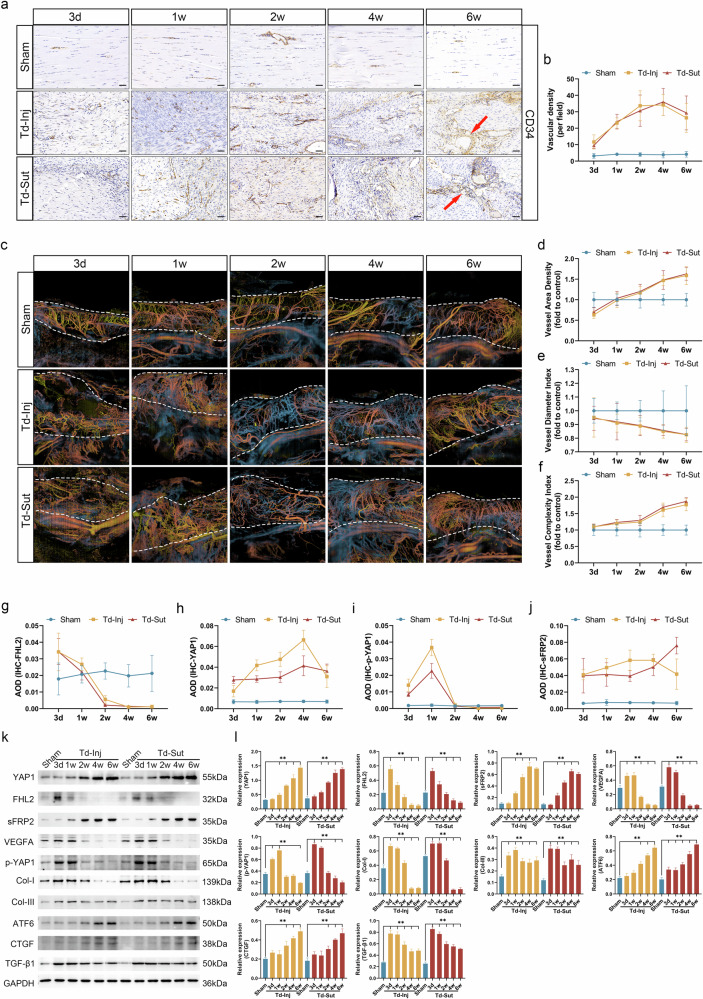
Pathological vascular pattern remodeling in tendons. **a** CD34 IHC staining, with the red arrows indicating clustered vessels (scale bar, 50 μm). **b** Vascular density in each group (per field, CD34 IHC). **c** OCTA showing the spatial distribution, morphology and abundance of vascular remodeling in the Td-Inj and Td-Sut groups, with dashed lines marking region of interest. **d** A statistical chart of VAD. **e** A statistical chart of VDI. **f** A statistical chart of VCI. **g–j** Statistical charts of average optical density (AOD) for FHL2 (**g**), YAP1 (**h**) p-YAP1 (**i**) and sFRP2 (**j**) in IHC staining. **k** WB of key proteins in the sham, Td-Inj and Td-Sut groups. **l** The relative protein expression levels measured using WB (**P < 0.05 versus sham).

The angiogenesis-regression homeostasis is critical for tissue repair after injury^15,21^. We found that VEGFA expression in the Td-Inj and Td-Sut groups significantly increased from day 3 to week 2 (*P* < 0.05) but sharply declined at week 4 and week 6, with no significant differences compared with the sham group (*P* > 0.05) (Supplementary Fig. 5f, g). Compared with the sham group, IHC (Fig. 4a, b) for CD34 showed significantly higher vascular density in the Td-Inj and Td-Sut groups across all time points (*P* < 0.05). Clustered vascular structures began to appear by week 4 to week 6. However, no significant differences in vascular density were observed between week 4 and week 6 in either the Td-Inj group (*P* = 0.251) or the Td-Sut group (*P* = 0.555).

To further analyze the vascular spatial pattern characteristics, including VAD, VDI and VCI, we conducted in vivo vascular imaging using OCTA technology. The Td-Inj and Td-Sut groups exhibited vascular defects in tendons at day 3 and week 1, characterized by poor vascular continuity and tissue hemorrhage (Fig. 4c). From week 2 to week 6, both groups showed increased vascular density, with numerous clustered or aggregated vessels predominantly distributed within the tendons. Analysis of the proportion of vascular area (Fig. 4d) revealed that VAD in the Td-Inj and Td-Sut groups was lower than that in the sham group at day 3, possibly due to vascular damage during modeling. Subsequently, vascular density increased, becoming significantly higher than that of the sham group at week 4 and week 6 (*P* < 0.05). Interestingly, vessel diameter analysis (Fig. 4e) demonstrated that compared with the sham group, VDI in the Td-Inj and Td-Sut groups decreased progressively (*P* < 0.05), suggesting the formation of a large number of blood vessels. Further analysis of vascular complexity (Fig. 4f) indicated that VCI in the Td-Inj and Td-Sut groups continued to increase, with vascular complexity significantly higher than that of the sham group at week 6 (*P* < 0.05). However, there were no significant differences in VAD, VDI or VCI between the Td-Inj and Td-Sut groups (*P* > 0.05). In summary, both the Td-Inj and Td-Sut groups experienced vascular spatial pattern remodeling, characterized by the formation of many spatially complex blood vessels predominantly distributed in the tendons, with no significant regression at week 6.

Subsequently, we analyzed the expression of signaling molecules, such as FHL2, YAP1 and sFRP2 (Fig. 4g–l). IHC (Fig. 4g and Supplementary Fig. 6a) revealed that FHL2 expression in the Td-Inj and Td-Sut groups was slightly higher than that in the sham group at day 3 but declined progressively over time and became significantly lower than that in the sham group (*P* < 0.05). Conversely, at all time points, YAP1 and sFRP2 levels were significantly higher in the Td-Inj and Td-Sut groups compared with the sham group (*P* < 0.05), with p-YAP1 levels showing a slight increase at day 3 and week 1, followed by a sustained decrease (Fig. 4h–j and Supplementary Fig. 6b–d). These findings indicate a negative correlation between downregulation of FHL2 expression and upregulation of YAP1 and sFRP2 expression in the Td-Inj and Td-Sut groups, suggesting that FHL2 may play a critical role in inhibiting YAP1 and sFRP2 expression, the relevant statistics for which are presented in Supplementary Fig. 6e–h. This trend was further validated by WB (Fig. 4k, l). In addition, WB showed that Col-III, CTGF, and TGF-β1 levels were consistently higher in the Td-Inj and Td-Sut groups (versus sham group, *P* < 0.05). On the contrary, Col-I expression transiently increased at day 3 and week 1 and decreased subsequently, becoming significantly lower than that in the sham group at weeks 4 and 6 (*P* < 0.05). These results are consistent with the SR staining trends, indicating collagen remodeling. RNA-seq analysis identified *ATF6* as a significantly upregulated gene in patients with hypervascularized tendinopathy, and its expression was also found to increase progressively in the Td-Inj and Td-Sut groups, suggesting that ATF6 may contribute to sustained angiogenesis^45^. Consistent with clinical findings and in vivo findings, VEGFA expression in the Td-Inj and Td-Sut groups increased at early stages but decreased over time, finally becoming significantly lower than that in the sham group (*P* < 0.05) (Fig. 4k, l). These findings suggest that although VEGFA is a common proangiogenic factor, it may not be a key regulatory factor in the pathological vascular remodeling associated with tendinopathy. By contrast, downregulation of FHL2 expression and upregulation of YAP1/sFRP2 expression are strongly and positively correlated with the temporal pathological remodeling and angiogenesis associated with tendinopathy.

**Fig. 5:**
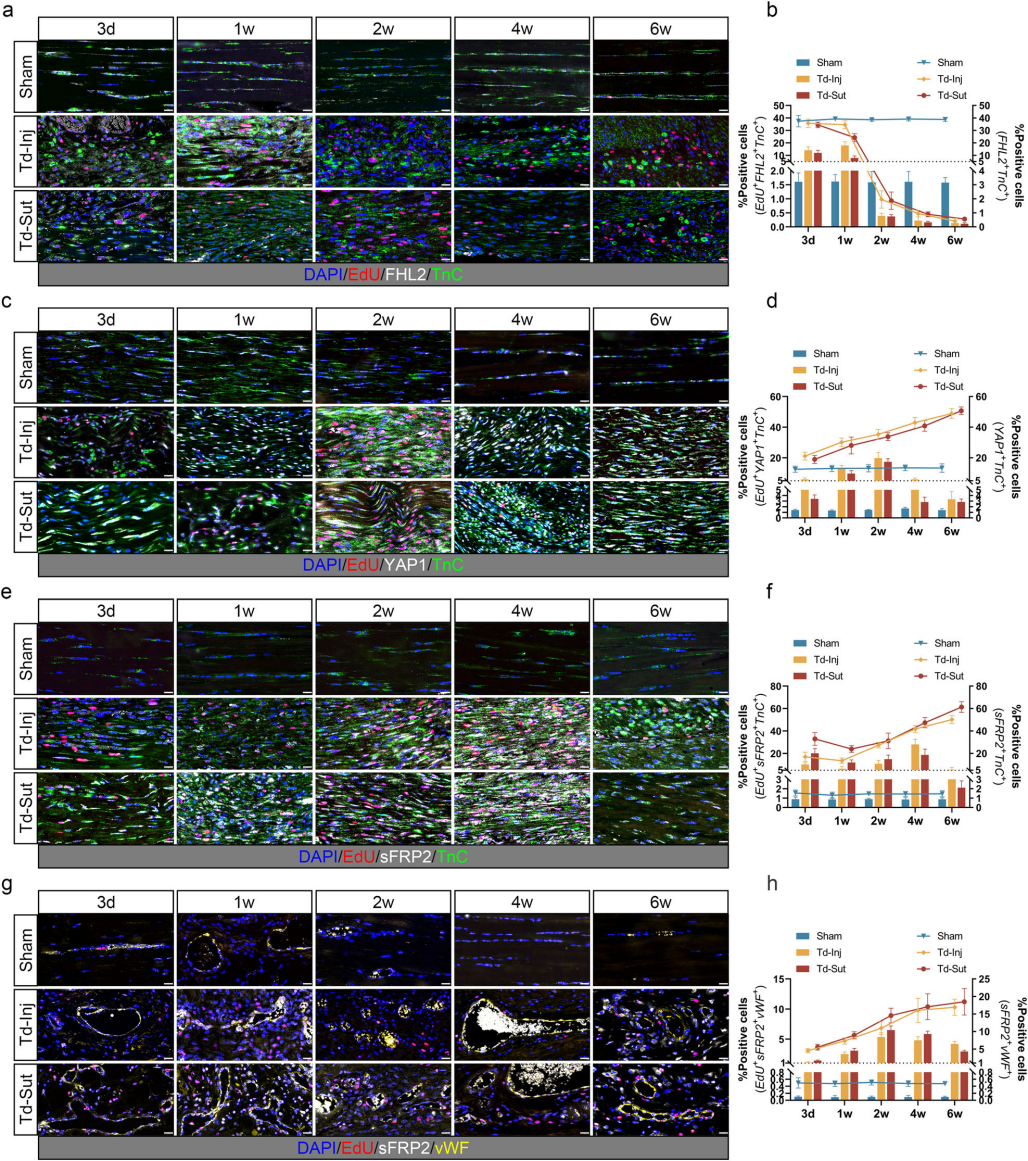
The proportion and proliferation (EdU labeling) of *FHL2*^*+*^, *YAP1*^*+*^ and *sFRP2*^*+*^ cells. **a** Multiplex IF staining of *EdU*^*+*^*FHL2*^*+*^*TnC*^*+*^ tenocytes. **b** The percentage of *EdU*^*+*^*FHL2*^*+*^*TnC*^*+*^ (proliferative, left axis) and *FHL2*^*+*^*TnC*^*+*^ (right axis) cells. **c** Multiplex IF of *EdU*^*+*^*YAP1*^*+*^*TnC*^*+*^ tenocytes. **d** The percentage of *EdU*^*+*^*YAP1*^*+*^*TnC*^*+*^ (proliferative, left axis) and *YAP1*^*+*^*TnC*^*+*^ (right axis) cells. **e** Multiplex IF of *EdU*^*+*^*sFRP2*^*+*^*TnC*^*+*^ tenocytes. **f** The percentage of *EdU*^*+*^*sFRP2*^*+*^*TnC*^*+*^ (proliferative, left axis) and *sFRP2*^*+*^*TnC*^*+*^ (right axis) cells. **g** Multiplex IF of *EdU*^*+*^*sFRP2*^*+*^*vWF*^*+*^ endothelial cells. **h** The percentage of *EdU*^*+*^*sFRP2*^*+*^*vWF*^*+*^ (proliferative, left axis) and *sFRP2*^*+*^*vWF*^*+*^ (right axis) cells. Scale bar, 20 μm.

Using multiplex IF staining, we analyzed the proportions of *FHL2*^*+*^, *YAP1*^*+*^ and *sFRP2*^*+*^ cells and their proliferation status (EdU labeling) (Fig. 5). Both FHL2 and YAP1 were predominantly expressed in tenocytes (*TnC*^*+*^). In the sham group, the proportion of *FHL2*^*+*^*TnC*^*+*^ tenocytes was relatively high, reaching approximately 40%, with proliferative *EdU*^*+*^*FHL2*^*+*^*TnC*^*+*^ tenocytes accounting for only about 1.5–1.7% of all tenocytes (Fig. 5a, b). Although the proportion of proliferative *EdU*^*+*^*FHL2*^*+*^*TnC*^*+*^ tenocytes significantly increased in the Td-Inj and Td-Sut groups in the early stages of tendon injury (from 3 days to 1 week) (approximately 7–18%; versis sham, *P* < 0.05), the overall proportion of *FHL2*^*+*^*TnC*^*+*^ tenocytes began to decrease. Between week 2 and week 6, the proportion of *FHL2*^*+*^*TnC*^*+*^ tenocytes in the Td-Inj and Td-Sut groups was significantly lower than that in the sham group (*P* < 0.05). The reduction in FHL2 may be associated with the destabilization of tenocyte function and pathological tissue remodeling. By contrast, compared with the sham group, the proportion of *YAP1*^*+*^*TnC*^*+*^ tenocytes continuously increased in the Td-Inj and Td-Sut groups (*P* < 0.05), reaching 48.96% and 50.73% at week 6, respectively (Fig. 5c, d). However, the proportion of proliferative *EdU*^*+*^*YAP1*^*+*^*TnC*^*+*^ tenocytes in the Td-Inj and Td-Sut groups peaked at week 2 and then began to decline, showing no significant difference compared with the sham group at week 6 (*P* > 0.05). A decreased proportion of proliferative cells at week 6 may be associated with the gradual cessation of active tissue remodeling, while the persistent increase in YAP1 signaling at this stage may not be protective.

Although sFRP2 was primarily expressed in tenocytes (Fig. 5e, f), sFRP2 signals were also detectable in a subset of endothelial cells (*vWF*^*+*^) (Fig. 5g, h). Compared with the sham group, the proportion of *sFRP2*^*+*^*TnC*^*+*^ cells and *sFRP2*^*+*^*vWF*^*+*^ cells in the Td-Inj and Td-Sut groups exhibited a time-dependent increase (*P* < 0.05). Moreover, at all time points, the proportion of *sFRP2*^*+*^*TnC*^*+*^ cells was consistently higher than that of *sFRP2*^*+*^*vWF*^*+*^ cells across all groups (*P* < 0.05). Similar to *YAP1*^*+*^*TnC*^*+*^ tenocytes, at week 6, the proportions of *sFRP2*^*+*^*TnC*^*+*^ tenocytes in the Td-Inj and Td-Sut groups reached 50.24% and 61.38%, respectively. Moreover, the number of proliferating *EdU*^*+*^*sFRP2*^*+*^*TnC*^*+*^ tenocytes decreased, suggesting a potential positive regulatory relationship between YAP1 and sFRP2.

Furthermore, unlike *EdU*^*+*^*YAP1*^*+*^*TnC*^*+*^ and *EdU*^*+*^*sFRP2*^*+*^*TnC*^*+*^ tenocytes, the proportion of proliferating *EdU*^*+*^*sFRP2*^*+*^*vWF*^*+*^ endothelial cells in the Td-Inj and Td-Sut groups remained significantly higher than that in the sham group (*P* < 0.05), even at the late stage of the disease (week 6). This finding indicates that angiogenesis may be ongoing and partly associated with the persistent presence of sFRP2 signaling. However, it remains unclear whether sFRP2 is expressed by endothelial cells, tenocytes or both of them.

### *YAP1* knockdown reduced sFRP2 expression and alleviated pathological vascular remodeling

**Fig. 6:**
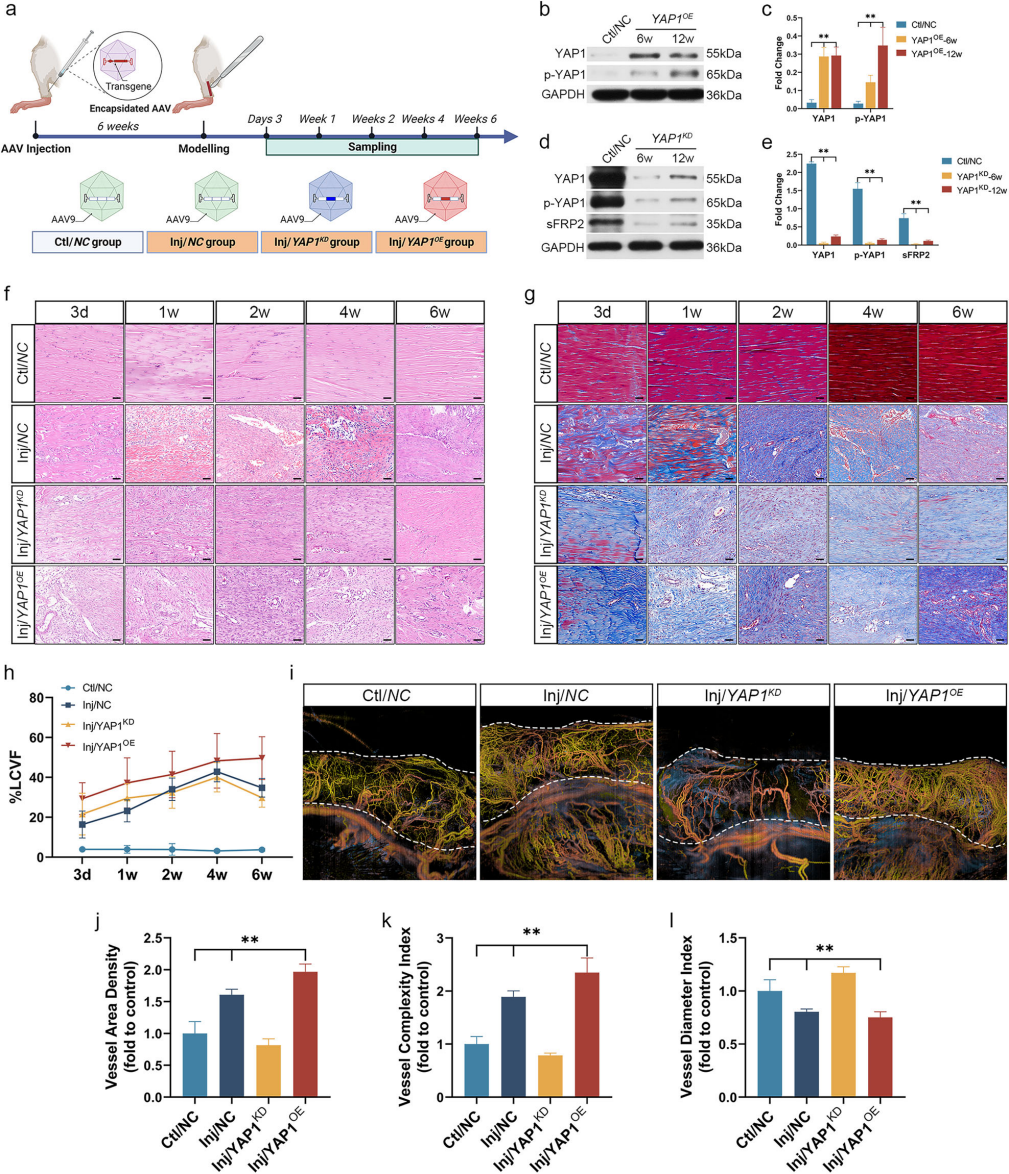
Construction of *YAP1*-knockdown (*YAP1*^*KD*^)/overexpression (*YAP1*^*OE*^) tendon injury models and assessment of vascular patterns. **a** The schematic diagram of model construction. AAV-*NC*, AAV-*YAP1*^*KD*^ and AAV-*YAP1*^*OE*^ transfection was conducted, followed by tendon injury modeling at 6 weeks to establish Ctl/*NC*, Inj/*NC*, Inj/*YAP1*^*KD*^ and Inj/*YAP1*^*OE*^ groups. **b**, **c** Tendon samples were collected 6 and 12 weeks after AAV-*YAP1*^*OE*^ transfection without tendon injury modeling. The reliability of transfection was assessed by WB, with the relevant statistical results displayed in panel **c**. **d**, **e** Tendon samples were collected 6 and 12 weeks after AAV-*YAP1*^*KD*^ transfection without tendon injury modeling. The reliability of transfection was assessed by WB, with the relevant statistical results displayed in panel **e**. **f** Hematoxylin–eosin (HE) staining (scale bar, 50 μm). **g** Masson staining (scale bar, 50 μm). **h** A statistical chart of loose collagen volume fraction (%LCVF) from Masson staining. **i** The OCTA of tendons 6 weeks after modeling, with dashed lines showing the tendon region (region of interest). **j** A statistical chart of VAD. **k** A statistical chart of VCI. **l** A statistical chart of VDI. **P < 0.05 versus Ctl/*NC*.

We constructed *YAP1*-knockdown (*YAP1*^*KD*^) and *YAP1*-overexpression (*YAP1*^*OE*^) models using AAV transfection to investigate the roles of FHL2/YAP1/sFRP2 in vascular remodeling in tendons (Fig. 6a). Tendon samples were collected at week 6 and week 12 after transfection without tendon injury modeling, and YAP1 expression was measured via WB. YAP1 expression significantly increased at both 6 and 12 weeks after AAV-*YAP1*^*OE*^ transfection (*P* < 0.05) (Figs. 6b,c). Conversely, *YAP1* expression significantly decreased at both week 6 and week 12 after AAV-*YAP1*^*KD*^ transfection (*P* < 0.05) (Figs. 6d, e). These results confirm the successful and reliable construction of the *YAP1*^*KD*^ and *YAP1*^*OE*^ models throughout the 12-week experimental period. Subsequently, tendon injury modeling was constructed in combination with surgery.

HE staining (Fig. 6f) revealed that in the Inj/*YAP1*^*KD*^ group, despite disorganized and irregular pathological features from day 3 to week 2, the tissue structure improved from week 4 to week 6 compared with the Inj/*NC* group, with significantly reduced vascular formation. By contrast, the Inj/*YAP1*^*OE*^ group exhibited pronounced pathological features, including disorganized and irregular tissue structure and disappearance of bundle structures, with apparent angiogenesis even at week 6, which was more extensive than that in the Inj/*NC* group. These findings suggest that *YAP1* knockdown may restore tissue structure. Masson staining (Fig. 6g, h) demonstrated that %LCVF in the Inj/*NC*, Inj/*YAP1*^*KD*^ and Inj/*YAP1*^*OE*^ groups was significantly higher than that in the Ctl/*NC* group at all time points (*P* < 0.05). Unfortunately, no significant difference in %LCVF was observed between the Inj/*NC* and Inj/*YAP1*^*KD*^ groups (*P* > 0.05). It appears that *YAP1* knockdown alone is insufficient to return the structural density of tendon fibers to normal levels in the short term.

At 6 weeks after injury, OCTA assessment (Fig. 6i–l) revealed that compared with the Inj/*NC* group, the Inj/*YAP1*^*KD*^ group exhibited significantly decreased VAD and VCI, along with significantly increased VDI (*P* < 0.05; versus Ctl/*NC* group, *P* > 0.05). Conversely, the Inj/*YAP1*^*OE*^ group showed significantly higher VAD and VCI than the Inj/*NC* group (*P* < 0.05). These results suggest that *YAP1* overexpression exacerbated pathological microvascular proliferation, whereas *YAP1* knockdown effectively suppressed this pathological vascular remodeling.

**Fig. 7:**
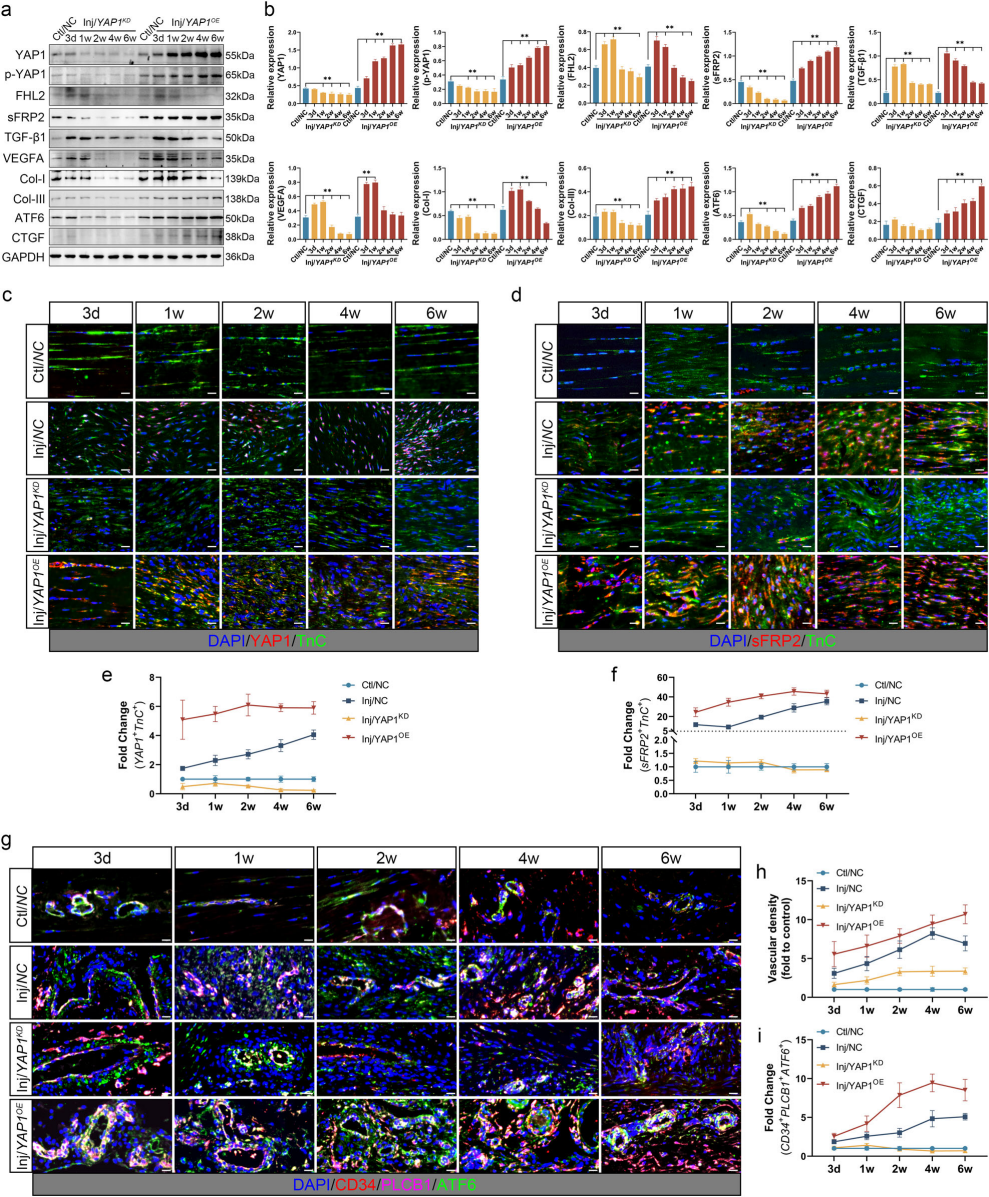
The expression of key signaling proteins (FHL2/YAP1/sFRP2) after *YAP1* knockdown/overexpression. **a**, **b** WB (panel **a**) and statistical charts (panel **b**) for the relative expression of key proteins in Ctl/*NC*, Inj/*YAP1*^*KD*^ and Inj/*YAP1*^*OE*^ groups (**P < 0.05 versus Ctl/*NC*). **c** Multiplex IF staining of *YAP1*^*+*^*TnC*^*+*^ tenocytes. **d** Multiplex IF of *sFRP2*^*+*^*TnC*^*+*^ tenocytes. **e**, **f** The proportion of *YAP1*^*+*^*TnC*^*+*^ and *sFRP2*^*+*^*TnC*^*+*^ cells (fold to Ctl/*NC*). **g** Multiplex IF of *CD34*^*+*^*PLCB1*^*+*^*ATF6*^*+*^ endothelial cells. **h** Vascular density in each group (fold to Ctl/*NC*, CD34). **i** The proportion of *CD34*^*+*^*PLCB1*^*+*^*ATF6*^*+*^ cells (fold to Ctl/*NC*). Scale bar, 20 μm.

WB (Fig. 7a, b) demonstrated that the expression of both YAP1 and sFRP2 was significantly upregulated in the Inj/*YAP1*^*OE*^ group, while significantly downregulated in the Inj/*YAP1*^*KD*^ group (*P* < 0.05), suggesting that YAP1 positively regulated sFRP2 expression. A similar trend was observed for p-YAP1 levels, which may be associated with YAP1 expression. Interestingly, FHL2 expression appeared to be unaffected or mildly affected in both the Inj/*YAP1*^*KD*^ and Inj/*YAP1*^*OE*^ groups, showing an early and transient increase followed by a sustained decrease over time, consistent with the trends observed in Fig. 4k. This finding suggests that FHL2 is less likely to be a downstream regulatory target of YAP1. Similarly, no significant change was observed for TGF-β1 expression (persistently high expression). Moreover, VEGFA expression exhibited an early increase followed by a subsequent decline, with overall expression levels slightly higher in the Inj/*YAP1*^*OE*^ group than in the Inj/*YAP1*^*KD*^ group, reflecting more active angiogenesis in the Inj/*YAP1*^*OE*^ group. Another angiogenesis-related factor, ATF6, showed a positive correlation with YAP1 expression. Moreover, compared with the Inj/*YAP1*^*KD*^ group, the Inj/*YAP1*^*OE*^ group displayed significantly increased expression of Col-III and CTGF, suggesting a more severe pathological state. Although Col-III expression decreased in the Inj/*YAP1*^*KD*^ group, Col-I levels were downregulated (versus Ctl/*NC* group, *P* < 0.05), suggesting that *YAP1* knockdown may not be an optimal strategy for treating pathological remodeling in tendinopathy.

Multiple-marker IF validation (Fig. 7c–f) indicated that at all time points, the proportions of *YAP1*^*+*^*TnC*^*+*^ and *sFRP2*^*+*^*TnC*^*+*^ tenocytes were significantly higher in the Inj/*YAP1*^*OE*^ group compared with the other three groups (*P* < 0.05); however, these proportions were markedly decreased in the Inj/*YAP1*^*KD*^ group (*P* < 0.05). In addition, IF staining confirmed that vascular density was significantly lower in the Inj/*YAP1*^*KD*^ group compared with the Inj/*NC* and Inj/*YAP1*^*OE*^ groups (*CD34* marker, *P* < 0.05) (Fig. 7g, h). Previous studies have shown that sFRP2 secreted by fibroblasts can activate the non-canonical Wnt/Ca^2+^ signaling pathway^50,51^, with phospholipase C-beta 1 (PLCB1) serving as a key regulatory factor. Activation of PLCB1 promotes endothelial angiogenesis^52^. We observed a certain correlation between PLCB1 and ATF6 signaling in endothelial cells and the expression of sFRP2 and YAP1. Compared with the Inj/*NC* group, the proportion of *CD34*^*+*^*PLCB1*^*+*^*ATF6*^*+*^ endothelial cells was significantly higher in the Inj/*YAP1*^*OE*^ group, while significantly lower in the Inj/*YAP1*^*KD*^ group (*P* < 0.05) (Fig. 7g, i). Activation of PLCB1 and ATF6 signaling may play a role in YAP1/sFRP2-mediated angiogenesis.

### Decreased FHL2 expression in activated tenocytes upregulated YAP1, which induces the expression and extracellular secretion of sFRP2

**Fig. 8:**
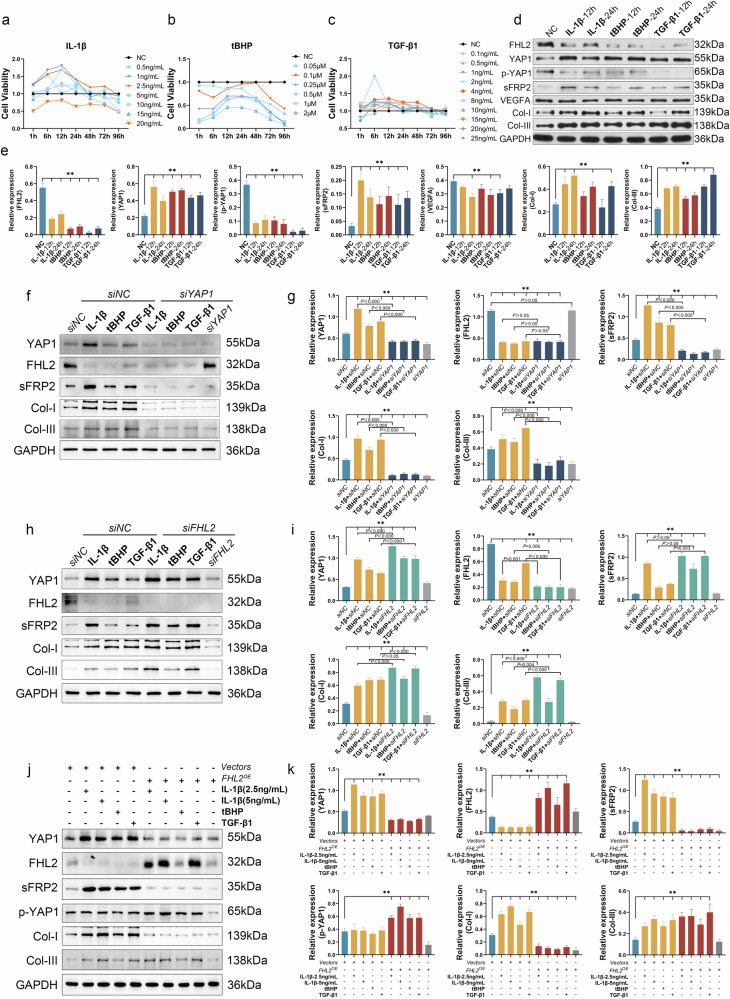
Analysis of the FHL2/YAP1/sFRP2 signaling axis in activated tenocytes. **a** Cell viability of tenocytes treated with IL-1β at gradient concentrations (CCK8). The 2.5 ng/ml concentration was identified as the optimal concentration. **b** Cell viability of tenocytes treated with tBHP at gradient concentrations (CCK8). The 0.1 μM concentration was identified as the optimal concentration. **c**, Cell viability of tenocytes treated with TGF-β1 at gradient concentrations (CCK8); 4 ng/ml was identified as the optimal concentration. **d**, **e** WB of key proteins (panel **d**) and relative expression levels (panel **e**) in tenocytes treated with IL-1β (2.5 ng/ml), tBHP (0.1 μM) and TGF-β1 (4 ng/ml) for 12 and 24 h (**P < 0.05 versus NC). **f**, **g** WB of key proteins (panel **f**) and relative expression levels (panel **g**) in tenocytes transfected with *siNC*/*siYAP1* and treated with IL-1β (2.5 ng/ml), tBHP (0.1 μM) and TGF-β1 (4 ng/ml) for 24 h (**P < 0.05 versus *siNC*). The specific *P* values comparing the *siNC* (+IL-1β/tBHP/TGF-β1) and *siYAP1* (+IL-1β/tBHP/TGF-β1) treatment groups are also presented. **h**, **i** WB of key proteins (panel **h**) and relative expression levels (panel **i**) in tenocytes transfected with *siNC*/*siFHL2* and treated with IL-1β (2.5 ng/ml), tBHP (0.1 μM) and TGF-β1 (4 ng/ml) for 24 h (**P < 0.05 versus *siNC*). The specific *P* values comparing the *siNC* (+IL-1β/tBHP/TGF-β1) and *siFHL2* (+IL-1β/tBHP/TGF-β1) treatment groups are also presented. **j**, **k** WB of key proteins (panel **j**) and relative expression levels (panel **k**) in tenocytes transfected with Vectors/*FHL2*^*OE*^ plasmids and treated with IL-1β (2.5 ng/ml), IL-1β^High-concentration^ (5 ng/ml), tBHP (0.1 μM) and TGF-β1 (4 ng/ml) for 24 h (**P < 0.05 versus Vectors).

Using stress models of tenocytes induced by IL-1β, TGF-β1 and tBHP, combined with *siYAP1*, *siFHL2* and *FHL2*^*OE*^ transfection, we investigated the regulatory mechanisms of FHL2/YAP1/sFRP2 in tenocytes. CCK8 assay showed that after treatment with IL-1β (Fig. 8a), tenocyte viability increased from 6 to 24 h, with the 2.5 ng/ml concentration demonstrating the highest activity with a long duration. Although the 1 ng/ml concentration exhibited slightly greater viability than 2.5 ng/ml at 12 h, its activity declined sharply afterward. Therefore, 2.5 ng/ml was selected for subsequent experiments. For treatment with tBHP (Fig. 8b), cell viability at all concentrations was lower than that of the NC group, with 0.1 μM concentration demonstrating higher overall activity compared with other concentrations. Thus, the 0.1 μM concentration was chosen for further experiments. After treatment with TGF-β1 (Fig. 8c), tenocyte viability showed minimal variation across concentrations, with most treatment concentrations resulting in slightly higher viability than the NC group from 6 to 48 h. The 4 ng/ml concentration showed the highest overall viability, while both lower and higher concentrations resulted in reduced viability. Therefore, the 4 ng/ml concentration was selected for subsequent experiments.

In WB (Fig. 8d, e), tenocytes treated with IL-1β, TGF-β1 or tBHP exhibited a significant decrease in FHL2 and a significant increase in YAP1 and sFRP2 expression (versus NC group, *P* < 0.05), consistent with our clinical and in vivo findings. Moreover, p-YAP1 levels were significantly reduced across all treatment groups, confirming that YAP1 phosphorylation was inhibited, leading to increased nuclear translocation. Similarly, VEGFA expression was not increased in IL-1β-treated, TGF-β1-treated or tBHP-treated tenocytes and was slightly lower than that in the NC group after 24 h of treatment with IL-1β and tBHP and 12 h of treatment with TGF-β1 (*P* < 0.05). While Col-I expression increased after 24 hours of treatment with IL-1β, tBHP and TGF-β1, the expression of Col-III showed a significant increase, particularly in the 24-h TGF-β1 group. This suggests that the imbalance in Col-I and Col-III expression after tissue injury may be regulated by the combination of inflammation, oxidative stress and TGF-β1 overexpression.

After interfering with YAP1 expression (Figs. 8f, g), we observed that the expression of YAP1 and sFRP2 was significantly reduced in the IL-1β + *siYAP1*, TGF-β1 + *siYAP1*, tBHP + *siYAP1* and *siYAP1* treatment groups (*P* < 0.05), accompanied by a marked decrease in Col-I and Col-III levels (*P* < 0.05). This finding aligns with the in vivo results (Fig. 7a, b), suggesting that YAP1 may act as a common transcription factor for sFRP2, Col-I and Col-III. However, *YAP1* knockdown did not significantly affect FHL2 expression.

The expression of FHL2 in tenocytes treated with IL-1β, tBHP or TGF-β1 was inherently reduced. Its expression further decreased after *siFHL2* transfection, significantly increasing YAP1 and sFRP2 expression (IL-1β + *siFHL2* group > IL-1β + *siNC* group, tBHP + *siFHL2* group > tBHP + *siNC* group, TGF-β1 + *siFHL2* group > TGF-β1 + *siNC* group, *P* < 0.05) (Fig. 8h, i). Interestingly, in tenocytes not treated with IL-1β, tBHP or TGF-β1, *FHL2* knockdown alone (*siFHL2* group) did not increase YAP1 or sFRP2 expression (*P* > 0.05) but significantly reduced Col-I expression (*P* < 0.05). This suggests that the regulatory effect of the FHL2/YAP1/sFRP2 axis can only be observed in stressed tenocytes. Conversely, upon *FHL2* overexpression (*FHL2*^*OE*^ transfection) (Figs. 8j, k), YAP1 and sFRP2 expression was significantly reduced in activated tenocytes, accompanied by an increase in p-YAP1 expression (*P* < 0.05). Even when the concentration of IL-1β was increased to 5 ng/ml, it failed to upregulate YAP1 and sFRP2 expression. In summary, under stress conditions, FHL2 may function as an upstream signaling molecule of the YAP1/sFRP2 axis, whose downregulation enhances YAP1/sFRP2 expression in tenocytes.

We also analyzed apoptosis in stressed tenocytes (Supplementary Fig. 7a, b) and observed increased apoptosis rates in tenocytes treated with IL-1β, tBHP or TGF-β1, with significantly higher apoptosis rates observed after treatment with tBHP and TGF-β1 (*P* < 0.05). Interfering YAP1 expression with *siYAP1* further enhanced apoptosis, particularly it markedly promoted late apoptosis (*P* < 0.05). However, with FHL2 overexpression (*FHL2*^*OE*^), the total apoptosis rate (early + late apoptosis) of tenocytes treated with IL-1β, tBHP or TGF-β1 did not exhibit a significant increase (IL-1β + *FHL2*^*OE*^ versus IL-1β + Vectors, tBHP + *FHL2*^*OE*^ versus tBHP + Vectors, TGF-β1 + *FHL2*^*OE*^ versus TGF-β1 + Vectors) (*P* > 0.05). These findings suggest that decreasing YAP1/sFRP2 through *FHL2* overexpression, thereby alleviating pathological remodeling of the vascular pattern, may be a superior option compared with direct knockdown of *YAP1*. Subsequently, we employed ELISA to measure sFRP2 levels in the culture medium of stressed tenocytes (Supplementary Fig. 7c, d). The results showed that sFRP2 levels in the culture medium were significantly elevated in tenocytes treated with IL-1β, tBHP or TGF-β1, while *YAP1* interference decreased sFRP2 levels. This indirectly indicates that stressed tenocytes can release sFRP2 extracellularly.

### Tenocytes promoted endothelial cell migration and angiogenesis via sFRP2

**Fig. 9:**
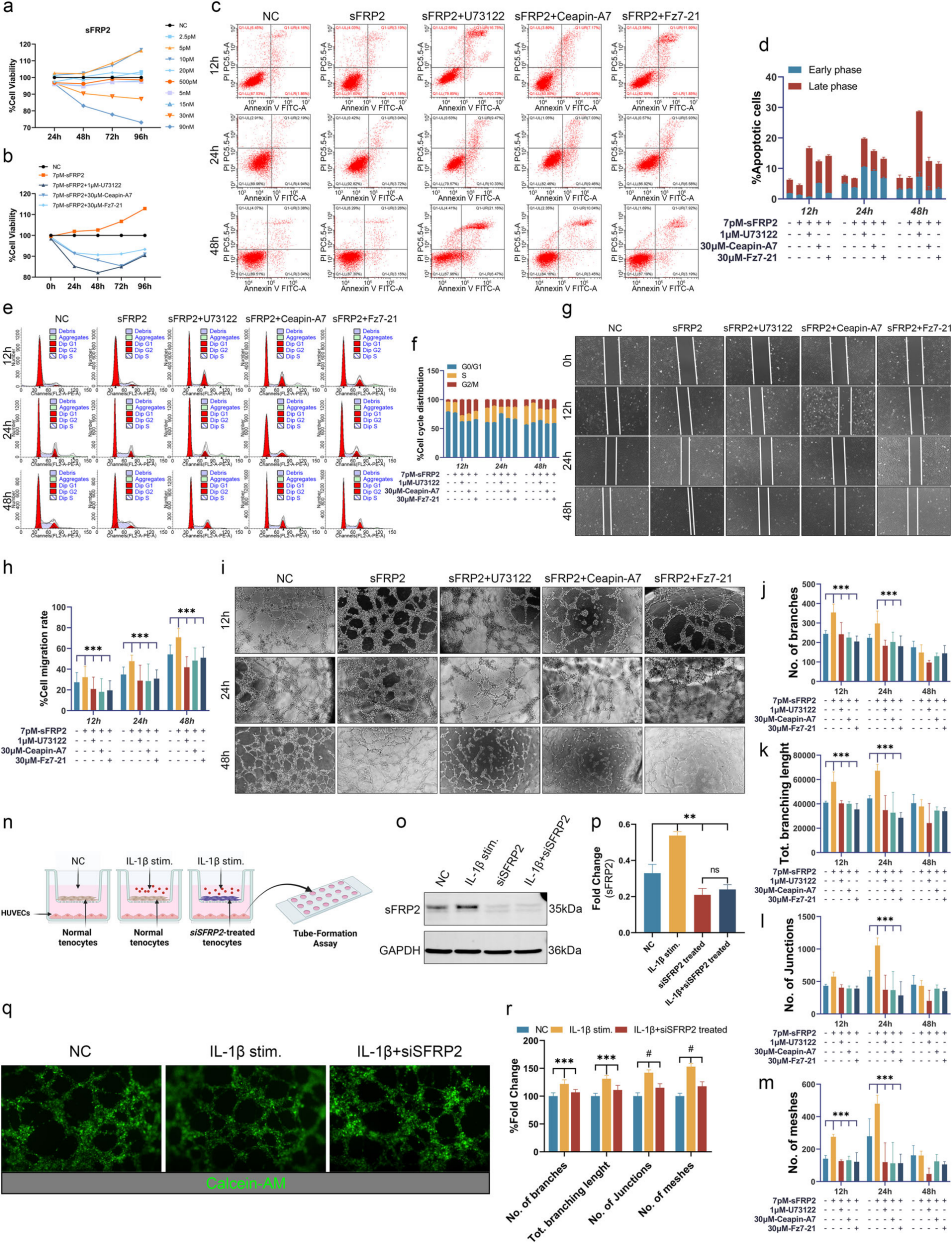
The effects of sFRP2 on the proliferation, migration and angiogenesis of HUVECs. **a** Cell viability of HUVECs treated with gradient concentrations of sFRP2 protein (CCK8). The 5–10 pM concentration showed the most significant effect. **b** Cell viability (CCK8) of HUVECs treated with sFRP2 + U73122 (phospholipase C inhibitor) or Ceapin-A7 (selective ATF6α signaling blocker) or Fz7-21 (FZD7 receptor peptide antagonist). **c**, **d** Flow cytometry analysis (panel **c**) of apoptosis in HUVECs treated with sFRP2 (7 pM) + U73122 (1 μM)/Ceapin-A7 (30 μM)/Fz7-21 (30 μM), with the percentage of cells exhibiting early apoptosis (LR) and late apoptosis (UR); the relevant statistical results displayed in panel **d**. **e**, **f** Flow cytometry analysis (panel **e**) of cell cycle in HUVECs treated with sFRP2 (7 pM) + U73122 (1 μM)/Ceapin-A7 (30 μM)/Fz7-21 (30 μM), with the percentages of cells in the G0/G1, S and G2/M phases; the relevant statistical results displayed in panel **f**. **g**, **h** Scratch wound healing assay (panel **g**); %Cell migration rate = (initial width at 0 h − width at specific time point)/initial width at 0 h, ***P < 0.05 versus the sFRP2-treated group; the relevant statistical results displayed in panel **h**. **i**–**m** Tube formation assay (panel **i**) with the number of branches (statistical results in panel **j**), total branching length (statistical results in panel **k**), number of junctions (statistical results in panel **l**) and number of meshes (statistical results in panel **m**), ***P < 0.05 versus the sFRP2-treated group. **n** Tenocyte-HUVECs coculture models. **o**, **p** WB (panel **o**) assessing the expression levels of sFRP2 in tenocytes transfected with *siSFRP2*, **P < 0.05 versus NC, ns: *P* > 0.05; the relevant statistical results displayed in panel **p**. **q** Tube formation assay of HUVECs after coculturing with tenocytes (Calcein-AM staining) (Supplementary Data File 1.3.3). **r** The number of branches, total branching length, number of junctions and number of meshes, ***P < 0.05 versus IL-1β-stimulated normal tenocyte-HUVECs coculture group, ^#^*P* < 0.05 between any two groups.

Clinical and in vivo findings revealed that sFRP2 overexpression is associated with angiogenesis and may affect the Wnt signaling pathway, PLCB1 and ATF6. We employed inhibitors U73122, Ceapin-A7 and Fz7-21 to measure the proangiogenic effects of sFRP2 on HUVECs. The CCK8 assay (Fig. 9a) revealed that sFRP2 promoted HUVEC viability at relatively low concentrations, with a narrow effective concentration window. Significant proviability effects were observed within the 5–10 pM concentration range. Treatment with U73122, Ceapin-A7 and Fz7-21 markedly suppressed the proviability effects of sFRP2, and the U73122-treated group exhibited the lowest overall cell viability (Fig. 9b).

Flow cytometry apoptosis assay (Fig. 9c, d) revealed no significant difference in total apoptosis (early + late apoptosis) between the sFRP2-treated group and the NC group (*P* > 0.05). However, treatment with sFRP2 significantly downregulated the early apoptosis of HUVECs at 24 h (*P* = 0.022). Treatments with U73122, Ceapin-A7 and Fz7-21 significantly increased the proportion of apoptotic cells, particularly the proportion of those with late apoptosis, which was markedly higher than that in the sFRP2-treated and NC groups (*P* < 0.05). At both 24 and 48 h, early and late apoptosis rates in the U73122-treated group were higher than those in the Ceapin-A7-treated and Fz7-21-treated groups (*P* < 0.05), consistent with the results of the CCK8 assay. This finding suggests that the PLCB1 signaling may play a critical role in HUVEC proliferation. Flow cytometry-cell cycle assay (Fig. 9e, f) revealed a significant increase in the G2/M phase proportion in the U73122-treated, Ceapin-A7-treated and Fz7-21-treated groups at 12 h (*P* < 0.05). At 24–48 h, the proportion of cells in the S phase significantly increased in the sFRP2 group but decreased in the U73122-treated, Ceapin-A7-treated and Fz7-21-treated groups (*P* < 0.05). Overall, at all time points, the proportion of cells in the G2/M phase was higher in the U73122-treated, Ceapin-A7-treated and Fz7-21-treated groups than in the sFRP2 group (*P* < 0.05), suggesting that the three inhibitors may induce G2/M phase arrest, necessitating future studies.

Scratch wound healing assay (Fig. 9g, h) demonstrated that at all time points, HUVEC migration rates in the sFRP2-treated group were significantly higher than those in other groups (*P* < 0.05), suggesting the pronounced promigratory effect of sFRP2 on HUVECs. Although migration rates in the U73122-treated, Ceapin-A7-treated and Fz7-21-treated groups were significantly lower than those in the sFRP2-treated group (*P* < 0.05), they were not significantly different from those in the NC group (*P* > 0.05).

Tube formation assay (Figs. 9i–9m) revealed that in the sFRP2-treated group, the number of branches, total branching length, the number of meshes at 12 and 24 h and the number of junctions at 24 h were significantly higher than those in other groups (*P* < 0.05). However, the number of branches and total branching length in the U73122-treated, Ceapin-A7-treated and Fz7-21-treated groups showed no significant differences compared with the NC group (*P* > 0.05). Furthermore, at 24 h, the number of meshes in the inhibitor-treated groups was slightly lower than that in the NC group (*P* < 0.05). No significant differences were observed among the U73122-treated, Ceapin-A7-treated and Fz7-21-treated groups (*P* > 0.05). These findings suggest that sFRP2 significantly promoted HUVEC tube formation while targeting FZD7 receptors, phospholipase C and ATF6 effectively inhibited this process. Notably, by 48 h, the collapse of tube structures was microscopically observed in all groups. At this time point, no significant differences in the number of branches, total branching length, number of junctions or number of meshes were detected among the groups (*P* > 0.05).

Subsequently, we employed tenocyte-HUVECs coculture models to determine the interaction between tenocytes and HUVECs (Fig. 9n). First, an *sFRP2*^*KD*^ tenocyte model was established using *siSFRP2* transfection. WB (Fig. 9o, p) confirmed that sFRP2 expression was significantly decreased in *siSFRP2*-transfected tenocytes, even after treatment with IL-1β, suggesting successful model construction. Tube formation assay (Fig. 9q, r) revealed that under IL-1β stimulation, coculturing normal tenocytes with HUVECs significantly enhanced the tube-forming ability of HUVECs, evidenced by a notable increase in the number of branches, total branching length, number of junctions and number of meshes (*P* < 0.05). By contrast, coculturing *sFRP2*^*KD*^ tenocytes with HUVECs suppressed the tube formation ability of HUVECs. The number of branches and total branching length showed no significant differences compared with the NC group (*P* > 0.05). These findings suggest that activated tenocytes can enhance the angiogenic activity of HUVECs by releasing sFRP2.

### Postinjury *FHL2* overexpression alleviated vascular remodeling and improved vascular perfusion function

**Fig. 10:**
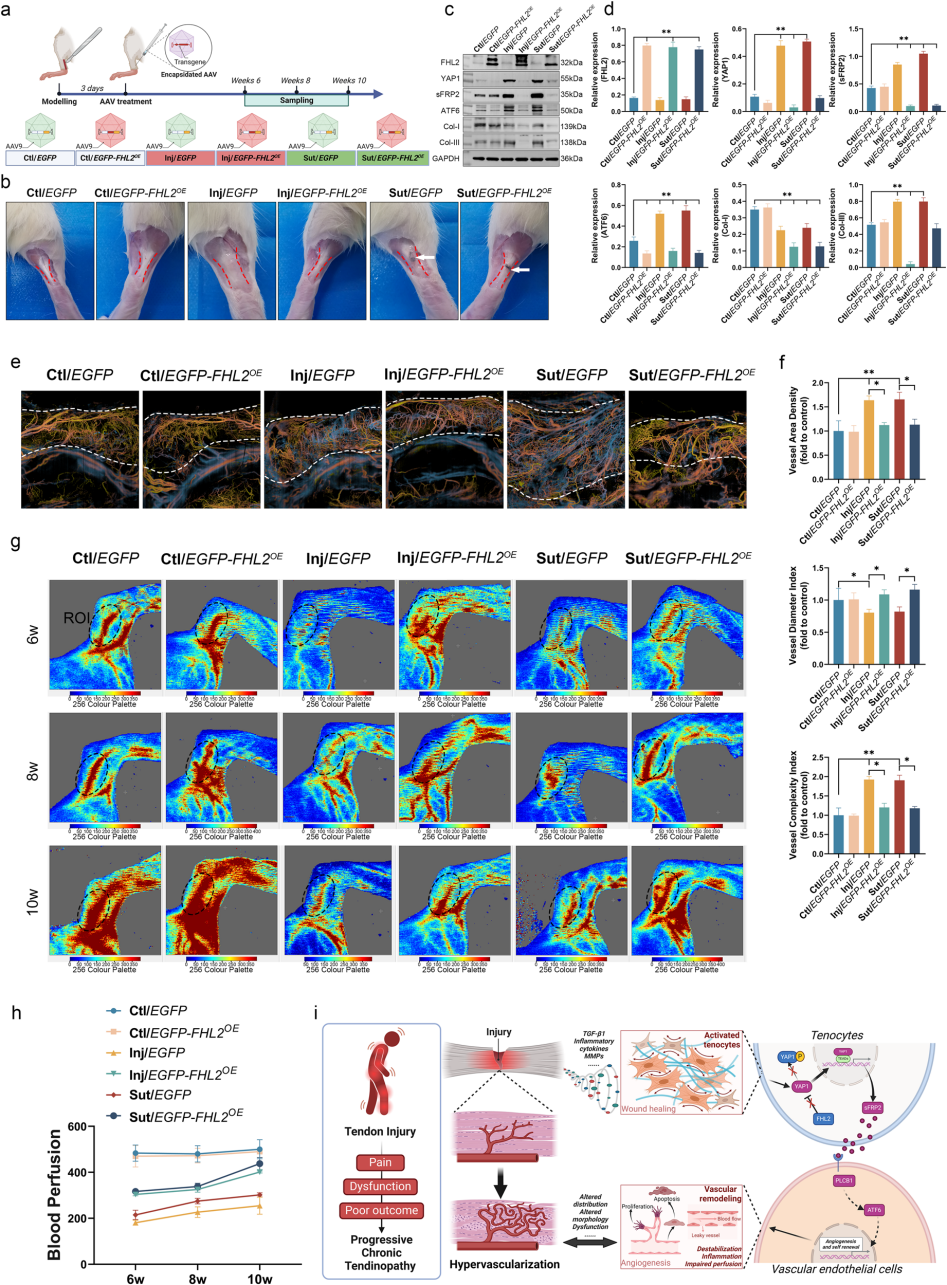
*FHL2* overexpression effectively alleviated vascular remodeling and improved vascular perfusion. **a** Construction of rat tendon injury (Inj)/suture (Sut) models, with AAV-*EGFP* (negative control) or AAV-*EGFP-FHL2*^*OE*^ transfection on day 3 after injury. The efficacy of *FHL2* overexpression in mitigating the progression of tendinopathy was measured at 6, 8, and 10 weeks after transfection (Supplementary Data File 1.4.1). **b** Gross specimens of rat tendon tissue at 10 weeks, with red dashed lines indicating the tendon region and white arrows indicating sutures. **c**, **d** WB of key proteins (panel **c**) and relative expression levels (panel **d**) at 10 weeks after transfection (**P < 0.05 versus Ctl/*EGFP*). **e** OCTA after 10 weeks, with the dashed lines indicating the tendon region (region of interest, ROI). **f** Statistical charts of VAD, VDI and VCI (**P < 0.05 versus Ctl/*EGFP*, *P < 0.05). **g** Laser speckle contrast imaging, with the black dashed circles indicating the ROI. **h** A statistical chart of average blood perfusion. **i** A schematic diagram of the mechanisms underlying vascular remodeling in tendinopathy, with the dashed arrows indicating undefined regulatory relationships.

This study revealed that low FHL2 expression-induced upregulation of YAP1/sFRP2 may be a critical mechanism underlying vascular remodeling in tendinopathy. However, direct *YAP1* knockdown increased tenocyte apoptosis and reduced Col-I expression (Figs. 7a and 8f and Supplementary Fig. 7a). Therefore, we employed a tendon injury/suture model combined with AAV-mediated gene intervention to measure the efficacy and feasibility of *FHL2* overexpression as a therapeutic strategy (Fig. 10a). IF of cryosections conducted six weeks after modeling (Supplementary Fig. 8a) demonstrated that encapsidated AAVs were successfully transfected into tendon tissue cells, exhibiting high transfection efficiency. At 10 weeks, gross specimens of rat tendon tissues (Fig. 10b) showed swelling, loss of tendon bundle structures and compromised tendon continuity in the Inj/*EGFP* and Sut/*EGFP* groups. By contrast, the Inj/*EGFP-FHL2*^*OE*^ and Sut/*EGFP-FHL2*^*OE*^ groups exhibited mild swelling and improved tissue continuity.

WB of rat tendon tissues 10 weeks after transfection (Fig. 10c, d) demonstrated that FHL2 expression was significantly increased in the Ctl/*EGFP-FHL2*^*OE*^, Inj/*EGFP-FHL2*^*OE*^ and Sut/*EGFP-FHL2*^*OE*^ groups (*P* < 0.05). Moreover, YAP1 and sFRP2 levels in the Inj/*EGFP-FHL2*^*OE*^ and Sut/*EGFP-FHL2*^*OE*^ groups were significantly lower than those in the Inj/*EGFP* and Sut/*EGFP* groups (*P* < 0.05), while there was no significant difference between the Ctl/*EGFP-FHL2*^*OE*^ and Ctl/*EGFP* groups (*P* > 0.05). These results confirmed that the regulatory role of FHL2 on YAP1/sFRP2 can be observed primarily when tenocytes become activated after tissue injury. The expression of ATF6 was significantly decreased in the Ctl/*EGFP-FHL2*^*OE*^, Inj/*EGFP-FHL2*^*OE*^ and Sut/*EGFP-FHL2*^*OE*^ groups, which was even less than that of the Ctl/*EGFP* group (*P* < 0.05). However, Col-I expression decreased when Col-III levels were significantly decreased in the Inj/*EGFP-FHL2*^*OE*^ and Sut/*EGFP-FHL2*^*OE*^ groups (*P* < 0.05). This suggests that the ‘injury’ factor may exert a greater effect on collagen expression.

IHC (Supplementary Fig. 8b, c) revealed no significant difference in IL-1β expression between the Ctl/*EGFP-FHL2*^*OE*^ and Ctl/*EGFP* groups (*P* > 0.05). By contrast, IL-1β levels in the Inj/*EGFP-FHL2*^*OE*^ and Sut/*EGFP-FHL2*^*OE*^ groups were significantly lower than those in the Inj/*EGFP* and Sut/*EGFP* groups (*P* < 0.05). These findings indicated that *FHL2* overexpression significantly reduced IL-1β levels after tendon injury.

In hematoxylin–eosin staining (Supplementary Fig. 8d), the extended evaluation period revealed an increased presence of calcified foci in the Inj/*EGFP* and Sut/*EGFP* groups. Moreover, pathological features, such as disordered and irregular tissue structure, fiber breakage, loss of bundle structures and angiogenesis persisted without improvement, validating progression into the chronic deterioration phase of tendinopathy. In the Inj/*EGFP-FHL2*^*OE*^ and Sut/*EGFP-FHL2*^*OE*^ groups, tissue structure became more organized over time, characterized by the emergence of bundle fiber structures. However, there was a noticeable gap between normal tendon tissue and the Ctl/*EGFP* group, there were no significant differences between the Inj/*EGFP-FHL2*^*OE*^ and Sut/*EGFP-FHL2*^*OE*^ groups.

Masson staining (Supplementary Fig. 8e, f) revealed no improvement in the compactness of collagen fiber structures in the Inj/*EGFP* and Sut/*EGFP* groups between week 6 and week 10, with %LCVF remaining elevated. By contrast, the Inj/*EGFP-FHL2*^*OE*^ and Sut/*EGFP-FHL2*^*OE*^ groups showed more distinct bundle fiber structures. At week 10, %LCVF in the Inj/*EGFP-FHL2*^*OE*^ and Sut/*EGFP-FHL2*^*OE*^ groups significantly decreased compared with week 6 (*P* < 0.05), suggesting a partial recovery in tissue compactness.

At week 10, OCTA (Fig. 10e, f) revealed that vascular distribution in the Inj/*EGFP-FHL2*^*OE*^ and Sut/*EGFP-FHL2*^*OE*^ groups appeared more organized than in the Inj/*EGFP* and Sut/*EGFP* groups. Compared with the Inj/*EGFP* and Sut/*EGFP* groups, the Inj/*EGFP-FHL2*^*OE*^ and Sut/*EGFP-FHL2*^*OE*^ groups revealed decreased VAD and VCI (*P* < 0.05), with no significant differences compared with the Ctl/*EGFP* group (*P* > 0.05). Meanwhile, VDI was increased in the Inj/*EGFP-FHL2*^*OE*^ and Sut/*EGFP-FHL2*^*OE*^ groups (versus Inj/*EGFP* and Sut/*EGFP* groups, *P* < 0.05). These findings indicated that *FHL2* overexpression inhibited the formation of excessive and complex blood vessels in tendinopathy.

Despite the extensive angiogenesis observed in the Inj/*EGFP* and Sut/*EGFP* groups, LSCI (Fig. 10g, h) exhibited that their average blood perfusion was decreased (versus Ctl/*EGFP* group, *P* < 0.05). By contrast, the Inj/*EGFP-FHL2*^*OE*^ and Sut/*EGFP-FHL2*^*OE*^ groups exhibited increased average blood perfusion compared with the Inj/*EGFP* and Sut/*EGFP* groups, although it remained significantly lower than that of the Ctl/*EGFP* group (*P* < 0.05). Perfusion progressively improved in the Inj/*EGFP-FHL2*^*OE*^ and Sut/*EGFP-FHL2*^*OE*^ groups over time, exhibiting minimal differences compared with the Ctl/*EGFP* group after 10 weeks. These findings highlight the beneficial role of *FHL2* overexpression in improving tissue perfusion after tendon injury.

## Discussion

The role of vascular networks in tendon repair remains unclear^1,21^. This study reveals that FHL2/YAP1/sFRP2-mediated vascular remodeling drives tendinopathy progression (Fig. 10i). Clinically, hypervascularization is correlated with poorer prognoses, including suboptimal postoperative recovery in terms of pain, function and strength. Pathological features (inflammatory infiltration, apoptosis and ECM disorganization) aligned with FHL2 downregulation and YAP1/sFRP2 upregulation. In injured tendons, activated tenocytes reduced FHL2 expression in response to inflammatory/oxidative stress, as well as TGF-β1 stimulation. FHL2 downregulation triggered YAP1 overexpression and nuclear translocation, promoting aberrant sFRP2 secretion. Elevated sFRP2 induced endothelial proliferation, migration and angiogenesis, driving pathological vascularization that disrupts tendon repair. The extensive formation and redistribution of spatially complex blood vessels disrupt tendon regeneration and repair, leading to the progression of tendon injuries into progressively worsening tendinopathy. *FHL2* overexpression effectively inhibited this remodeling, improved tissue structure and perfusion, and offered novel therapeutic insights.

The power Doppler imaging identified two chronic tendinopathy states in the study: rich and deficient blood flow signals. Similarly, Tang et al.^53^ observed subtypes characterized by downregulated versus upregulated angiogenesis/inflammation, with anti-inflammatory agents effective only in the latter—paralleling our HyperV subgroup findings of elevated inflammation and poorer outcomes. While vascularization strongly correlates with tendinopathy pain^9,11,54,55^ and angiogenesis inhibition mitigates progression^8^, angiogenesis remains beneficial for tendon repair in specific injury stages^4^. Thus, decoding dynamic vascular instability and identifying critical ‘uncontrolled factors’ in tendinopathy progression is clinically vital.

Contrary to traditional views linking VEGFA upregulation to angiogenesis^56–58^, VEGFA expression was lower in the HyperV than in the HypoV group. Notably, targeting VEGFA in diseases characterized by abnormal vascularization (for example, retinal disorders and tumors) often results in suboptimal efficacy, leading to rebound angiogenesis or damage to normal vasculature^59^. This suggests that other critical proangiogenic factors drive abnormal angiogenesis in tendinopathy. Bulk RNA-seq implicated TGF-β, Wnt and Hippo pathways in DEGs, highlighting TGF-β1, FHL2, YAP1 and sFRP2. Our prior phosphoproteomic analysis of chronic rotator cuff tendinopathy identified MST1 (Hippo/YAP1 upstream kinase) as a core kinase, with downregulated phosphosites enriched in muscle development^60^. Studies on skeletal muscle injury and remodeling have shown that YAP1 upregulation is closely associated with increased vascular density^32^, and inhibition of YAP1-mediated angiogenesis can partly reduce ectopic ossification in tendon injuries^61^. YAP1 overexpression in tendon fibroblasts enhances chromatin accessibility, mitigating matrix catabolism^62^, while persistent YAP1 activation converts fibroblasts to pathological cancer-associated fibroblasts^63^. In this study, abnormal YAP1 upregulation in the HyperV group correlated strongly with persistent pathological remodeling, FHL2 downregulation, and TGF-β1/sFRP2 upregulation.

This study employed exercise-induced (treadmill) and trauma-induced rat tendinopathy models to investigate the FHL2/YAP1/sFRP2 axis in the context of angiogenesis. The noninvasive treadmill model minimizes external interference but has lower reproducibility, whereas the trauma model examines the progression from acute to chronic conditions. An innovatively established tendon injury suture model further assessed the surgical prevention of tendinopathy development. Luo et al.^33^ reported that in overload exercise-induced tendinopathy models, moderate treadmill running (similar to the exercise intensity of the L-tm group in this study) induced adaptive tendon remodeling after 8 weeks with milder fiber damage or rupture. Similar to the H-tm group, intensive treadmill running increased the expression of adipogenic, chondrogenic, and osteogenic genes, and enhanced PGE2 production as early as 4 weeks, finally leading to chronic tendinopathy. Overload exercise models have unique advantages in simulating fatigue-related degenerative tendon injuries observed in clinical settings. In the early stages of tendinopathy (6–8 weeks), we observed pathological features, including partial fiber rupture and collagen remodeling, in both the L-tm and H-tm groups. In the later stage (12–18 weeks), the L-tm group exhibited adaptive physiological tendon remodeling, whereas the H-tm group exhibited increased vascular density and progressed to chronic tendinopathy characterized by disorganized fiber structures, increased apoptosis and increased Col-III/Col-I ratio. Although clinical studies have reported that intratendinous blood flow increases during normal tendon exercise^64^, angiogenesis is more likely to induce pain than blood flow^10,65^. Furthermore, we found that FHL2 expression was increased during physiological tendon remodeling (L-tm), whereas FHL2 expression was substantially decreased under pathological conditions (H-tm), accompanied by increased TGF-β1/YAP1/sFRP2 levels. Previous studies in the musculoskeletal system have shown that FHL2 can reduce inflammation during tissue injury, enhance fibroblast activity and promote repair^42,66,67^. This suggests that FHL2 may serve as an important protective factor in tendon repair.

In the traumatic tendon injury models, the tendons exhibited pathological remodeling after 4–6–weeks, suggesting worsening tendinopathy. Suturing did not prevent the progression of severe tendon injury to chronic tendinopathy, suggesting that the extent of disruption in overall tendon continuity may not be the primary driver of tendinopathy. OCTA provided detailed vascular spatial mapping in the Td-Inj and Td-Sut groups, revealing pathological features of vascular remodeling, such as increased vascular abundance, redistribution into tendons, increased morphological complexity and decreased diameter of blood vessels. In rheumatoid arthritis with high vascular abundance, increased vascular complexity in synovial tissue has been linked to higher rates of rheumatoid factor positivity and erosive disease^23^. Studies on retinal vascularization and malignant tumors have shown that highly branched vascular structures during angiogenesis can decrease effective blood flow at the angiogenic front, potentially exacerbating tissue hypoxia^68,69^. Orr et al.^70^ reported that vascular branching networks can help define overall physical structures and simultaneously regulate surrounding cellular properties, with vascular development playing a critical role in reshaping tissue morphology in normal organs. While the specific roles or relative contributions of vascular abundance, morphology, and distribution in tendinopathy remain unclear, pathological vascular remodeling leads to the progression of tendinopathy. Despite the observed vascular ingrowth during tendon repair, it remains unresolved whether pathological progression in tendinopathy stems from insufficient neovessel pruning, impaired perfusion, or aberrant endothelial cell phenotypic shifts, underscoring the critical need to link spatial tendon heterogeneity^27,49,71^ with vascular remodeling dynamics systematically.

Sequential changes in the expression of the FHL2/YAP1/sFRP2 axis were observed in the temporal remodeling of vascular patterns. In the acute phase of tendon injury (from 3 days to 1 week), FHL2 exhibited a transient expression peak, accompanied by an increased proportion of *FHL2*^*+*^ tenocytes. FHL2 signaling substantially decreased over time, accompanied by aberrant increases in YAP1 and sFRP2 expression, including the increased proportion of *YAP1*^*+*^ and *sFRP2*^*+*^ tenocytes. In AAV-transfected *YAP1*^*KD/OE*^ models, we observed that *YAP1*^*KD*^, which decreased sFRP2 levels without affecting FHL2, substantially inhibited vascular remodeling and improved structural remodeling. By contrast, *YAP1*^*OE*^, which increased sFRP2 levels without affecting FHL2, exhibited the opposite effect. This finding indicates that YAP1-mediated increase in sFRP2 levels in tenocytes may be a key mechanism promoting vascular remodeling. Previously, studies in melanoma revealed that sFRP2 can promote angiogenesis by inhibiting VEGF expression^44^. Similarly, studies on diabetic wound healing reported that suppressing sFRP2 expression can noticeably inhibit angiogenesis and tissue remodeling^72^. The proangiogenic effects of sFRP2 are believed to be mediated by the activation of the non-canonical Wnt signaling pathways^73^. Our study also identified a significant increase in PLCB1 signaling, a critical factor in the non-canonical Wnt/Ca^2+^ pathway. Moreover, sFRP2 plays a dual and concentration-dependent role (promotive or inhibitory) in fibrosis and angiogenesis^51^. However, the role and mechanism behind the concentration-dependent effects of sFRP2 in tendon vascular remodeling remain unclear. sFRP2 expression in tendinopathy is associated with the Hippo/YAP1 signaling pathway. YAP1 is crucial for the regulation of cell proliferation, survival and differentiation, organ development, homeostasis and regeneration^74^. From the perspective of tissue repair, increased YAP1 expression in the early stages of injury may facilitate cell proliferation and regeneration and promote tissue repair. In this study, we observed a progressive increase in YAP1 expression over time, suggesting the disruption of the Hippo signaling pathway. This imbalance may lead to excessive gain of function in tenocytes, a critical pathological feature of tendon vascular pattern remodeling.

Subsequently, we employed tenocyte stress models and coculture models with HUVECs to investigate the mechanisms behind YAP1 imbalance and unravel the interactions between tenocytes and endothelial cells. Tenocytes treated with IL-1β, TGF-β1 or tBHP consistently exhibited a marked decrease in FHL2 expression and a significant increase in YAP1 and sFRP2 expression. Moreover, interventions with siRNA and plasmids indicated that reduced FHL2 expression in tenocytes can induce YAP1-mediated upregulation of sFRP2. Furthermore, ELISA of the culture medium revealed that activated tenocytes secreted sFRP2 extracellularly (Supplementary Fig. 7c, d), serving as a bridge for interactions between tenocytes and endothelial cells. Subsequently, a coculture model using HUVECs and Transwell chambers (Fig. 9) demonstrated that tenocyte-secreted sFRP2 promoted the proliferation, migration and angiogenesis of endothelial cells. Although this study revealed the regulatory role of tenocytes in mediating angiogenesis through the FHL2/YAP1/sFRP2 axis, the presence of a negative feedback loop cannot be excluded. For instance, previous studies have indicated that sFRP2 may either promote or inhibit fibrosis^50,51^. Moreover, interventions targeting the Wnt pathway/FZD7 receptor, PLCB1 and ATF6 in HUVECs noticeably inhibited the proangiogenic effects of sFRP2. Studies have shown that the FZD7 receptor signaling in the Wnt pathway is essential for postnatal angiogenesis^75^, and the loss of pericyte FZD7 in pulmonary hypertension was shown to impair microvascular formation^76^. PLCB1 and ATF6, identified as differentially expressed factors in this study, have been reported to be associated with angiogenesis^45,52^. Therefore, we speculated that the proangiogenic effects of sFRP2 may be associated with the activation of the Wnt/FZD7 receptor/PLCB1/ATF6 signaling axis. Moreover, Courtwright et al.^73^ demonstrated that sFRP2 also promotes angiogenesis by activating the calcineurin/NFAT signaling pathway, suggesting that the proangiogenic actions of sFRP2 operate through a complex regulatory network. However, since our study did not investigate the crosstalk between the FZD7 receptor/PLCB1/ATF6 axis and the calcineurin/NFAT signaling pathway, the precise molecular mechanism underlying sFRP2-mediated angiogenesis remains unclear.

This study identified two approaches to reduce sFRP2 expression: *FHL2* overexpression and *YAP1* knockdown. However, we found that *YAP1* knockdown increases apoptosis (Supplementary Fig. 7a, b), suggesting that YAP1, as a key regulator of several growth and developmental signaling pathways, may not be an optimal therapeutic target for treating tendinopathy. Therefore, we developed a therapeutic strategy based on AAV-*FHL2*^*OE*^. Our findings demonstrated that *FHL2* supplementation after tendon injury can noticeably decrease YAP1/sFRP2 expression, inhibit vascular remodeling associated with tendinopathy and restore tissue structure despite some differences from normal tendon tissue. Interestingly, we observed that the formation of complex blood vessels in tendinopathy does not enhance blood flow. LSCI analysis revealed that blood perfusion levels remained less than normal even 10 weeks after injury (Fig. 10g, h), while *FHL2* overexpression noticeably improved vascular perfusion levels. These results indicated the feasibility and efficacy of AAV-*FHL2*^*OE*^ in treating tendinopathy. Nevertheless, low targeting specificity in this intervention modality is challenging. In addition to its effects on tenocytes, AAV vectors may act on other cell types involved in vascular remodeling, such as vascular endothelial cells and macrophages. The precise effects of these factors on the therapeutic efficacy of gene intervention remain unclear.

However, studies in other contexts report proangiogenic roles for FHL2. In corneal injury, elevated FHL2 expression induces angiogenesis^77^. Similarly, cancer-associated fibroblast-derived FHL2 promotes angiogenesis and metastasis in lung adenocarcinoma by activating osteopontin secretion^78^. Huang et al. further demonstrated that FHL2 deficiency impairs proangiogenic cell mobilization, hindering neovascularization and compromising blood perfusion^79^. Normalizing blood perfusion critically depends on the maturation of vascular cell phenotypes and luminal vasomotor function. Indeed, FHL2 regulates vascular tone by modulating the phenotypic switching of vascular smooth muscle cells^80^, which aligns with our observation that FHL2 overexpression improves tendon blood perfusion. Therefore, the reduced vessel abundance and complexity observed here probably reflect the unique role of FHL2 within the specific inflammatory microenvironment of tendinopathy. FHL2 is a well-established anti-inflammatory and protective factor in tissue repair and arthritis^42,66^. Thus, in tendinopathy, *FHL2* overexpression may primarily suppress inflammatory angiogenesis via its anti-inflammatory effects, thereby improving perfusion and promoting repair, potentially mediated by the YAP1/sFRP2 axis. Collectively, these findings suggest that FHL2 exerts context-dependent dual roles in regulating angiogenesis. Its anti-inflammatory, vessel-stabilizing function in tendinopathy contrasts with its proangiogenic actions in corneal repair or tumor microenvironments. Elucidating the precise mechanisms governing the vascular effects of FHL2 across different pathophysiological conditions is crucial for understanding its functional diversity and therapeutic targeting.

This study focused on vascular homeostasis and revealed that vascular spatial pattern remodeling is critically involved in the development and progression of tendinopathy. It also unraveled the mechanism by which tenocytes regulate angiogenesis through the FHL2/sFRP2 axis and demonstrated the feasibility and efficacy of *FHL2* overexpression in treating vascular remodeling. These findings provide novel theoretical insights into the mechanisms of chronic tendinopathy and offer new avenues for clinical diagnosis and treatment. However, this study has certain limitations: (1) due to technical constraints and cost-effectiveness considerations, this study did not obtain spatial information on tendon vasculature of patients, including critical parameters such as vascular spatial distribution and morphology. Future advancements in noninvasive and nonradiative vascular spatial imaging technologies will dramatically facilitate tendon vascular research. (2) This study did not thoroughly analyze the regulatory relationships within the sFRP2/PLCB1/ATF6 signaling axis in endothelial cells. Further studies are needed to elucidate how these signals affect the functional phenotypic transitions of endothelial cells. (3) Although *FHL2* overexpression alleviated vascular spatial remodeling and partly improved vascular perfusion, the pathological relationship between vascular spatial remodeling and perfusion function necessitates further studies.

In conclusion, this study systematically explored the progression of tendinopathy and its pathological relationship with vascular instability, highlighting the critical role of vascular remodeling via the FHL2/YAP1/sFRP2 signaling axis. Chronic tendinopathy with excessive vascular proliferation was associated with poor clinical outcomes, characterized by persistently high pain scores, impaired function, and reduced upper limb strength. Using a rat model and in vitro experiments, this study demonstrated that the formation and redistribution of numerous morphologically complex blood vessels are key pathological factors driving the progression of tendinopathy. The low expression of FHL2 and the high expression of YAP1 and sFRP2 were identified as pivotal mechanisms. Under stress conditions, low FHL2 expression in tenocytes activated YAP1, thereby inducing sFRP2 expression and secretion, and promoting endothelial cell proliferation, migration, and angiogenesis. Gene therapy targeting *FHL2* improved tendon tissue structure and blood perfusion and alleviated vascular remodeling. These findings provide a theoretical basis for developing novel therapeutic strategies, demonstrating critical clinical value for clinical practice.

## Supplementary information

Supplementary Information Supplementary Data Files 1–4, Figs. 1–8, Tables 1 and 2.

## Data Availability

All data are available in the main text or the Supplementary Information.
