# Supplementary material for: Temporal vascular pattern remodeling mediated by the FHL2/sFRP2 signaling pathway in tenocytes affects tendon repair and regeneration
Source: Exp Mol Med. 2025 Nov 11;57(11):2533–58. doi: 10.1038/s12276-025-01574-2 (PMC12686517; doi:10.1038/s12276-025-01574-2)
Supplement: Supplementary file 1 — Supplementary Information Supplementary Data Files 1–4, Figs. 1–8, Tables 1 and 2. [file 12276_2025_1574_MOESM1_ESM.pdf]

Data File S1

1. Clinical Study

1.1. Patient cohort and clinical follow-up

All ultrasonographic assessments in the clinical study were conducted with the assistance of the same senior physician from the radiology department. Surgical procedures and intraoperative sampling were conducted by senior attending surgeons of the team using standard techniques, without involving new materials or procedures. All patients underwent identical rehabilitation training. The protocol followed the guidelines and regulations of the Shanghai Medical Ethics Committee and was approved by the Shanghai Tenth People's Hospital Ethics Committee.

The inclusion criteria for the HyperV group were as follows: (1) being diagnosed with chronic rotator cuff tendinopathy accompanied by full-thickness rotator cuff tears (primarily involving the supraspinatus tendon) and undergoing arthroscopic rotator cuff repair and shoulder joint debridement; (2) symptoms persisted for at least 12 months; (3) body mass index (BMI) between 18.5 and 25 kg/m^2 and aged 30-65 years; and (4) Adler's grading II-III based on shoulder Power Doppler imaging.

The inclusion criteria for the HypoV group were as follows: (1) being diagnosed with chronic rotator cuff tendinopathy accompanied by full-thickness rotator cuff tears (primarily involving the supraspinatus tendon) and undergoing arthroscopic rotator cuff repair combined with shoulder joint debridement; (2) symptoms persisted for at least 12 months; (3) BMI between 18.5 and 25 kg/m^2 and aged 30-65 years; and (4) Adler's grading 0-I based on shoulder Power Doppler imaging.

The inclusion criteria for the normal group were as follows: (1) being diagnosed with anterior cruciate ligament injuries and undergoing arthroscopic anterior cruciate ligament reconstruction (using autologous hamstring tendons); (2) no evidence of shoulder joint or rotator cuff disorders; and (3) BMI between 18.5 and 25 kg/m^2 and aged 30-45 years.

The exclusion criteria for the HyperV and HypoV groups were as follows: (1) massive irreparable rotator cuff tears; (2) history of severe shoulder trauma, steroid/immunosuppressant use, or shoulder surgery; (3) history of tumors, infections, or severe systemic diseases; (4) neurological or psychiatric disorders not allowing physical examination; (5) incomplete medical or imaging records or noncompliance with medical interventions or follow-up; (6) severe congenital deformities of the upper limbs; and (7) recent intra-articular shoulder injections on the affected side.

The exclusion criteria for the normal group were as follows: (1) history of severe knee trauma, steroid/immunosuppressant use, or knee surgery; (2) history of severe shoulder trauma, steroid/immunosuppressant use, or shoulder surgery; (3) history of tumors, infections, or severe systemic diseases; (4) neurological or psychiatric disorders not allowing physical examination; (5) incomplete medical or imaging records or noncompliance with medical interventions or follow-up; and (6) severe congenital deformities of the upper or lower limb.

Upper limb strength testing method: A real-time force gauge was attached at the wrist while maintaining the Jobe's test position (arm abducted 90°, horizontally adducted 30°, internally rotated 90°, elbow extended, and thumb pointing downward).

Patients maximally resisted the examiner's downward pull. Maximum resistance force and endurance time were recorded for both affected and healthy sides. The differences in strength and resistance time between the affected and healthy sides were calculated for analysis.

1.2 Sampling and histological assessment

Tissues designated for Bulk RNA sequencing (RNA-seq) and western blotting (WB) were preserved by liquid nitrogen freezing. A portion of the tendon tissues was fixed in 4% paraformaldehyde, embedded in paraffin, and sectioned longitudinally (4 μm thickness). The sections were subjected to routine pathological staining. WB was conducted in six replicates, and all types of histological staining were conducted with eight replicates per sample.

Hematoxylin-eosin staining (HE): Paraffin sections were deparaffinized and sequentially processed with hematoxylin solution (G1005, Servicebio), hematoxylin differentiation solution (G1039, Servicebio), hematoxylin Scott tap bluing (G1040, Servicebio), and eosin dye (G1005, Servicebio). The sections were then dehydrated, mounted, and observed under a microscope.

Alcian blue staining (AB): Paraffin sections were deparaffinized and stained with AB dye (G1027, Servicebio). After dehydration and mounting, acidic mucinous substances appeared blue under microscope.

Sirius red staining (SR): Paraffin sections were deparaffinized and stained using a modified SR staining kit (G1078, Servicebio). After dehydration and mounting, the sections were observed under a polarized light microscope (NIKON Eclipse ci). Under polarized light, Col-I appeared as orange or bright red thick fibers, while Col-III appeared as green thin fibers. Four distinct fields of view were captured at 40× magnification for each sample. Image-Pro Plus 6.0 software was used to calculate the ratio of *Col-III* to *Col-I*, and the mean value of four fields was recorded as the final ratio for each sample.

TdT-mediated dUTP nick-end labeling assay (TUNEL): Paraffin sections were deparaffinized and stained using a TUNEL assay kit (G1501-50T, Servicebio). The sections were observed under a fluorescence microscope, and images were captured for analysis (nuclei stained blue; apoptotic cells stained green). For each sample, six distinct fields of view were analyzed at 60× magnification. The percentage of apoptotic cells in each field was calculated using ImageJ software (% = apoptotic cells/total cells). The average value of the six fields was considered the apoptosis rate for each sample.

Immunohistochemistry (IHC): Paraffin sections were prepared for routine IHC staining to detect IL-1β (GB11113, Servicebio), IL-6 (TD6087, Abmart), TNF-α (PY19810, Abmart), VEGFA (TA5131, Abmart), CD34 (GB15013, Servicebio), FHL2 (TD13015, Abmart), YAP1 (A19134, ABclonal), and sFRP2 (TD4451, Abmart). Sections were deparaffinized to water and subjected to antigen retrieval using citrate buffer solution (pH=6.0, G1219, Servicebio). Endogenous peroxidase activity was blocked using 3% hydrogen peroxide solution, followed by blockade with 3% BSA solution or 10% rabbit serum. Thereafter, the sections were incubated with the primary antibodies and then incubated with HRP-labeled secondary antibodies. DAB

staining (G1212, Servicebio) was conducted for visualization, and counterstaining of nuclei was conducted. The sections were then dehydrated and mounted, and images were captured. For each sample, four distinct fields of view were captured at 20× magnification. The average optical density (AOD) was quantified using Image-Pro Plus 6.0 software, with the mean value of these four fields recorded as the semi-quantitative expression level of the marker for the sample. For CD34 staining, the number of blood vessels was counted in 4 distinct fields of view at 20× magnification (number/0.5 mm^2). The average value of these fields was recorded as the semi-quantitative vascular density for the sample.

Immunofluorescence (IF): IF staining was conducted to measure the proportion of positive cells, including *TGF-β1*$^+$, *FHL2*$^+$*TnC*$^+$, *YAP1*$^+$*TnC*$^+$, and *sFRP2*$^+$*TnC*$^+$*vWF*$^-$ cells. Primary antibodies included TGF-β1 (A22296, ABclonal), FHL2 (TD13015, Abmart), TnC (A18156, ABclonal), YAP1 (A19134, ABclonal), sFRP2 (TD4451, Abmart), and vWF (ab6994, Abcam). Paraffin sections were deparaffinized to water and subjected to antigen retrieval using EDTA buffer (pH=8.0, G1207, Servicebio). Sections were treated with 3% hydrogen peroxide and then blocked with 3% BSA solution or 10% rabbit serum. Primary and HRP-labeled secondary antibodies were then incubated sequentially. For multiplex IF staining, TSA kits (G1226/G1236, Servicebio) were used for processing. Finally, the nuclei were counterstained with DAPI, autofluorescence was quenched, and the sections were mounted. Imaging was conducted using filters specific to the excitation and emission wavelengths of the fluorescent dyes. Six distinct fields of view at 60× magnification were captured for each sample. The percentage of positive cells (% = positive cells/total cells) in each field was calculated using ImageJ software, and the mean percentage across the six fields was recorded as the positive cell rate for the sample.

WB: Semi-quantitative analysis of the expression of FHL2 (TD13015, Abmart), YAP1 (A19134, ABclonal), and sFRP2 (TD4451, Abmart) was conducted in the HypoV and HyperV groups. The protein sample was extracted using grinding and RIPA lysis buffer (SD0005/SD0007, Simuwubio) supplemented with protease inhibitors (SD0001, Simuwubio). Protein concentration was determined using a BCA protein assay kit (SD0012, Simuwubio). Denatured proteins were subjected to SDS-PAGE, followed by transfer to PVDF membranes (G6044, Servicebio). Immunoreactions were conducted using primary and secondary antibodies, and signals were detected using ECL chemiluminescence (G2020, Servicebio). The grayscale values of the target bands were quantified using ImageJ software. GAPDH served as the loading control, and the ratio of the target band intensity to the GAPDH band intensity was used for analysis.

1.3 Bulk RNA-seq

RNA was extracted from samples using Trizol (Thermo Fisher Scientific, 15596018). Libraries were prepared using the NEBNext Ultra RNA Library Prep Kit (NEB #E7490), and purified with beads (AMPure XP system). Sequencing was conducted on a NovaSeq 6000 platform with PE150. Quality control and analysis were conducted subsequently.

2. *In Vivo* Studies

2.1 Rats

The study adhered to the relevant guidelines and regulations of the Shanghai Medical Ethics Committee, followed the principles of animal welfare ethics, and was approved by the Shanghai Tenth People's Hospital Ethics Committee. Histological staining was conducted with six replicates, and WB was conducted with four replicates.

2.2 Modeling of exercise-induced tendon injury

Rats were randomly divided into three groups for treadmill exercise experiments: control, low-intensity exercise (L-tm), and high-intensity exercise (H-tm). All rats underwent adaptive pre-training under conditions of 5 m/min speed, a 10° incline, 10 minutes/day for one week. The control group ceased treadmill exercise after pre-training. L-tm rats exercised at a speed of 17 m/min, a 10° incline, with a cumulative daily duration of 1.5 hours (30-minute exercise followed by a 30-minute rest with food and water), six days per week. H-tm rats exercised at a speed of 25 m/min, a 15° incline, with the same schedule as L-tm. Rats refusing to exercise were subjected to brief 0.4 mA electrical stimulation. Rats with prolonged refusal or severe lower-limb injury or dead rats were excluded. Tissue samples were collected under anesthesia after 6, 8, 12, and 18 weeks of exercise for corresponding histological assessments.

2.3 Modeling of trauma-induced tendon injury

Rats were randomly divided into three groups, including sham, tendon injury (Td-Inj) group, and tendon injury with suture (Td-Sut) group. Rats were anesthetized, and the procedures were conducted under a sterile ultraclean bench after inducing deep anesthesia. In the prone position, the rats were fixed, and the skin on the Achilles tendon was shaved and disinfected. A 0.5-1 cm incision was made on the mid-external side of the Achilles tendon to expose the tendon. For the sham group, skin suturing and disinfection were immediately conducted. In the Td-Inj group, after exposing the tendon, a partial transection (1/3–1/2 depth) was made at the midsection of the tendon in a vertical direction, followed by skin suturing with 4-0 absorbable sutures and disinfection. Following the same injury protocol as the Td-Inj group, the injured tendon was repaired in the Td-Sut group using a modified Kessler method with sterile 5-0 non-absorbable sutures. Next, the skin was then sutured and disinfected. Rats were maintained under standard conditions, and daily skin disinfection was conducted for three consecutive postoperative days. Starting one week before tissue sampling, rats in all groups received daily intraperitoneal injections of EdU solution (G5059, Servicebio) at a concentration of 0.5 mg/mL and a dose of 10 μL/g body weight. Live vascular assessments and tissue sampling were conducted on postoperative days 3, 7, 14, 28, and 42.

2.4 Modeling of tendon injury in rats with *YAP1* knockdown/overexpression

The study included four groups, including Ctl/*NC*, Inj/*NC*, Inj/$YAP1^{KD}$, and Inj/$YAP1^{OE}$ groups. Rats were anesthetized, and the skin was prepared for injection. Using a microinjector, AAV vectors (Supplementary Table 1) were subcutaneously injected into the Achilles tendon area. The Ctl/*NC* and Inj/*NC* groups received 25 μL of AAV-*NC* empty vector (1.25×10^{11}VG/rat, Shanghai Genomeditech Co., Ltd.); the

Inj/$YAP1^{KD}$ group received 25 μL of AAV-$YAP1^{KD}$ vector (1.25×10^{11}VG/rat, Shanghai Genomeditech Co., Ltd.); and the Inj/$YAP1^{OE}$ group received 25 μL of AAV-$YAP1^{OE}$ vector (1.25×10^{11}VG/rat, Shanghai Genomeditech Co., Ltd.). Rats were maintained under standard conditions after the injections.

After six weeks of transfection, the Ctl/*NC* group underwent sham modeling as described in Data file S1-2.3, while the Inj/*NC*, Inj/$YAP1^{KD}$, and Inj/$YAP1^{OE}$ groups underwent tendon injury modeling following the Td-Inj protocol (Data file S1-2.3). Tissue sampling and assessments were conducted on days 3, 7, 14, 28, and 42 after modeling.

2.5 Transmission electron microscope

Fresh tendon tissue samples approximately 1 mm^3 in size were collected and fixed in an electron microscope fixative (G1102, Servicebio) in the dark. The samples were dehydrated and infiltrated for embedding (acetone, 10000418, Sinopharm Chemical Reagent Co., Ltd.; 812 embedding agent, 90529-77-4, SPI). The embedding blocks were polymerized in a 60°C oven for 48 hours. Ultra-thin sections (with 60-80 nm thickness) were prepared using an ultra-microtome (cross-sections of the middle region of tendon injuries). The sections were then stained with 2% uranyl acetate saturated ethanol solution in the dark and 2.6% lead citrate solution without exposure to carbon dioxide, followed by washing and drying. A transmission electron microscope was used for observation, and images were captured for analysis.

2.6 Histopathological assessment

Rat tendon tissue samples were processed for paraffin embedding, followed by HE, SR, and TUNEL staining based on the procedures and analysis methods described in Data file S1-1.2. Other staining methods included Masson's trichrome staining (Masson) and Safranin O-Fast green staining (SOFG), as well as IHC, IF, and WB. The detailed protocols for these staining and analysis techniques are provided below.

Masson: Masson staining was conducted using a staining kit (G1006, Servicebio). Sections were sequentially immersed in Masson solutions A, B, C, D, E, and F, followed by differentiation with 1% glacial acetic acid, dehydration, and mounting. Images were captured and ImageJ software was used to calculate the loose collagen volume fraction (%LCVF = blue-stained area/total collagen area). The average value from four fields of view was regarded as the sample score. Masson staining is based on tissue permeability: tightly packed tissues with low permeability stain red, while loose tissues with high permeability stain blue. In tendon staining, tightly packed and “mature” collagen bundles stain red, while “fresh and immature” loose collagen fibers stain blue, reflecting tissue remodeling/maturation.

SOFG: SOFG staining was conducted using a Safranin O-Fast green staining kit (G1053, Servicebio), where cartilages were stained red.

IHC: IHC staining was conducted for IL-1β (GB11113, Servicebio), IL-6 (TD6087, Abmart), TNF-α (PY19810, Abmart), MMP2 (GB11130, Servicebio), MMP3 (GB11131, Servicebio), MMP9 (GB15132, Servicebio), VEGFA (TA5131, Abmart), CD34 (GB15013, Servicebio), FHL2 (TD13015, Abmart), YAP1 (A19134, ABclonal), sFRP2 (TD4451, Abmart), and p-$YAP1^{S127}$ (TA3328, Abmart). The procedures and analysis methods followed those described in Data file S1-1.2.

IF: IF staining, including single-label or multiplex, was conducted for *Ki-67*$^+$, *TGF-β1*$^+$, *Tnmd*$^+$*CD90*$^+$*CD44*$^+$, *CD34*$^+$*PLCB1*$^+$*ATF6*$^+$, *sFRP2*$^+$*TnC*$^+$, and *YAP1*$^+$*TnC*$^+$ cells. The procedures and analysis methods are described in Data file S1-1.2. Differently, ImageJ software was used to calculate the mean fluorescence intensity for *TGF-β1*$^+$ IF staining (mean gray value).

In the trauma-induced tendon injury model, proliferating cells in the tissue were pre-labeled with EdU, and the proportion of *EdU*$^+$*FHL2*$^+$*TnC*$^+$, *EdU*$^+$*sFRP2*$^+$*TnC*$^+$, *EdU*$^+$*YAP1*$^+$*TnC*$^+$, and *EdU*$^+$*sFRP2*$^+$*vWF*$^+$ cells was meausred. Paraffin sections were deparaffinized to water, followed by antigen retrieval using Tris-EDTA antigen retrieval buffer (pH=8.0, G1207, Servicebio). Tissue sections were treated with 3% hydrogen peroxide, blocked with 3% BSA solution (or 10% rabbit serum), and incubated with the corresponding primary and secondary antibodies (HRP-labeled). After treatment with TSA staining reagents (G1226, Servicebio), a second round of incubation with primary and secondary antibodies was conducted. Subsequently, 60 μL of the EdU staining reaction mixture (including reaction buffer, catalyst A, fluorescent dye B, and catalyst additive C, G1603, Servicebio) was added to each slide and dark incubated at room temperature for 30 minutes. Finally, the sections were counterstained with DAPI, autofluorescence quenched, and mounted for analysis. Images were captured and analyzed following the methods described in Data file S1-1.2. Primary antibodies were as follows: Ki-67 (GB111499, Servicebio), TGF-β1 (A22296, ABclonal), Tnmd (PC41215, Abmart), CD90 (ab307736, Abcam), CD44 (T55122, Abmart), CD34 (GB15013, Servicebio), PLCB1 (TD6726, Abmart), ATF6 (TD6009, Abmart), FHL2 (TD13015, Abmart), YAP1 (A19134, ABclonal), sFRP2 (TD4451, Abmart), TnC (A18156, ABclonal), and vWF (ab6994, Abcam).

WB: WB of rat tendons was conducted for the following indicators: FHL2 (TD13015, Abmart), YAP1 (A19134, ABclonal), p-YAP1^{S127} (TA3328, Abmart), sFRP2 (TD4451, Abmart), CTGF (A11067, ABclonal), MST1 (A8043, ABclonal), p-MST1^{T387} (AP0906, ABclonal), LATS1 (A17992, ABclonal), p-LATS1/2$^{Ser909/Ser872}$ (TA8163, Abmart), Col-I (ab270993, Abcam), Col-III (TA5457, Abmart), Tnmd (PC41215, Abmart), VEGFA (TA5131, Abmart), ATF6 (TD6009, Abmart), TGF-β1 (A22296, ABclonal), and GAPDH (GB15002, Servicebio). The experimental and analytical methods followed those described in Data file S1-1.2.

3. *In Vitro* Studies

3.1 siRNA/plasmid-treated tenocyte models

Tenocytes transfected with siRNA or overexpression plasmids were cultured in T25 flasks (approximately 2~5×10^5 cells). Opti-MEM was used to prepare the transfection solutions for siRNA (50 nM/T25; *siNC*, *siYAP1*, *siFHL2*; Supplementary Table 2; Shanghai Genomeditech Co., Ltd.) and overexpression plasmids (4 μg/T25; *Vectors*, *FHL2*^{OE}; Supplementary Table 2; Shanghai Genomeditech Co., Ltd.) at a volume of 500 μL/T25: Opti-MEM + siRNA/plasmid + 7.5 μL Lipo3000 (with an additional 10 μL P3000 for plasmid transfection)/T25. The solution was incubated at room temperature for 20 minutes. Primary cells seeded beforehand (switched to antibiotic-free medium 24 hours prior) were washed to remove the previous medium

and were treated with the transfection solution, followed by the addition of 3.5 mL of antibiotic-free complete medium with 10% FBS. After 48 hours of incubation, the medium was replaced with a standard complete medium to establish activated tenocyte models.

After transfection, tenocytes were treated with 2.5 ng/mL IL-1β, 0.1 μM tBHP, or 4 ng/mL TGF-β1 for 24 hours, and then WB or flow cytometry apoptosis assay was conducted. The experimental groups were as follows: the *siNC* group (transfected with *siNC* without any stimulation), the IL-1β/tBHP/TGF-β1+*siNC* group (transfected with *siNC* followed by treatment with IL-1β, tBHP, or TGF-β1, respectively), the *siYAP1* group (transfected with *siYAP1* without any stimulation), the IL-1β/tBHP/TGF-β1+*siYAP1* group (transfected with *siYAP1* followed by treatment with IL-1β, tBHP, or TGF-β1, respectively), the *siFHL2* group (transfected with *siFHL2* without any stimulation), the IL-1β/tBHP/TGF-β1+*siFHL2* group (transfected with *siFHL2* followed by treatment with IL-1β, tBHP, or TGF-β1, respectively), the *vector* group (transfected with *vectors* without any stimulation), the IL-1β (2.5 and 5 ng/mL)/tBHP/TGF-β1+*vector* group (transfected with *vectors* followed by treatment with IL-1β, tBHP, or TGF-β1, respectively), the $FHL2^{OE}$ group (transfected with $FHL2^{OE}$ without any stimulation), and the IL-1β (2.5 and 5 ng/mL)/tBHP/TGF-β1+$FHL2^{OE}$ group (transfected with $FHL2^{OE}$ followed by treatment with IL-1β, tBHP, or TGF-β1, respectively).

3.2 Enzyme-linked immunosorbent assay (ELISA)

ELISA was conducted using tenocytes cultured in T75 flasks until reaching 60%-70% confluence. Transfection with *siNC* or *siYAP1* and treatments with IL-1β, tBHP, or TGF-β1 were conducted as described in Data file S1-3.1, maintaining consistent volume ratios (four replicates per group). The following groups were included: *siNC*, IL-1β+*siNC*, tBHP+*siNC*, TGF-β1+*siNC*, IL-1β+*siYAP1*, tBHP+*siYAP1*, TGF-β1+*siYAP1*, and *siYAP1* groups. Approximately 12 mL of the culture medium was collected from each group, centrifuged and concentrated using ultrafiltration tubes (UFC901096, Millipore) to obtain approximately 1 mL of the concentrate. ELISA was conducted to detect sFRP2 (six replicates per sample, CSB-EL021139HU, CUSABIO). The final standard curve equation was (r=0.99994): y=(a+bx)/(1+cx+dx^2), where a=-0.20327, b=1.62832, c=-0.32650, and d=0.02186. The sFRP2 concentration (ng/mL) in the concentrated samples was calculated accordingly.

3.3 Tenocyte-HUVECs co-culture models

$sFRP2^{KD}$ tenocytes were produced using *siSFRP2* transfection (Supplementary Table 2) following the method described in Data file S1-3.1. Combined with IL-1β treatment (2.5 ng/mL, 24 h), WB was conducted to measure sFRP2 expression and validate the reliability of the model.

HUVECs were seeded in the lower Transwell chamber (0.4 μm pore size, CLS3412, Corning), while normal tenocytes or $sFRP2^{KD}$ tenocytes were seeded in the upper chamber. Endothelial cell complete medium (HUVEC-90011, OriCell), with or without IL-1β (2.5 ng/mL), was used for cultivation. The groups included normal tenocytes+HUVECs, IL-1β-treated normal tenocytes+HUVECs, and IL-1β-treated

sFRP2^{KD} tenocytes+HUVECs. After 24 hours of co-culturing, HUVECs from the lower chamber were collected for tube formation assay. Following 24 hours of tube formation, the medium was removed from the wells, and 50 μL of Calcein AM working solution (diluted with serum-free medium; C2012, Beyotime) was added. The samples were dark incubated for 30 minutes, washed with PBS, and images were captured at 494 nm/514 nm for IF. ImageJ software was employed to determine the number of branches, total branching length, number of junctions, and number of meshes.

3.4 Scratch wound healing assay

Parallel lines were drawn on the outer bottom surface of a 6-well plate using a marker, with a spacing of nearly 0.5 cm to ensure consistent imaging locations. HUVECs were seeded in the 6-well plate. After reaching the logarithmic growth phase (80%-90% confluence), the plate was removed from the incubator. The medium was aspirated, and a scratch was made along the horizontal lines on the plate bottom using a 10 μL pipette tip, maintaining the tip perpendicular to the surface without tilting. The same procedure was applied to each well. The cells were rinsed with PBS three times (2 mL per rinse) to remove the detached cells. Subsequently, the medium containing 2% FBS and the respective drug concentrations was added, and the plate was returned to the incubator for cultivation. Observations and imaging were conducted at 0, 12, 24, and 48 hours. Cell migration rate = (initial width at 0 h – width at a specific time point) / initial width at 0 h.

4. Therapeutic Study

4.1 AAV-*FHL2*^{OE} therapeutic intervention in the rat models

Twelve-week-old male Sprague-Dawley rats (specific pathogen-free, Shanghai Jihui Laboratory Animal Care Co., Ltd.) were used for this experiment. Tendon injury and tendon injury suture models were established as described in Data file S1-2.3. AAV transfection was conducted on day 3 after modeling (Supplementary Table 1, Shanghai Genomeditech Co., Ltd.) including the following groups: Ctl/*EGFP* group (no tendon injury + AAV-*EGFP* transfection (25 μL, 5.6×10^{11}VG)), Ctl/*EGFP-FHL2*^{OE} group (no tendon injury + AAV-*EGFP-FHL2*^{OE} transfection (25 μL, 5.6×10^{11}VG)), Inj/*EGFP* group (tendon injury + AAV-*EGFP* transfection (25 μL, 5.6×10^{11}VG)), Inj/*EGFP-FHL2*^{OE} group (tendon injury + AAV-*EGFP-FHL2*^{OE} transfection (25 μL, 5.6×10^{11}VG)), Sut/*EGFP* group (tendon injury suture + AAV-*EGFP* transfection (25 μL, 5.6×10^{11}VG)), and Sut/*EGFP-FHL2*^{OE} group (tendon injury suture + AAV-*EGFP-FHL2*^{OE} transfection (25 μL, 5.6×10^{11}VG)). OCTA, vascular perfusion, and histopathological assessments were conducted at 6, 8, and 10 weeks after transfection. Evaluation methods for OCTA and histopathology (HE, Masson, IHC, WB) are described in Data file S1-2.6.

IF staining for frozen tissue sections was conducted to analyze the efficiency of AAV transfection (EGFP, 6 weeks). Fresh tendon tissues were embedded in OCT (G6059, Servicebio) and rapidly frozen, followed by sectioning (8-10 μm). DAPI was used for nuclear staining, and the slides were mounted. The images were acquired with the excitation wavelength of 330-380 nm and emission wavelength of 420 nm

for DAPI, and the excitation wavelength of 484 nm and emission wavelength of 507 nm for EGFP.

Supplementary Figures

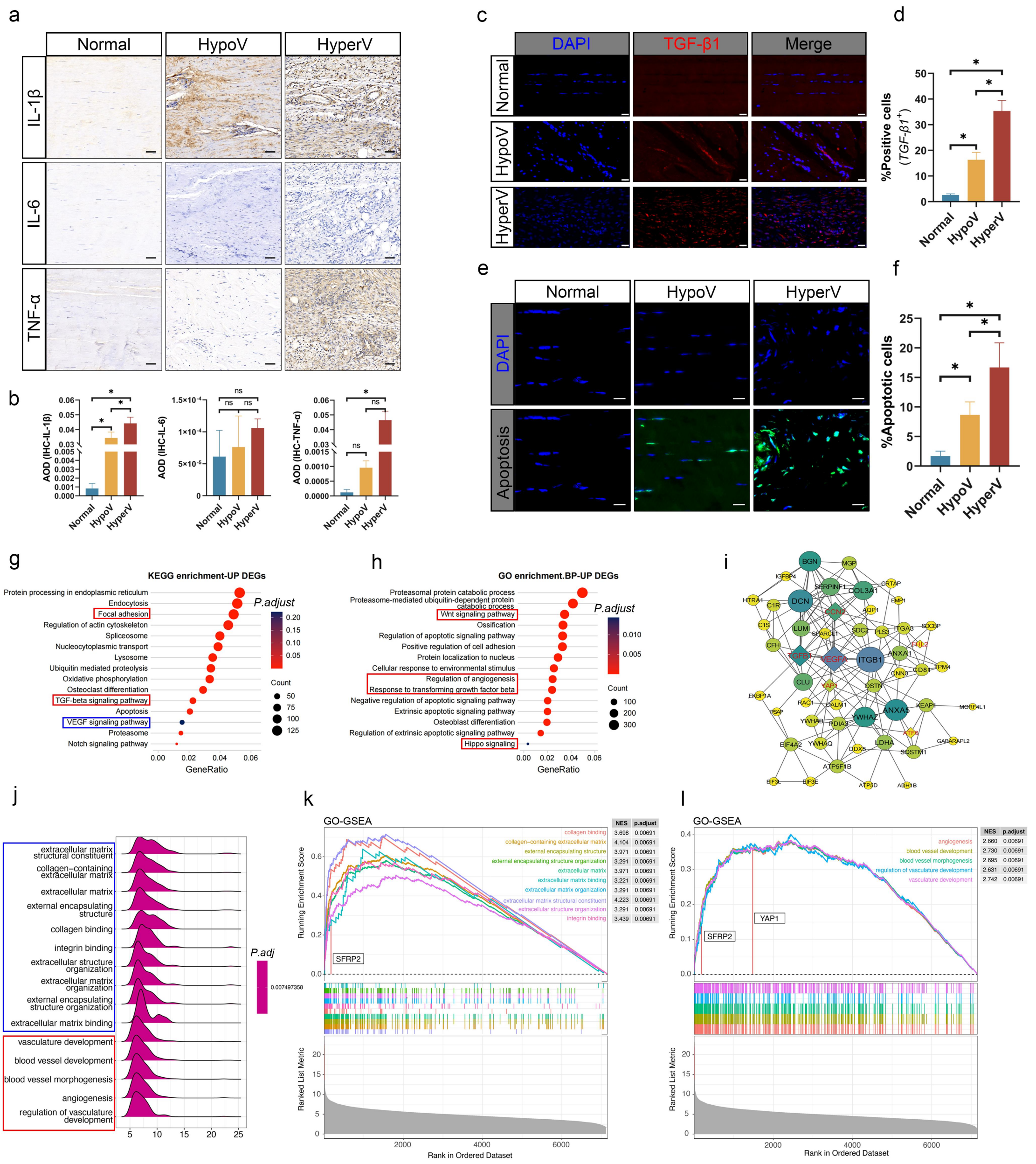

Supplementary Fig. 1. Bulk RNA-seq analysis of the HyperV and HypoV groups. (**a**) Immunohistochemical (IHC) staining for inflammatory factors, including IL-1β, IL-6, and TNF-α, scale bar = 50 μm. (**b**) Average optical density (AOD) of IL-1β, IL-6, and TNF-α in IHC staining. (**c**) TGF-β1 immunofluorescence staining, scale bar=20 μm. (**d**) The percentages of $TGF\text{-}\beta 1^{+}$ cells. (**e**) TUNEL staining for apoptosis, scale bar =20 μm. (**f**) The percentage of apoptotic cells. (**g,h**) Kyoto Encyclopedia of Genes and Genomes (KEGG)/Gene Ontology (GO) enrichment of upregulated differentially expressed genes (UP-DEGs, HyperV vs. HypoV), with red boxes highlighting key pathways. (**i**) Protein-protein interaction network of relevant DEGs. (**j**) Ridge plot of gene set enrichment analysis (GSEA, GO database), with blue boxes indicating matrix remodeling-related pathways and red boxes showing

angiogenesis-related pathways. (**k**) GSEA line chart for matrix remodeling-related pathways. (**l**) GSEA line chart for angiogenesis-related pathways. *$P<0.05$; ns $P>0.05$.

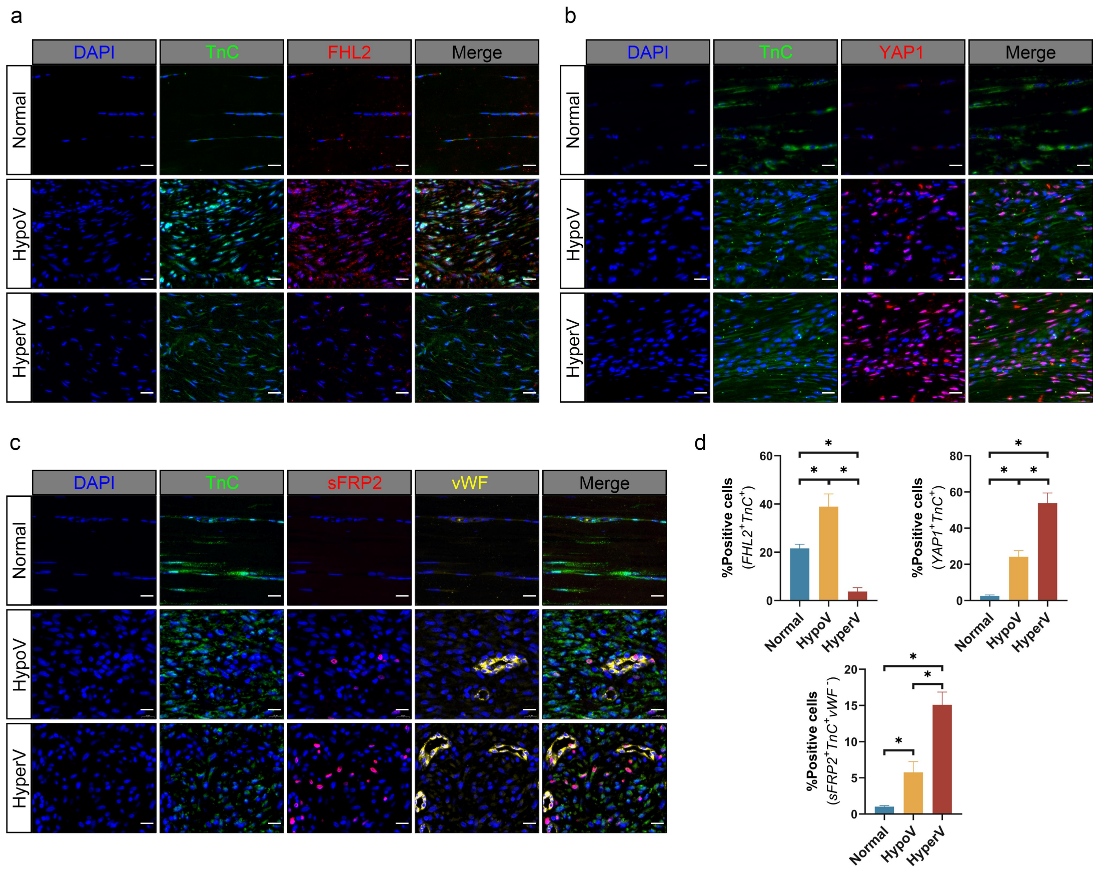

Supplementary Fig. 2. Localization of the protein expression of FHL2, YAP1, and sFRP2. (**a**) Double immunofluorescence (IF) staining of FHL2 and TnC. (**b**) Double IF staining of YAP1 and TnC. (**c**) Triple IF staining of sFRP2, TnC, and vWF. (**d**) Statistical analysis of $FHL2^{+}TnC^{+}$, $YAP1^{+}TnC^{+}$, and $sFRP2^{+}TnC^{+}vWF^{-}$ positive cell percentages. *$P<0.05$; Scale bar = 20 μm.

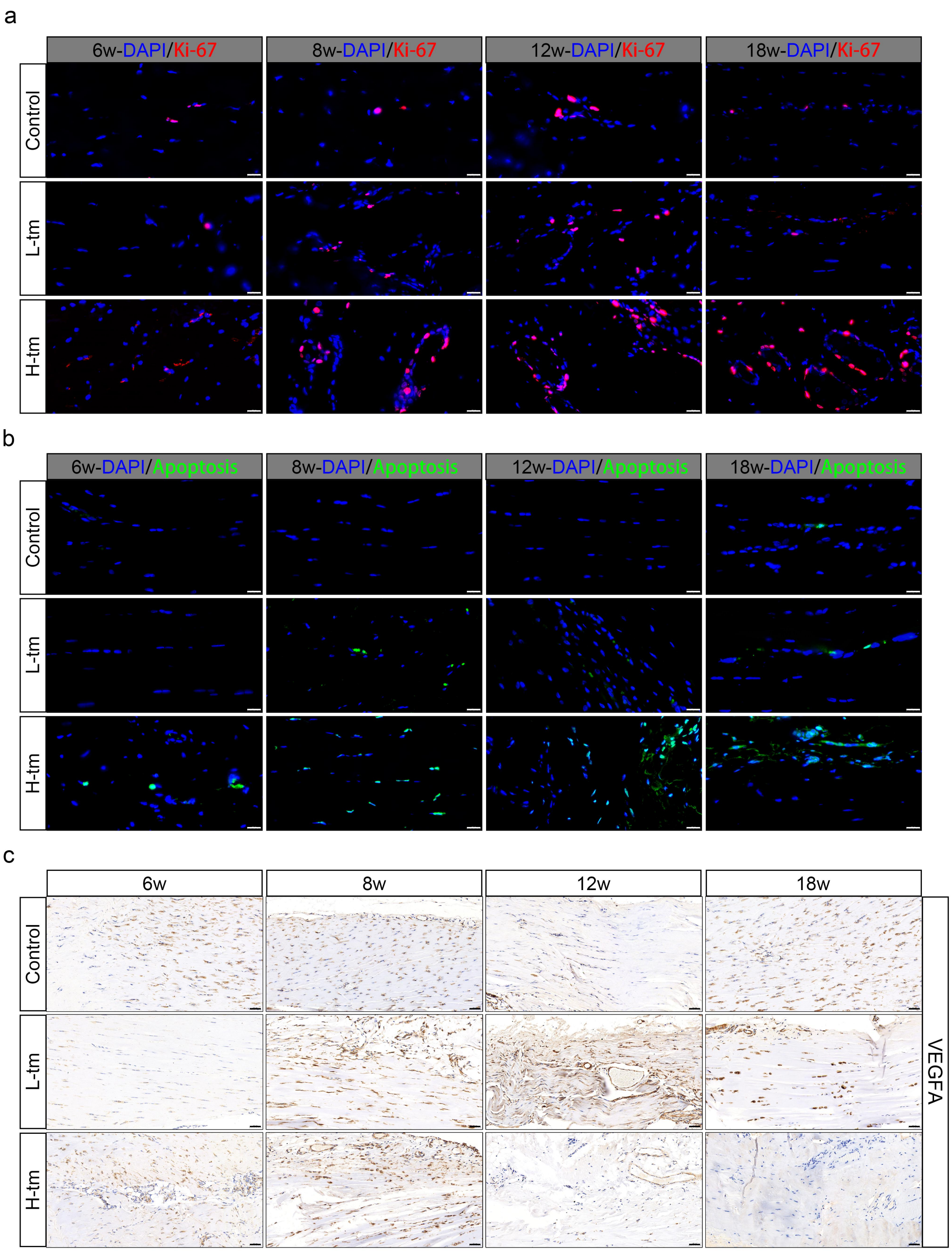

Supplementary Fig. 3. Cell proliferation, apoptosis, and VEGFA expression in the control, L-tm, and H-tm groups. (**a**) Ki-67 immunofluorescence staining (Scale bar=20 μm). (**b**) TUNEL assay (Scale bar=20 μm). (**c**) The immunohistochemical staining for VEGFA (Scale bar=50 μm).

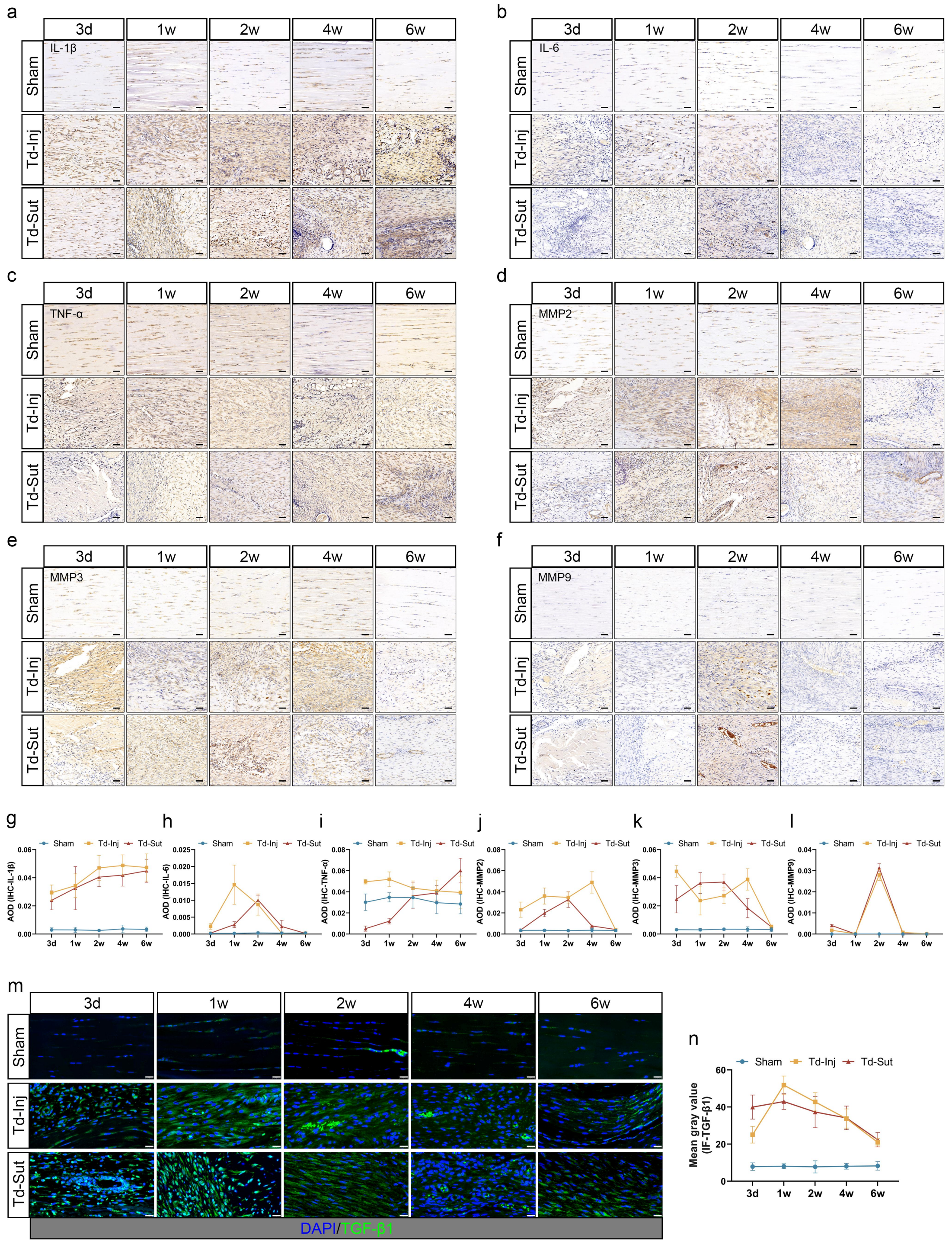

Supplementary Fig. 4. Evaluation of matrix remodeling in the Sham, Td-Inj, and Td-Sut groups. (**a-c**) The immunohistochemical (IHC) staining for inflammatory factors (IL-1β, IL-6, and TNF-α, Scale bar=50 μm). (**d-f**) IHC staining for MMPs (MMP2, MMP3, and MMP9, Scale bar=50 μm). (**g-l**) The average optical density (AOD) for inflammatory factors (IL-1β, IL-6, and TNF-α) and MMPs (MMP2, MMP3, and MMP9) in the sham, Td-Inj, and Td-Sut groups (IHC staining). (**m**) TGF-β1 immunofluorescence staining (Scale bar=20 μm). (**n**) The fluorescence intensity of TGF-β1 (mean gray value).

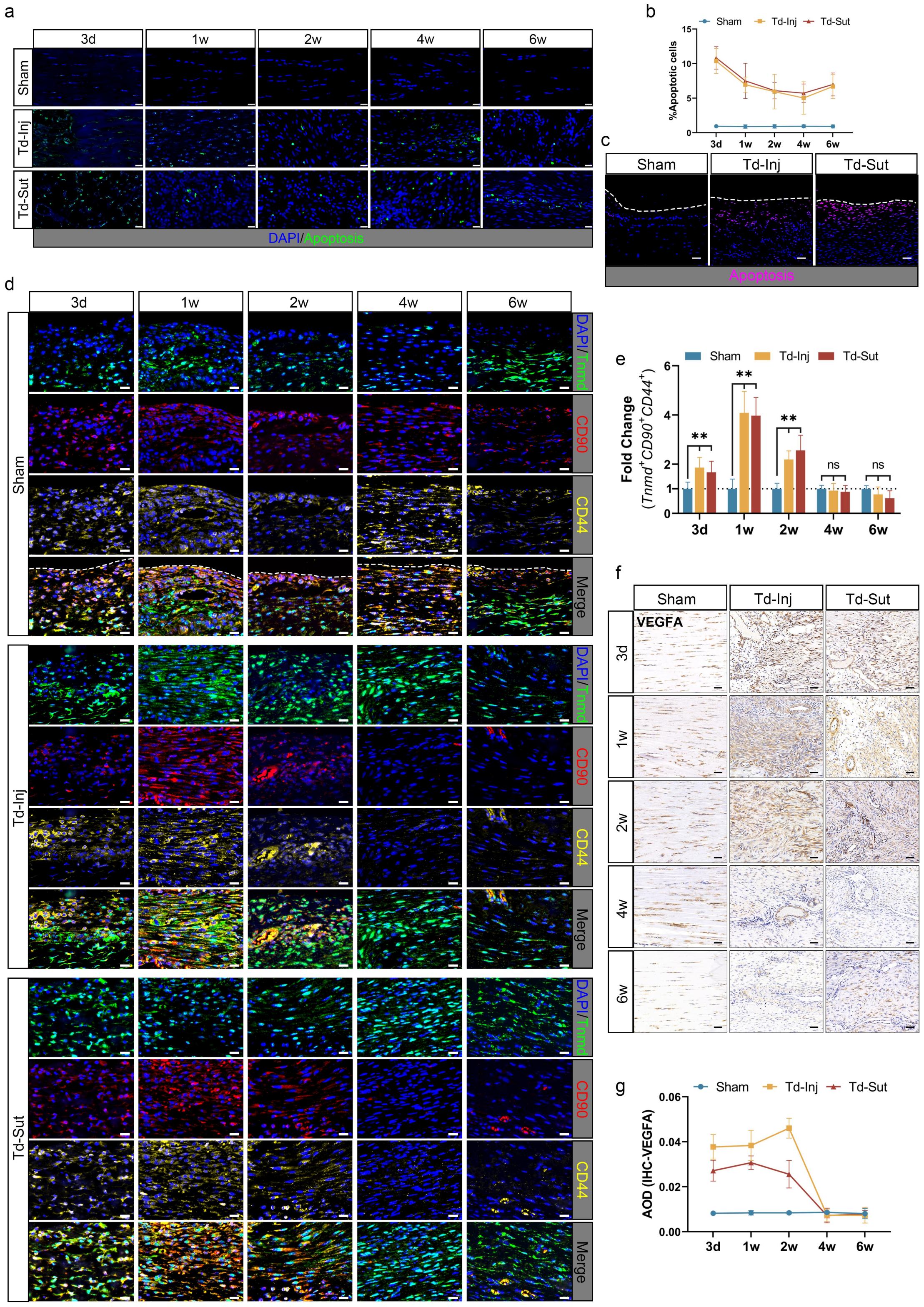

Supplementary Fig. 5. Evaluation of apoptosis and tendon stem/progenitor cells in the Sham, Td-Inj, and Td-Sut groups. (**a**) TUNEL assay (Scale bar=20 μm). (**b**) The percentages of apoptotic cells (TUNEL). (c) TUNEL staining, showing apoptotic cells concentrated at the tendon sheath boundary, with dashed lines marking tendon edges (Scale bar=50 μm). (**d**) The immunofluorescence (IF) staining of

Tnmd$^+$*CD90*$^+$*CD44*$^+$ tendon stem cells, with dashed lines marking tendon edges (Scale bar=20 μm). (**e**) The proportions of *Tnmd*$^+$*CD90*$^+$*CD44*$^+$ cells (fold to sham, ***P*<0.05 vs. Sham, ns *P*>0.05). (**f**) The immunohistochemical (IHC) staining for VEGFA (Scale bar=50 μm). (**g**) The average optical density (AOD) for VEGFA in IHC staining.

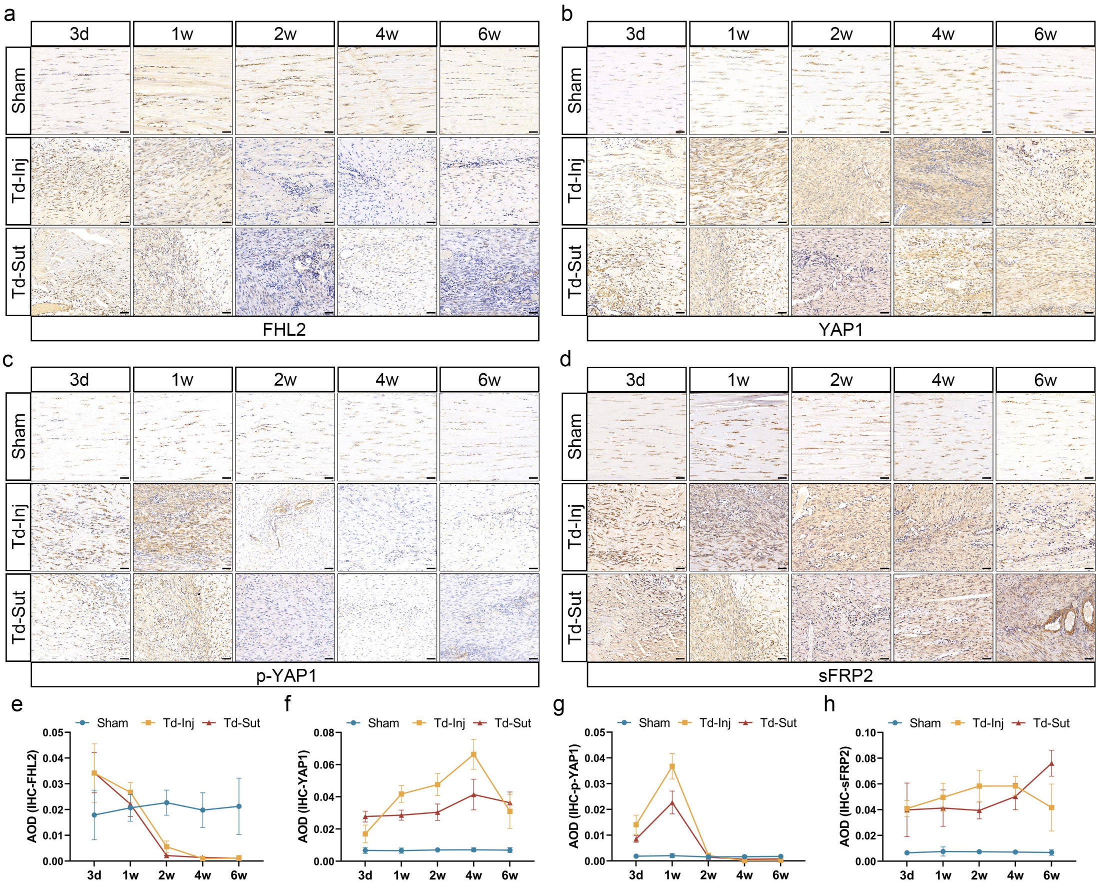

Supplementary Fig. 6. The expression levels of key proteins FHL2/YAP1/sFRP2. (**a**) Immunohistochemical (IHC) staining for FHL2. (**b**) IHC staining for YAP1. (**c**) IHC staining for p-YAP1. (**d**) IHC staining for sFRP2. (**e-h**) Statistical charts of average optical density (AOD) for FHL2, YAP1, p-YAP1, and sFRP2 in IHC staining. Scale bar=50 μm.

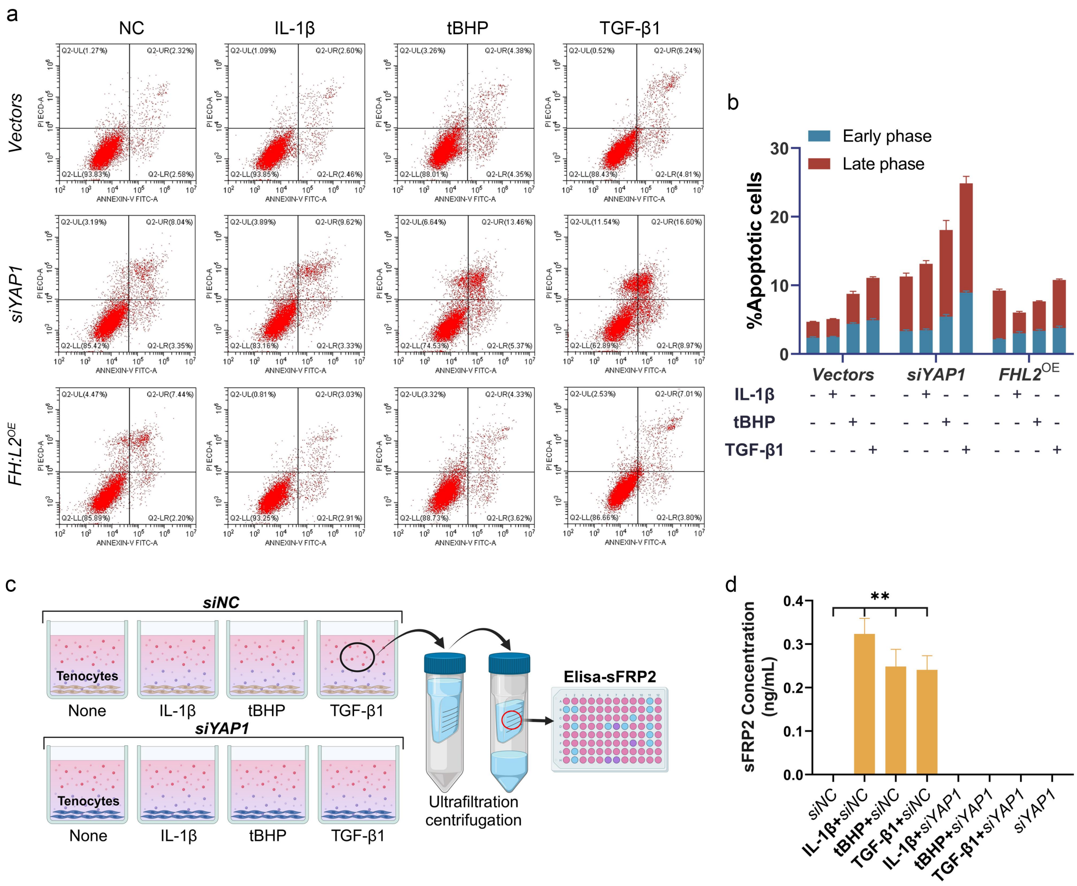

Supplementary Fig. 7. Apoptosis analysis and extracellular secretion of sFRP2 in activated tenocytes treated with *siYAP1*/*FHL2*^{OE}. (**a**) Flow cytometry analysis for apoptosis in tenocytes transfected with *Vectors*/*siYAP1*/*FHL2*^{OE} and treated with IL-1β (2.5 ng/mL), tBHP (0.1 μM), and TGF-β1 (4 ng/mL) for 24 hours; UR represents late apoptotic cells, LR represents early apoptotic cells. (**b**) Statistical chart of the percentage of early and late apoptotic cells. (**c,d**) Enzyme-linked immunosorbent assay (ELISA) for detecting sFRP2 concentration in culture media (ultrafiltration concentrate) from activated tenocytes treated with *siNC*/*siYAP1*, showing extracellular secretion of sFRP2 by activated tenocytes (***P*<0.05 vs. *siNC*).

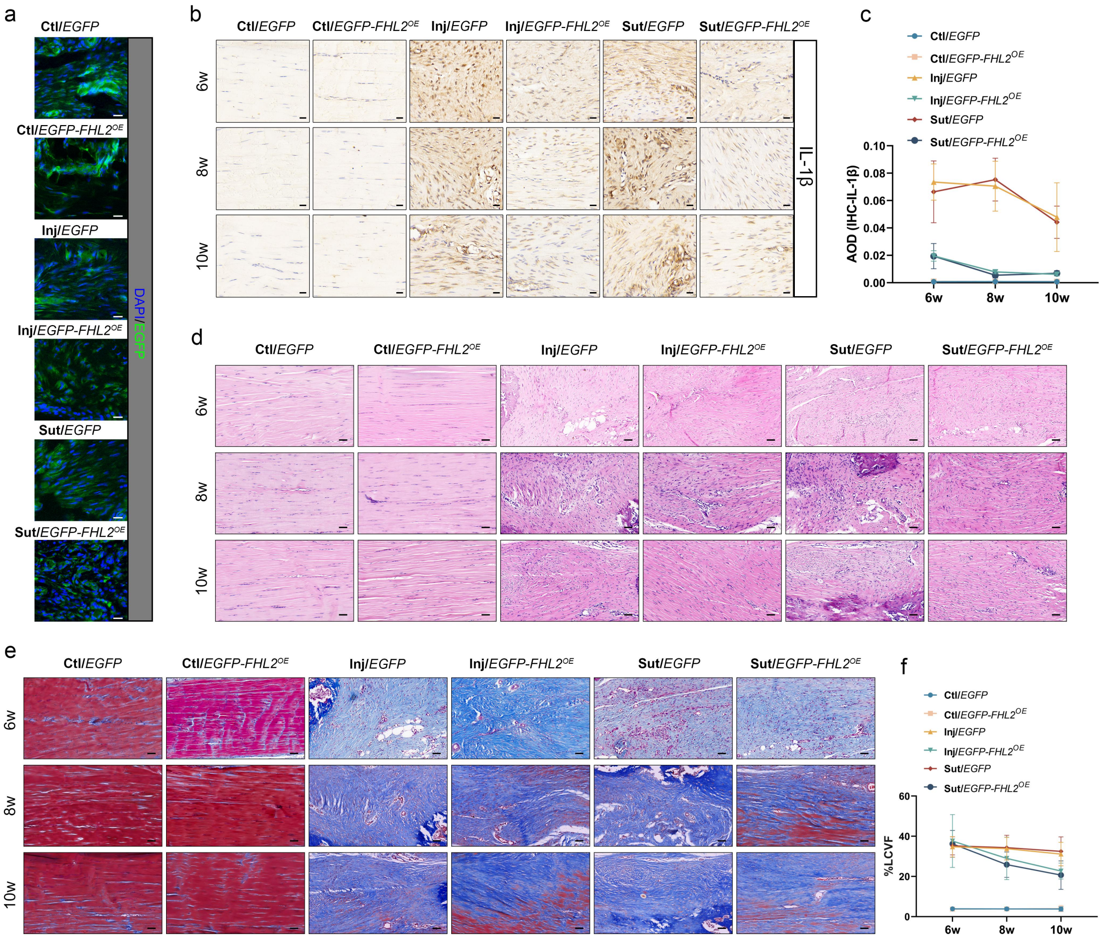

Supplementary Fig. 8. *FHL2* overexpression effectively improved tissue remodeling. (**a**) The immunofluorescence (IF) staining of frozen sections showing EGFP at 6 weeks after AAV-*EGFP* (negative control) or AAV-*EGFP-FHL2*^{OE} transfection (Scale bar=20 μm). (**b,c**) The average optical density (AOD) for IL-1β in immunohistochemical staining (Scale bar=20 μm). (**d**) Hematoxylin-Eosin staining (Scale bar=50 μm). (**e**) Masson's trichrome staining (Scale bar=50 μm). (**f**) Statistical chart of loose collagen volume fraction (%LCVF) from the Masson's trichrome staining.

Supplementary Table 1. AAV transfection systems

Transfection systems	Plasmids	Vectors
AAV-*NC* (negative control)	GPAAV-CMV-MCS-WPRE	AAV9
AAV-*YAP1*^{OE} (*YAP1* overexpression)	GPAAV-CMV-Rat_Yap-WPRE	AAV9
AAV-*YAP1*^{KD} (*YAP1* knockdown)	GPAAV-CMV-mir30-Rat_Yap1(Yap)-shRNA1-mir30-Rat_Yap1(Yap)-shRNA2-mir30-Rat_Yap1(Yap)-shRNA3-WPRE<br><br>shRNA1: GGTCAGAGATACTTCTTAAAT<br>shRNA2: GGAGAGGCTGCGATTGAAACA<br>shRNA3: GGATGGAGGGACTCAGAATGC	AAV9
AAV-*EGFP* (*EGFP*-labelled control)	GPAAV-CMV-EF1-EGFP-WPRE	AAV9
AAV-*EGFP-FHL2*^{OE} (*FHL2* overexpression)	GPAAV-CMV-Rat_FHL2-T2A-eGFP-WPRE	AAV9

Supplementary Table 2. siRNA and overexpression plasmid transfection systems

Transfection systems	Sequence (5'-3')/Information
siNC	UUCUCCGAACGUGUCACGUdTdT
	ACGUGACACGUUCGGAGAAdTdT
siYAP1	GACCAAUAGCUCAGAUCCUUU tt
	AAAGGAUCUGAGCUAUUGGUC tt
siFHL2	CCCUGCUAUGAGAAACAACAU tt
	AUGUUGUUUCUCAUAGCAGGG tt
siSFRP2	CAUCAACCGAGAUACCAAA tt
	UUUGGUAUCUCGGUUGAUG tt
Vectors	PGMLV-CMV-MCS-PGK-Blasticidin
FHL2^{OE}	PGMLV-CMV-H_FHL2-HA-PGK-Blasticidin
